# Preparation of Hydrogel by Crosslinking and Multi-Dimensional Applications

**DOI:** 10.3390/gels11110896

**Published:** 2025-11-09

**Authors:** Md Murshed Bhuyan, Jae-Ho Jeong

**Affiliations:** 1 Department of Mechanical, Smart, and Industrial Engineering (Mechanical Engineering Major), Gachon University, 1342, Seongnam-daero, Sujeong-gu, Seongnam-si 13120, Republic of Korea; 2 School of Mechanical Engineering, College of Engineering, Chung-Ang University, 84 Heukseok-Ro, Dongjak-Gu, Seoul 06974, Republic of Korea

**Keywords:** gels/hydrogel, biomedical, agriculture, electronics, edible, TEG

## Abstract

Functional hydrogels are cutting-edge materials that are important in various fields, such as biomedical engineering, agriculture, pollution control, artificial organs, electronics, and domestic products. They are essential to contemporary scientific and industrial advancements because of their adaptability and versatility. The new synthesis techniques and multidimensional applications of different kinds of hydrogels are the goals of this study. The special qualities of hydrogels are one of the main reasons for their widespread use. Because of their stimulus-responsivity, these materials may alter their properties in response to external environmental signals, including light exposure, pH, and temperature. Their biodegradability and biocompatibility make them appropriate for ecological and medicinal applications, while their intrinsic flexibility guarantees adaptation across many applications. Furthermore, the ability of hydrogels to self-heal and be reused enhances their sustainability and efficiency. The preparation of hydrogels with these unique qualities necessitates exacting preparation methods and cautious raw material selection based on the application. To improve their operation and make sure they satisfy the required performance standards in various sectors, a variety of chemical and physical modifications are used. The functional processes of hydrogels in each sector are thoroughly examined in this review, which offers in-depth information on their interactions, efficacy, and the science underlying their uses. By providing a comprehensive overview, this analysis hopes to provide readers with a solid knowledge of potential hydrogels, empowering them to investigate new avenues for research and optimize their uses across a range of sectors.

## 1. Introduction

Hydrogels, as three-dimensional polymeric networks, have emerged as a promising solution to address various challenges in fields such as electronic devices, drug delivery, biomedical engineering, agriculture, and water purification [1]. Their unique properties, including high water content, biocompatibility, and tunable mechanical strength, make them suitable for diverse applications [2]. However, several challenges persist in hydrogel synthesis and application, including mechanical stability, reproducibility, biointegration, and multifunctionality. To overcome these limitations, researchers have focused on innovative hydrogel designs that incorporate new materials and advanced synthesis techniques. Hydrogels can be synthesized using a variety of raw materials, including natural and synthetic polymers, monomers, graphene, carbon nanotubes, and other organic-inorganic compounds [3]. The selection of raw materials depends on the specific application requirements, ensuring optimal performance and adaptability. While synthetic hydrogels offer durability and tunability, they frequently present end-of-life issues because of low biodegradability and increased life-cycle effects. Natural polymer hydrogels are typically more biodegradable and ecologically benign [4]. Hydrogels are made of natural polymers, such as cellulose, alginate, chitosan, and gelatin. They exhibit high biodegradability due to microbial and enzymatic breakdown processes. Usually non-toxic, breakdown products reintegrate into natural processes. For instance, chitosan hydrogels break down into glucosamine, which is harmless to the environment [5]. Synthetic polymer hydrogels, such as polyvinyl alcohol (PVA), polyethylene glycol (PEG), and polyacrylamide, often decompose slowly or are not biodegradable. Ecological issues may arise from the production of hazardous byproducts or microplastics during degradation. To improve end-of-life results, some more recent synthetic designs include degradable connections (such as ester and disulfide bonds). However, hydrogels made of synthetic and natural polymers have different sustainability characteristics. Natural polymers often offer intrinsic biodegradability and environmental friendliness, despite their limited mechanical strength and uniformity. Despite the environmental concerns associated with synthetic polymers in the past, designers are increasingly focusing on their biodegradability and specific functions, offering customizable features and a wider range of applications [6].

Preparation techniques for hydrogels face challenges that influence their quality and functionality. Acrylic acid, for instance, tends to dimerize into diacrylic acid (DAA), which can negatively impact the hydrogel’s characteristics by raising the soluble fraction and residual monomer content—two things that are not desirable in the finished result. In this case, methoxyhydroquinone (MHC) and other inhibitors are utilized to stop acrylic acid from spontaneously polymerizing while being stored. But, in commercial production, these inhibitors are usually not eliminated, which might result in the development of undesirable dimers that need to be controlled. Temperature, water concentration, and pH levels all affect how quickly DAA forms. For every 5 °C rise in temperature, the dimerization rate doubles. This indicates that in order to reduce DAA formation and guarantee a high-quality hydrogel, careful management of these parameters is necessary [7]. A rubbery reaction product that can be challenging to handle is frequently the consequence of the production process. Problems include an uneven distribution of particle sizes and an increase in sol content because of heat and hydrolytic cleavage. These elements can impact the performance of the finished product and make the production process more difficult. Therefore, process optimization is essential for producing hydrogel. This covers the categorization of hydrogels according to their chemical and physical properties, as well as the viability of using them technically. To properly address these issues, industrial technology innovations and their consequences for process design are important [8,9]. However, the radiation technique has advantages over other conventional methods of hydrogel preparation, such as no cross-linking agent or initiator is required, simple processing, and comparatively pure gel production [10]. The functional groups—-COOH, -OH, -NH_2_, -NO_2_, -N(CH_3_)_2_, -SO_3_H, -PO_3_H, -CONH_2_, -CON(CH_3_)_2_, etc.—determine the properties and application of hydrogels [11,12]. The most significant properties of hydrogels are swelling-deswelling capacity, mechanical strength, stimuli-responsivity, self-healing, biological mimicking, and reusability [13]. By tuning the above properties, hydrogel can be applied in the desired sector, for example, in drug delivery with a controlled release rate, selective adsorption of metal ions, and soft electronics [14,15]. The recent and revolutionary application of hydrogel is in electronic devices and biomedical engineering. Abhishek Paudel et al. reported a metal free hydrogel-based battery where ammonium cations (NH_4_^+^) act as charge carriers, polyaniline (PANI), polypyrrole (PPy), and edible xanthan gum work as anode, cathode, and electrolyte, respectively [16]. Rongrong Zhao et al. successfully developed a mechanically flexible, bio-adhesive, self-healing, and biocompatible hydrogel by combining poly(3,4 ethylenedioxythiophene): polystyrene sulfonate (PEDOT:PSS) with guar gum that has the potential to be utilised in a variety of wearable devices, including smart systems for sign language recognition and sensitive skin sensors for tracking human movements [17].

Hydrogels, despite their promising applications in various biomedical and engineering fields, encounter several challenges that hinder their effectiveness. Their low solubility, high crystallinity, and non-biodegradability significantly impact performance, making processing difficult and limiting their usability. A major obstacle in hydrogel development is the lack of comprehensive knowledge surrounding the intricate processes of swelling and water absorption. This gap in understanding complicates the design of hydrogels tailored for specific applications, as researchers struggle to optimize their mechanical, chemical, and physical properties effectively [18]. A notable advancement in hydrogel technology is the development of ionic liquid hydrogels, which provide a stable and adaptable medium for protein crystallization and other biochemical processes. These materials have opened new avenues in materials science, biochemistry, and structural biology, offering enhanced control over molecular interactions. A pioneering study by Qi Han et al. introduced injectable hydrogels composed of alginate and ionic liquid ethylammonium nitrate, designed specifically to improve protein crystallization [19]. Through their research, they successfully crystallized lysozyme in these hydrogels, demonstrating that the resulting crystals maintained their stability and shared similar properties with those observed in liquid environments. This innovation represents a breakthrough in hydrogel applications, paving the way for improved sample transport and optimized crystal formation for various biological and pharmaceutical processes. By addressing hydrogel limitations and expanding their potential through ionic liquid-based modifications, researchers can unlock new possibilities in drug delivery, tissue engineering, and biotechnology [19]. The review explores different hydrogel preparation techniques, drawbacks, and the scope of improvements. Being adaptable materials with a wide range of uses that take advantage of their special qualities, hydrogels are useful in many different industries, but especially in the fields of biomedicine, agriculture, electronics, water purification, and food packaging. Their capacity to imitate biological systems and react to changes in the environment creates new avenues for innovation and advancement. This review also discusses the working mechanisms of hydrogels in each of the aforementioned applications, shedding light on their interaction with biological, chemical, and physical environments to optimize their performance.

## 2. Preparation of Hydrogel Through Different Crosslinking

**Figure 1:**
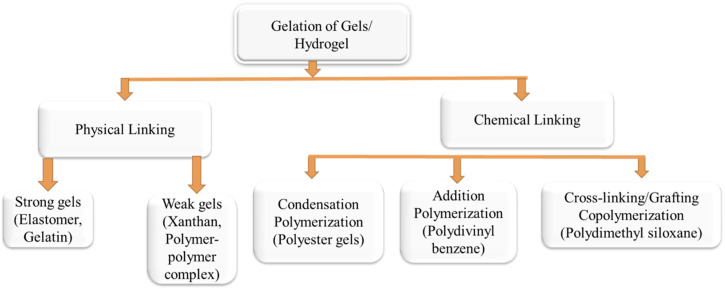
Different gelation takes place during hydrogel formation.

The macromolecules link together to form larger branched soluble polymers referred to as sol. Further linking of the sol results in bigger-sized and more branched polymers without solubility in water. This infinite and branched polymer is called a gel. Thus, the term “sol–gel transition” (also known as “gelation”) refers to the change from a system with finite branching polymers to one with infinite molecules, while the term “gel point” refers to the crucial point at which gel first manifests. There are several types of gelation taking place through physical or chemical cross-linking. The gelation that takes place in the hydrogel is presented in Figure 1 [20]. Based on the composition, properties, and application of hydrogels, specific methods of preparation are selected.

A phase transition process where molecules react or colloids collect, hydrogel production is a complicated process that involves both chemical kinetics and thermodynamic factors. Significant changes in material characteristics, including a fast increase in viscosity and the onset of elasticity, are caused by this process, which is characterized by the divergence of second and higher moments of molecular weight and cluster-size distributions. In view of kinetic aspects of gelation, reversible gelation, which includes cluster aggregation and fragmentation at the same time, is the fundamental process that controls the development of hydrogels. Population balance equations that monitor the time evolution of continuous cluster-size distributions describe these processes. Cluster size and molecular weight increase during aggregation, but clusters disintegrate with fragmentation. The overall gelation behavior is determined by the equilibrium between these two processes [21]. In view of the thermodynamic aspects of gelation, the process of gelation is characterized as a phase change. The equilibrium state and the existence of a non-gelling steady state are determined by thermodynamics, whereas the kinetic models explain the route to gelation. In reversible aggregation-fragmentation systems, the moment divergence, which indicates gelation, can happen even before equilibrium is completely reached. During gelation, it is essential to adhere to the fundamental thermodynamic principle of mass conservation. Throughout the procedure, the system’s overall mass is preserved. Under some circumstances, the absence of gelation is confirmed by the existence of a steady-state solution for the second moment. This suggests that under particular kinetic and thermodynamic circumstances, the system can achieve a stable, non-gelled state [22].

**Figure 2:**
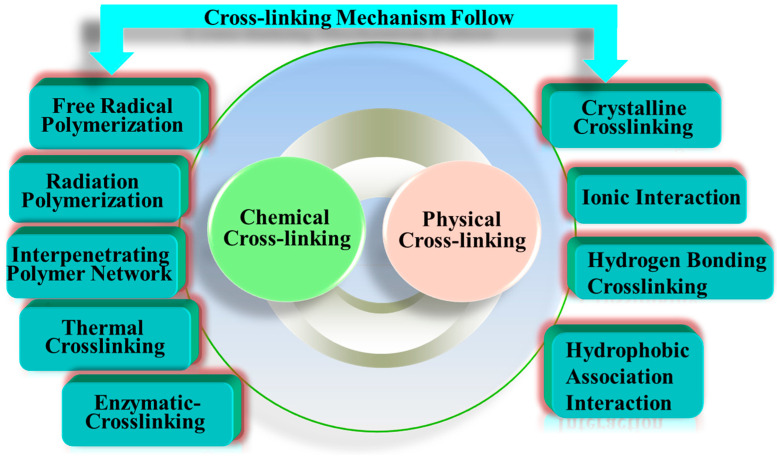
Different mechanisms followed by physical and chemical crosslinking.

One-step processes such as polymerization and parallel cross-linking of multifunctional monomers can be used to create hydrogels. This makes it possible to create networks of polymers with certain characteristics [23]. Reactive group-containing polymer molecules are created in many steps, and then the molecules are cross-linked with the help of appropriate agents [24]. There are three methods for creating hydrogels: solution, bulk, and radiation polymerization. The solvents like water, ethanol, dimethyl siloxane (DMSO), or combinations of these are used in the polymerization process. By swelling the hydrogels in water after the gel has formed, the solvent may be eliminated. A few cross-linking agents are added to the monomer mixture in the bulk polymerization process. Hydrogels with the required physical characteristics can be created by polymerization with radiation, UV light, or chemical catalysts. Ionizing radiation may be used to create free radicals that recombine as cross-link junctions, or it can be used to join polymer chains chemically to create hydrogels. Hydrogel cross-linking can also be facilitated by physical interactions such as entanglements, electrostatics, and crystallite formation. Although hydrophilic monomers are commonly utilized to create hydrogels, hydrophobic monomers can also be utilized to customize the gel’s characteristics for certain uses. The hydrogels’ mechanical strength and rate of degradation are influenced by the monomer selection. To improve their qualities and make them appropriate for the creation of hydrogels, natural polymers can be functionalized with radically polymerizable groups. To guarantee the purity and functionality of the finished product, hydrogels must be cleaned after preparation to get rid of contaminants like unreacted monomers, initiators, and cross-linkers [9]. Figure 2 shows two types of cross-linking methods follow different mechanisms [25], and Table 1 presents the advantages and disadvantages of different crosslinking methods.

Hydrogels that are physically cross-linked depend on non-covalent interactions to keep their structure. Because of worries about the toxicity of the cross-linkers employed in chemical procedures, this technique is seen as an alternative to chemically cross-linked hydrogels. Hydrogels that are physically cross-linked may be useful in situations where biocompatibility is essential [26,27]. Chemically cross-linked hydrogels are becoming increasingly popular because of their superior mechanical strength. Cross-links are included in these hydrogels to provide structural integrity while in use by preventing the breakdown of polymer chains [28].

**Table 1:** Advantages and disadvantages of different crosslinking.

Types of Crosslinking	Advantage	Disadvantage	References
Crystalline Crosslinking	Increased dimensional and thermal stability, improved chemical and solvent resistance, and superior mechanical toughness and fatigue life in some systems.	Decreased processability and recyclability, nonuniform characteristics, and residual stress.	[29]
Ionic Interaction Crosslinking	Self-healing and reversibility, mechanical characteristics that can be adjusted, responsiveness to stimuli, increased resilience thanks to sacrificial links.	Environmental sensitivity, restricted solvent stability or high temperatures, long-term flow and creep.	[29]
Hydrophobic Association Interaction Crosslinking	Toughness, energy dissipation, reversibility, and self-healing without the need for covalent chemistry.	Reduced permanent strength, difficulties with control and repeatability.	[30]
Interpenetrating Polymer Network Crosslinking	customizable mix of attributes, enhanced resistance to chemicals and solvents, Phase morphology control at tiny length scales.	complexity of synthesis and processing challenges, restricted phase separation and miscibility, decreased reprocessability and recyclability.	[31]
Free Radical and Radiation Crosslinking	No residues or chemical initiators, processing at low or room temperature, Quick, continuous processing.	High capital and infrastructure cost, Oxygen-induced deterioration, Limited penetration for thick or dense sections.	[32]
Thermal Crosslinking	Improved elasticity and mechanical strength, as well as customizable qualities.	stability problems and byproducts, restricted suitability for specific formulations, processing expense and complexity, longer times for curing.	[33]
Enzymatic Cross-Linking	High biocompatibility, selective cross-linking, and toxic reagent avoidance.	Slower reaction kinetics and limited mechanical strength.	[34]

### 2.1. Crystalline Crosslinking

**Scheme 1:**
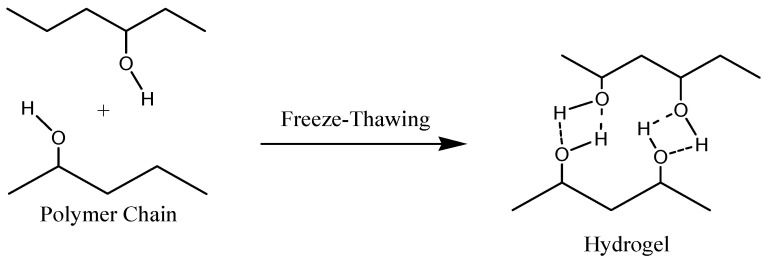
Crystalline crosslinking through freeze–thawing and hydrogen bonding.

In hydrogel synthesis, crystalline crosslinking is the process by which polymer chains crystallise and establish hydrogen bonds to form physical crosslinks, creating a three-dimensional network structure. This technique is frequently accomplished through the production of stereocomplexes or freeze–thaw cycles in homopolymeric systems. The hydrogel’s characteristics are greatly influenced by the kind and degree of crystallinity [35]. For example, when polyvinyl alcohol (PVA) is used to generate hydrogels, particularly during freeze–thaw cycles, crystallization is crucial for forming cross-links between PVA chains, as shown in Scheme 1. Regular pendant hydroxyl groups found in PVA have the ability to build crystallites by generating strong hydrogen bonds between chains. Hydroxyl groups are haphazardly placed on each side of both polymer chains in the two-molecule monoclinic unit cell that is characteristic of the crystals created with this technique [36].

Crystallisation within the polymer-rich microphages is made possible by the initial freezing of water and the ensuing expansion during the first freeze–thaw cycle, which raises the concentration of polymers in the unfrozen phase. The mechanical behaviour of these cryogels can be affected by processing factors such as the polymer concentration, the number of heat cycles, and the pace of thawing. For example, stronger cryogels might result from more heat cycles because they improve molecular chain aggregation and intermolecular hydrogen bonding [37]. Through the development of crystallites and inter-polymer interactions during a sequence of freeze–thaw cycles, a combination of polymers can generate connection sites in composite cryogels. In the hydrogel, these interactions operate as cross-linking sites. Nevertheless, the production of PVA crystallites may be adversely affected by the inclusion of another polymer, leading to less organised hydrogel formations. For instance, it has been demonstrated that raising the chitosan content in a chitosan-PVA mix decreases the hydrogel’s crystallinity [38].

### 2.2. Ionic Interaction Crosslinking

**Scheme 2:**
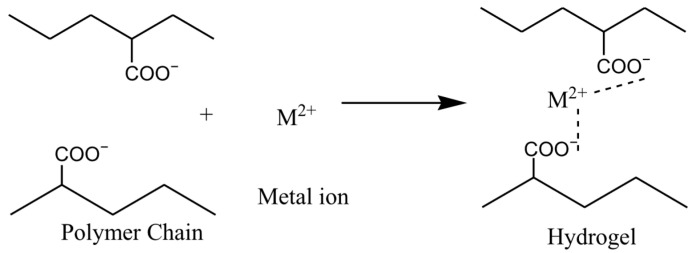
Formation of hydrogel through ionic interaction.

Ionic-based hydrogels use electrostatic forces between ions or polymers with opposing charges to build a crosslinked network. This technique is popular for producing hydrogels in moderate environments, such as room temperature and physiological pH, which makes it appropriate for use in biomedical applications [39]. For Example, bivalent ions and the carboxyl groups of alginate’s guluronic moieties combine to form an ionic gelation process, which is commonly used to create alginate-based hydrogels. A typical paradigm for this process is the ‘egg-box’ model [40]. A β-(1-4) glycosidic bond connects the monomers of β-D-mannuronic acid and α-L-guluronic acid to form alginate, a naturally occurring ionic polymer. The capacity of alginate to go through a sol/gel transition when bivalent cations like Mg^2+^, Ca^2+^, Sr^2+^, and Ba^2+^ are present is important for alginate gelation. According to this model, the bivalent metal ions—such as Ca^2+^, Sr^2+^, and Ba^2+^—function as crosslinking agents presented in Scheme 2. The hydrogel is a stable, three-dimensional network that is created when they attach to and connect neighboring guluronic blocks of the alginate chains. The term “egg-box” comes from the way this binding looks like eggs nestled in the dents of an egg carton [41]. Scheme 1 exhibits the ionic interaction between the carboxyl group of the alginate chain and a metal ion, forming the cross-linking to give a hydrogel.

However, this reaction mechanism is extremely sensitive to the kind and concentration of the bivalent ions, the concentration of alginate, the duration of the crosslinking process, and ambient conditions, all of which affect the hydrogel’s final characteristics [41].

### 2.3. Hydrophobic Association Interaction Crosslinking

**Scheme 3:**
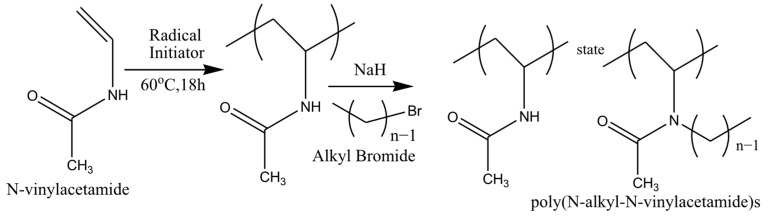
Synthesis of PNVA and poly(N-alkyl-N-vinylacetamide) hydrogel via hydrophobic association interaction [52].

**Figure 3:**
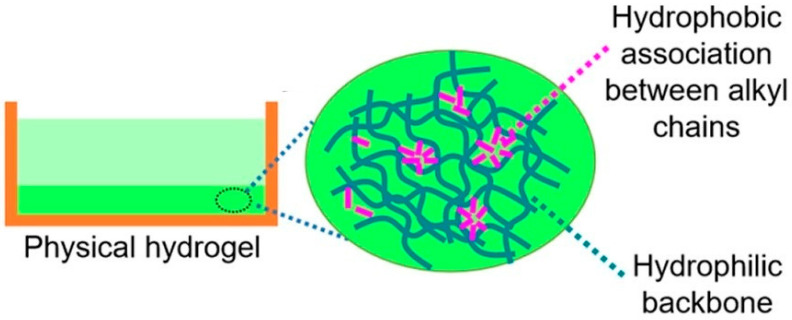
Hydrophobic association in the hydrogel [52].

When creating self-healing hydrogels, especially those made from amphiphilic polymers, hydrophobic interactions are essential. The main force behind this mechanism is the propensity of hydrophobic segments to reduce water interaction in an aqueous environment, which results in the creation of stable supramolecular structures. Reducing or removing the interaction between hydrophobic segments and water molecules requires the entropy-driven spontaneous process of hydrophobic associations. As a result, amphiphilic molecules in watery media self-assemble. Within the gel, there is a constant balance between free and linked hydrophobes. Reversible dissociation dissipates this energy when an external force is applied, preventing gel fracture and granting self-healing qualities. By varying the quantities of hydrophilic and hydrophobic segments, as well as the kind and size of the hydrophobic components, the mechanical strength of these hydrogels may be accurately regulated. Neutral O–H⋯O/N H-bond energies are typically ≈5–30 kJ·mol^−1^ per bond (strong H-bonds at the higher end; many biological H-bonds ~10–20 kJ·mol^−1^) [1]. Van der Waals force is weak per-atom contact, around 0.4–4 kJ·mol^−1^ per contact (extremely limited range; cumulative when numerous atoms interact) [42]. Water-mediated attractions between larger hydrophobic surfaces or aggregated domains can produce net free-energy gains of tens of kJ·mol^−1^ (i.e., comparable to or exceeding single H–bonds) because the driving force is the collective gain in solvent entropy and favorable solvation shell restructuring. However, for small nonpolar groups, the incremental transfer free energy per methylene is modest (order 0.6–1.0 kJ·mol^−1^ per –CH_2_–) [43,44]. Hydrophobicity is a solvent-mediated, frequently collective interaction rather than a single short-range pairwise potential. When several nonpolar groups cluster, the total free-energy gain scales supra-linearly because water releases ordered solvation shells and decreases unfavorable water–nonpolar contacts, resulting in multiple weak contacts adding up to a large net stabilization. Therefore, a clustered hydrophobic domain whose cumulative solvent-driven stabilisation approaches tens of kJ·mol−1 can easily match or outcompete a single H-bond (10–20 kJ·mol^−1^) [45,46]. Hydrophobic associations are stronger than hydrogen bonds and van der Waals interactions and may function across greater distances, although they are commonly regarded as weak. Increasing the medium ionic strength or temperature can also increase their strength [47]. Because ions screen electrostatic interactions and change water structure/solvation free energy, adding salt (monovalent salts up to ~0.5–1.0 M) often increases hydrophobic attraction (salting-out); the amount and sign depend on solute size/shape and ion identity (Hofmeister effects). Up to high salt (≈0.5–1 M), where effects are more noticeable, empirical and simulation investigations demonstrate quantifiable increases in hydrophobic association throughout common biological ionic strengths (tens of mM) [48]. Due to entropy-dominated solvation changes, many tests and simulations reveal an increase in hydrophobic attraction with rising temperature across the liquid water range (e.g., 20 → 60 °C); new investigations quantify an anomalous strengthening with T for nanoscale solutes. Modest heating (room temperature → 40–60 °C) frequently enhances hydrophobic driving force and can significantly alter mechanical behaviour for polymeric and protein-like materials [49]. Larger/extended hydrophobic surfaces and solutes that allow dewetting or collective solvent rearrangement exhibit more hydrophobic strengthening with salt or T; extremely tiny solutes (single methane) exhibit weaker, more immediate effects [50]. Making hydrophobized forms of polysaccharides like chitosan and dextran is another creative strategy. For instance, physically cross-linked hydrogels made from pullulan modified with cholesterol can be utilized to make drug-loading nanoparticles having a high-water content [51]. Using hydrophobic interactions in poly(N-alkyl-N-vinylacetamide) with alkyl chains as hydrophobic groups, Chunchen Zhang et al [52]. prepared the reversible hydrogel, as seen in Scheme 3. In order to introduce hydrophobic alkyl side chains onto the polymer backbone during the N-alkylation of poly (N vinylacetamide)s (PNVAs), alkyl bromides are a necessary reagent. When PNVA is N-alkylated, NaH acts as a powerful base that makes it easier for alkyl side chains to connect, resulting in modified polymers with hydrophobic properties [52]. In aqueous conditions, amphiphilic polymers—which have both hydrophilic and hydrophobic segments—self-assemble. Aggregates with a variety of shapes, including spherical or worm-like micelles, vesicles, or hydrogels, are produced as a result of this self-assembly. These structures are distinguished by hydrophilic chains encircling inner hydrophobic microdomains, as shown in Figure 3 [53]. 

Hydrophobic associations function as dynamic physical cross-links in a variety of self-healing hydrogels. After being exposed to external stress, the hydrogel can regain its original characteristics because of these reversible interactions. For example, hydrophobic segments can be used as cross-linking locations in the creation of amphiphilic polymers [54]. Hydrophobic interactions can be strengthened by the addition of surfactant micelles. Water-insoluble hydrophobic monomers can be dissolved by surfactant micelles, and the resultant polymer’s hydrophilic segments are joined by the cross-linking sites of the mixed micelles generated by hydrophobic monomers and surfactants. Micellar copolymerization has been utilized to create self-healing hydrogels [55]. Hydrophobic associations can also be strengthened by interactions between amphiphilic polymers and the hydrophobic cores of vesicle bilayers or nanoparticles. The amphiphilic polymer in these systems serves as a link between particles, creating supramolecular networks with self-healing and shear-thinning characteristics. Stiffer gels may be made using this technique [56].

### 2.4. Radiation-Induced Free Radical Crosslinking

**Scheme 4:**
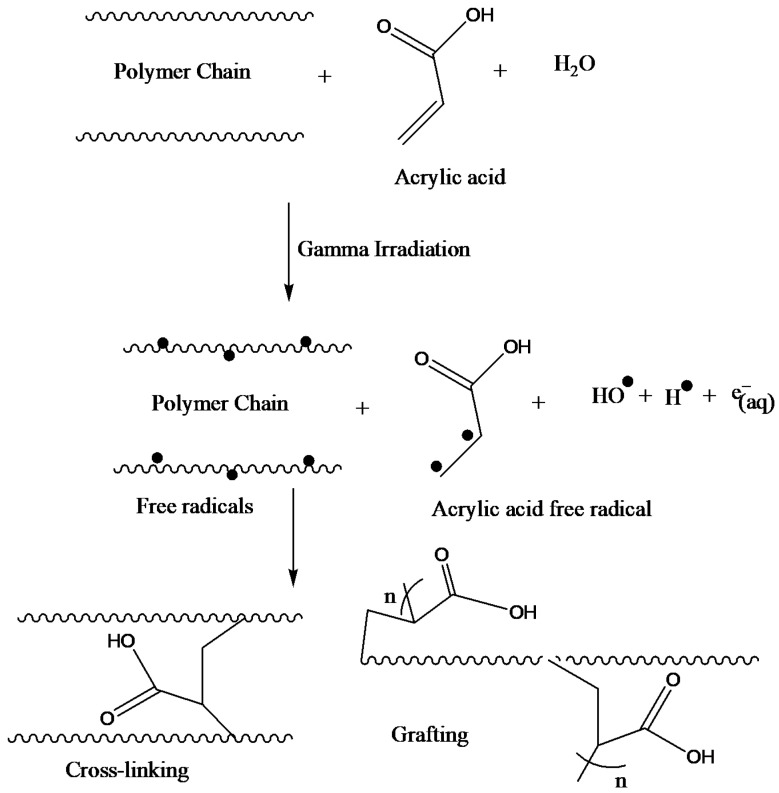
Radiation-induced crosslinking and grafting.

Crosslinking processes using radiation and free radicals can be used to create hydrogels. When polymer chains are exposed to free radicals, they join to produce crosslinks. This process is known as free radical crosslinking. Chemical initiators (such as ammonium persulfate and TEMED) or physical processes (such as UV light or high-energy radiation) can both produce free radicals. There are two forms of radiation (a) Ionizing (γ, e-beam, X-ray): photons or particles that have enough energy to ionize atoms or molecules (usually X-rays, high-energy electrons from e-beam accelerators, and γ-rays from 60Co). These are often classified as “ionizing radiation induced free radical polymerization” and directly generate primary radicals by bond scission and solvent/monomer radiolysis. Typically, they are given in kGy (1 kGy = 1000 Gy). Depending on the polymer/monomer, formulation, and presence of oxygen or sensitizers, crosslinking and polymerization typically take place over a period of 1–100 kGy [57]. For single-use medical devices, the standard industrial sterilization dosage is 25 kGy; higher doses can gradually cause chain scission, oxidation, discoloration, and embrittlement in polymers that are vulnerable. While γ gives a lower dosage rate (longer exposures), e-beam delivers a high dose rate (quick exposure durations). Radical recombination, oxygen transport, and therefore gelation versus degradation pathways are all impacted by dose rate; at very different dose rates, an identical total dosage might result in distinct consequences [58]. (b) Electromagnetic fields that do not directly ionize are known as non-ionizing radiation; UV (photons < ~10 eV) and microwaves are common examples in polymer processing. Through photoinitiators or direct absorption (photochemical initiation), UV starts free-radical polymerization. Microwaves can speed up thermal or thermally triggered radical processes by acting mainly through dielectric heating; any non-thermal microwave effects are still debatable and system-dependent [59]. Irradiance (mW·cm^−2^), exposure duration (s), and cumulative energy (J·cm^−2^ = irradiance × time) are important UV radiation metrics. The dosage needed to achieve vitrification or complete conversion in UV-cure formulations is determined by the photoinitiator, thickness, and absorptivity. Frequency (often 2.45 GHz in lab/industrial systems), power (W to kW scale for bulk materials), and exposure duration are important microwave radiation factors. The effects are mostly thermal (temperature vs. time being the main control). Although microwave-assisted polymerization windows vary greatly depending on the formulation, they are usually selected to quickly and consistently attain the desired reaction temperatures without protracted overheating that results in degradation [60]. Many crosslinked networks can be sterilized using ionizing radiation (25 kGy common target) if (i) the polymer is radiation-resistant (such as silicones or some polyolefins with stabilizers), (ii) the formulation contains antioxidants or UV stabilizers, and (iii) the network is sufficiently crosslinked and thermally stable to prevent incremental chain scission from compromising mechanical or functional properties. At sterilizing doses, polymers with large concentrations of labile bonds (ester, urethane, and certain elastomers) frequently exhibit property loss (embrittlement, discoloration); oxidative degradation is accelerated by oxygen and dosage rate [57]. Through direct chain scission or radiolysis of water, radiation crosslinking—often accomplished by irradiating polymer chains with gamma or electron beams—produces free radicals that, when they recombine, cause crosslinking [9]. This process entails the free radical polymerization of hydrophilic polymers or vinyl monomer mixes that have been derivatized by polymerizable groups. It makes it possible to create hydrogels using hydrophilic polymers that are natural, synthetic, and semi-synthetic. The development of designed structures and pure hydrogel preparation is possible using radiation polymerization [10]. Radical polymerisation can be accomplished in four different ways: emulsion, suspension, solution, and bulk polymerisation [61]. Radiation-induced free radical polymerization includes four main radiations, namely gamma, ultraviolet, microwave, and electron beam [62]. The radiation method offers advantages such as mild reaction conditions, negligible by-product formation, quick gelation, and the ability to achieve product purity and sterilization in a single step, making it beneficial for medical applications. Ionizing radiation: The most researched ionizing radiation techniques for hydrogel production are electron beams and gamma rays. Because of their great energy, these radiations can either directly or indirectly ionize the material they travel through. Additionally, synthesis and grafting may be accomplished using gamma radiation without the use of an initiator or crosslinker, producing extremely pure products [63]. Non-ionizing radiation, microwave has advantages, including increased yield and quicker reaction times. High response speeds, superior production yields in less time, fewer adverse effects, and enhanced product qualities are all results of microwave-assisted heating because of its uniform heating and rapid temperature increase [64]. Free radicals are released when covalent bonds break, which usually starts the radiation-induced ionization process. Due to its tremendous intensity, ionising radiation, such as electron beams and gamma rays, creates free radicals equally across the material they pass through in the first stage. For example, the radiolysis of water molecules in aqueous conditions results in radicals such as hydrogen (H•), hydroxyl (OH•), and hydrated electrons (e_aq_^−^). After attacking polymer chains, these radicals produce macroradicals [65]. Through targeted excitation, the microwave technique also breaks or cleaves bonds, producing free radicals on the polymer backbone. The ions and free radicals generated during the second stage of the process cause chemical interactions between molecules. The hydrogel is created when these macroradicals recombine on different strands to produce a crosslinked, three-dimensional structure [66]. For instance, when ethylene glycol (EG) and methacrylic acid (MAA) are grafted onto starch to create a hydrogel, the monomers and starch absorb radiation, get activated, and then separate to produce radicals that polymerise and crosslink [67]. During irradiation of an aqueous blend solution of raw materials, both the cross-linking and grafting may take place, resulting in the final hydrogel product, hydrogels shown in Scheme 4 [68].

**Figure 4:**
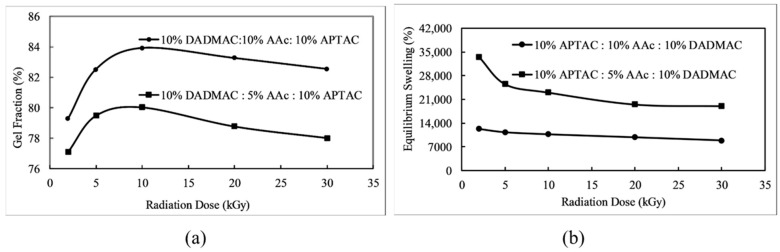
Effect of radiation dose on (**a**) gel fraction and (**b**) equilibrium swelling [70].

**Scheme 5:**
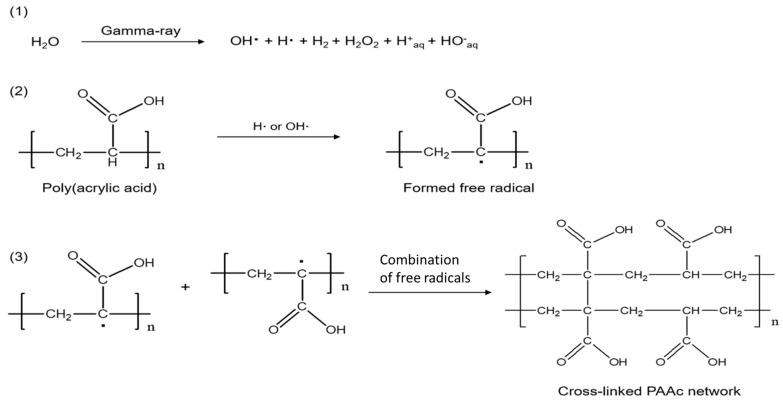
Gamma radiation-induced crosslinking of PAAc [69].

Jin-Oh Jeong et al. investigated the gamma radiation of 25 kGy induced polymerisation of polyacrylic acid for metronidazole (MD) drug delivery [69]. Scheme 5 presents the PAAc hydrogel formation mechanism upon gamma irradiation, where the free radicals are produced first, and then the combination of free radicals yields the product. The equilibrium swelling and the gel fraction are both strongly affected by the radiation exposure. Bhuiyan et al. reported the effects of radiation dose on the gel content and swelling of Diallyldimethylammonium Chloride-Acrylic Acid-(3-Acrylamidopropyl) Trimethylammonium Chloride (DADMAC-AAc-APTAC) superabsorbent Hydrogel, shown in Figure 4. Because not all of the raw material particles are activated for the process, there is less gel produced at lower radiation doses. The gel fraction first rises with increasing radiation, peaking at 10 kGy, before starting to decline. The radiation dosage and the equilibrium swelling percentage are negatively correlated. At lower radiation dosages, the greatest oedema is seen. For example, after a radiation dosage of 2 kGy, an equilibrium swelling of 33,483.48% was obtained with a composition ratio of 1:0.5:1 DADMAC:AAc:APTAC. The tendency of this swelling reduces up to 30 kG as the radiation exposure rises [70].

The number of free radicals generated initially determines whether radiation crosslinking can yield hydrogels. By altering the radiation dose, the degree of crosslinking—which has a direct impact on hydrogel swelling—can be changed [71]. In general, a higher dosage of irradiation results in a higher concentration of free radicals, which raises the degree of conversion and crosslinking. But going above a certain ideal dose might cause a compact structure with more crosslinking sites to develop, which can reduce performance and can even cause characteristics to deteriorate, especially in natural polymers. Network deterioration may result from higher dosages because intramolecular free radical recombination may become more common than intermolecular recombination [62].

### 2.5. Interpenetrating Polymer Network Crosslinking

**Figure 5:**
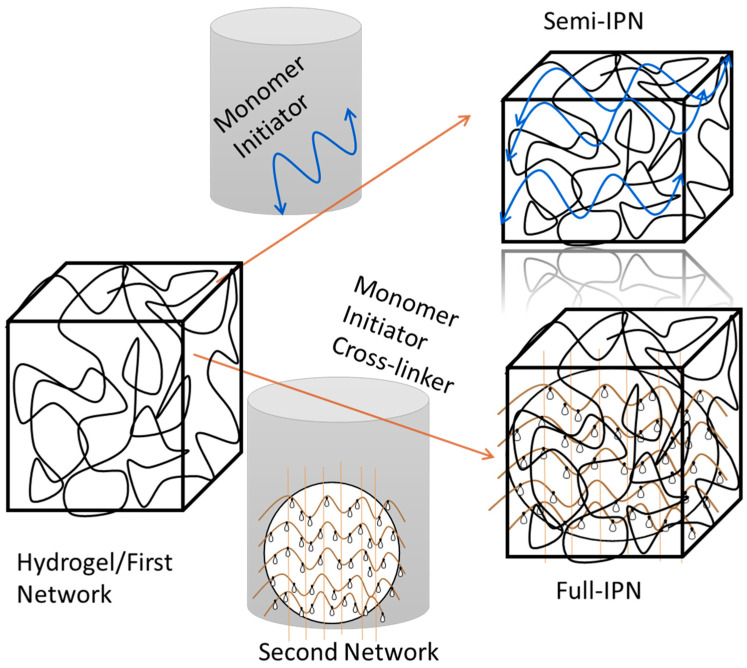
Schematic diagram of semi and full interpenetration hydrogel networks.

When a second hydrogel network is polymerised inside a prepolymerized hydrogel, an interpenetrating polymer network (IPN) is created. Usually, to do this, a prepolymerized hydrogel is submerged in a monomer and polymerisation initiator solution. As shown in Figure 5, IPNs can develop either with a cross-linker present to create a completely interpenetrating polymer network (full IPN) or without one to create a network of embedded linear polymers trapped inside the original hydrogel (semi-IPN) [72]. Thus, the double or triple network hydrogels are formed.

**Scheme 6:**
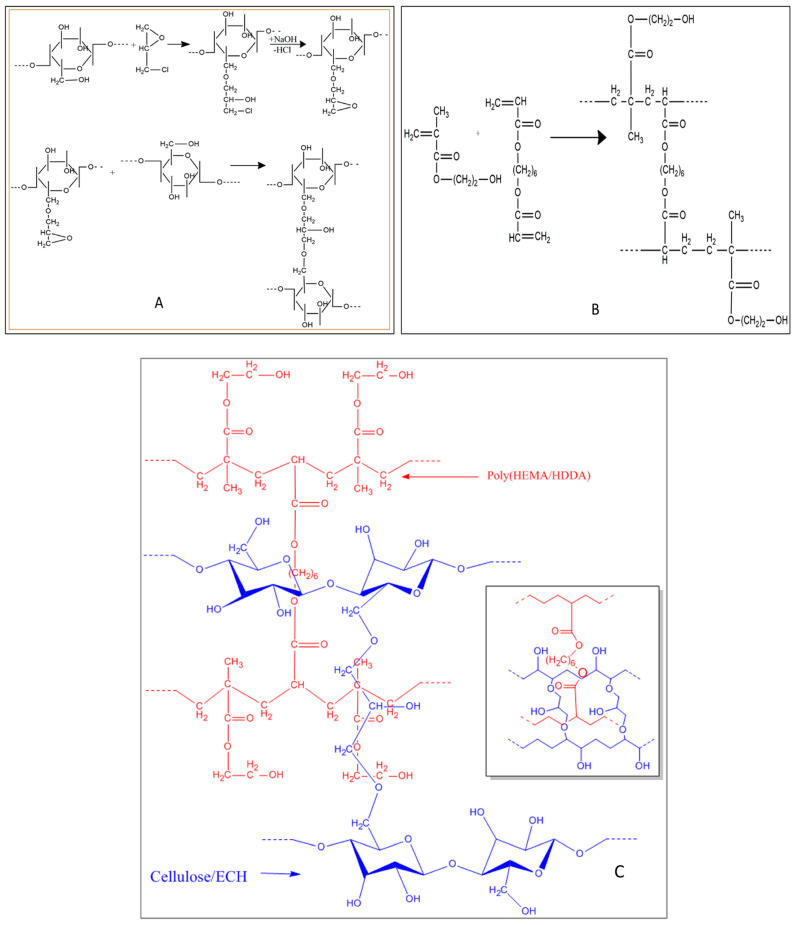
Schematic representation of (**A**) cellulose crosslinking by ECH (**B**) HEMA crosslinking by HDDA and (**C**) structure of an IPN based on cellulose and PHEMA, obtained by sequential crosslinking [73].

Meriem Mihoub et al. combined networks of cellulose and poly (hydroxyethyl methacrylate) (PHEMA) to create an interpenetrating polymeric network (IPN) by a sequenced process shown in Scheme 6 [73]. As crosslinkers, epichlorohydrin and 1,6-hexanediol diacrylate (HDDA) were used. Due to the hydrogen interaction between the cellulose and HEMA-OH groups, which improved the diffusion of HEMA monomer into the cellulose networks, the cellulose network showed significant swelling in a HEMA monomer medium. This IPN was used to remove dyes; when submerged in a solution of eosin Y and malachite green, it removed the former while leaving eosin Y behind, demonstrating its capacity for selective removal [73]. 

### 2.6. Thermal Crosslinking

**Scheme 7:**
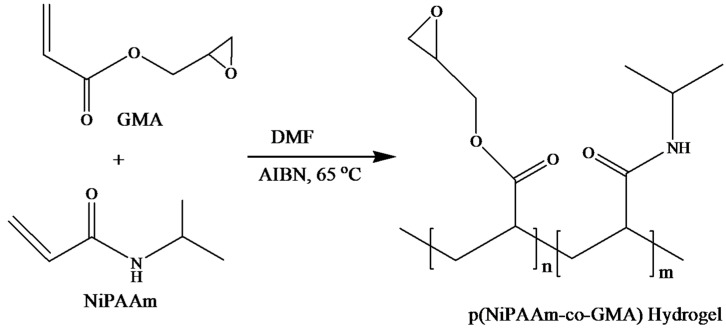
Thermally crosslinking between NiPAAm and GMA.

In order to create a three-dimensional network that can absorb and hold onto a significant quantity of water, thermal crosslinking is commonly used in hydrogel production to create chemical or physical crosslinks between polymer chains. This approach is flexible and may be accomplished by a number of processes, such as phase transitions, hydrogen bond reorganisation, free radical formation, or the breakdown of crosslinking agents [74]. N-Isopropylacrylamide (NiPAAm) -co- glycidyl methacrylate (GMA) can be thermally synthesized by free radical polymerization in DMF at 65 °C under a nitrogen atmosphere using 2,2′-azobis(2-methypropionitrile) (azobisisobutyronitrile, AIBN) as a free radical initiator (Scheme 7) [75].

In order to synthesize a stable chitosan/PEG-MA hydrogel network, Arman Jafari et al. explained a thermal crosslinking technique that does not require the use of traditional harmful crosslinkers or catalysts [76]. At a reaction temperature of 70 °C, they first created the crosslinking agent maleic anhydride-ended polyethylene glycol (PEG-MA) without the need for catalysts. Since this temperature is somewhat higher than the melting point of MA, MA will completely dissolve in PEG and produce a homogenous solution. PEG-MA and chitosan are combined with deionised water and agitated to form a clear solution in order to prepare a hydrogel. The hydrogel synthesis is then completed by pouring the solutions into silicone moulds and drying them in a vacuum oven at 100 °C. The interaction between the amine groups present on the chitosan backbone and the carboxylic acid groups at the end of the PEG-MA structure is what causes the heat crosslinking process. The crosslinks in the hydrogel network are amide bonds, which are created by this process. According to the study, amide linkages may be formed by interacting with the acid and amine groups at a temperature of 100 °C. Therefore, this thermal crosslinking technique offers a simple and biocompatible means of creating chitosan/PEG-MA hydrogels, which makes them appropriate for uses such as wound healing [76].

### 2.7. Enzymatic Cross-Linking

One type of chemical cross-linking is enzymatic cross-linking. It produces chemical links, especially covalent bonds, which are a feature of chemical cross-linking, even though it uses enzymes to catalyze the production of covalent connections between molecules. Hydrogel synthesis by enzymatic cross-linking is gentle and cell-friendly, which makes it appropriate for tissue engineering uses. The reactions usually take place in aqueous media, at moderate temperatures, and at neutral pH, avoiding the cytotoxicity linked to photo-initiators or organic solvents. Certain cross-linking processes are catalysed by different enzymes to create stable hydrogel networks [77]. A variety of enzymes can catalyse the creation of hydrogel networks by forming chemical connections between molecules. The most used enzymes are Glutamine Transaminase (TGase), Tyrosinase (TYR), Laccase, Horseradish Peroxidase (HRP), Glucose Oxidase (GOx). Phosphopantetheinyl transferase (PPTase), lysyl oxidase, plasma amine oxidase, Phosphatases, thermolysin, *β*-lactamase and phosphatase/kinase [78].

**Figure 6:**
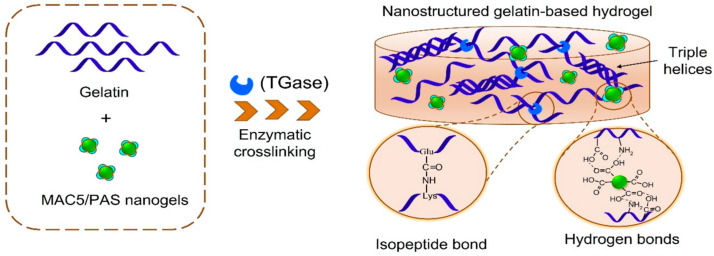
Schematic illustration of the fabrication of TGase-crosslinked gelatin hydrogel loaded with MAC5/PAS nanogels [84].

The enzyme glutamine transaminase (TGase) catalyses the deamination processes involving the γ-carboxamide group of glutamine residues and amine groups on proteins or free amino acids, as well as the creation of isopeptide bonds and amide bond transfers. TGase helps generate ε-(γ-glutamyl)-lysine isopeptide bonds intramolecularly or intermolecularly when lysine residues act as acceptors, changing the functional characteristics of the protein. It may be used, for instance, to create hydrogels from human-like collagen and bovine serum albumin and to cross-link hyaluronic acid, carboxylated chitosan, and human-like collagen to simulate extracellular matrices. Additionally, it has been used for flexible sensors using high acyl gellan gum and fish gelatin [79]. The copper-containing oxidoreductase enzyme tyrosinase (TYR) catalyzes the conversion of ortho-diphenols to o-quinones (diphenolase activity) and monophenols to o-quinones (monophenolase activity). With residues such as lysine, tyrosine, and cysteine in solutions, these reactive quinones easily generate covalent Y-K, Y-C, or Y-Y cross-links that aid in the formation of hydrogel structures. It has been utilised to create TA-conjugated HA by conjugating tyramine to hyaluronic acid. It then catalyzes the transformation of TA into o-quinones so that it may form covalent bonds with reactive amino acids in gelatin [80]. The multicopper blue oxidase protein family includes laccase, which catalyzes the oxidation of phenolic compounds such as aminophenols, polyphenols, diphenols, polyphenols, hydroxylated aryls, and aryldiamines. Because of its wide spectrum of substrates and excellent catalytic efficiency, this enzyme may be used in a variety of reactions, including polymerisation procedures. In order to increase the effectiveness of cross-linking, it can bind small-molecule phenolic mediators and start non-enzymatic cross-linking by forming radicals. Examples include its application in the formation of hydrogels from silk-elastin-like protein (SELP) by establishing dityrosine linkages and in the manufacture of hydrogels from precursor pectin by cross-linking ferulic acid molecules [81]. Horseradish Peroxidase (HRP) is a heme-containing biocatalyst that facilitates the cross-linking of phenols, anilines, and their derivatives by catalysing oxidative processes when hydrogen peroxide (H_2_O_2_) is present. Hydrogels made from polymers with phenolic hydroxyl groups have been prepared by HRP-catalyzed processes; active C residues oxidise glucose to produce H_2_O_2_. Dopamine-modified carboxymethyl cellulose and dopamine-modified hyaluronic acid have been used to create CMC-DA hydrogels and hydrogels with quaternized chitosan mixes, respectively [82]. Specifically, in aerobic circumstances, β-D-glucose is oxidised by glucose oxidase (GOx) to produce gluconic acid and H_2_O_2_. Cross-linking substrates can be immediately oxidised by the H_2_O_2_ generated by GOx. Additionally, it is used with HRP to facilitate the cross-linking of polymers that contain phenolic hydroxyl groups, including HA derivatives, gelatin, poly (ethylene glycol)-tyramine, and others [83]. Alina Gabriela Rusu et al. prepared enzymatically crosslinked gelatin hydrogels loaded with amoxicillin nanogels (using transglutaminase, TGase) for possible use as wound dressings [84]. By combining gelatin, maleoyl-chitosan (MAC5)/PAS nanogels, and TGase solution (at doses of 2 U/g and 5 U/g), as seen in Figure 6, enzymatically crosslinked nanostructured hydrogels were created for this investigation.

## 3. Application of in Different Fields

Hydrogels are extensively used in the biomedical field (Drug Delivery Systems, Tissue Engineering, Artificial Muscles) due to their biocompatibility and ability to mimic biological tissues [18]. In the food industry, hydrogels are gaining attention for their Antibacterial Properties and Moisture and Gas Barriers [85]. Other significant applications of hydrogels are in environmental remediation, Robotics and Soft Robotics, Medical Implants, and Diagnostic Devices. A few applications of hydrogels with functional mechanisms are discussed here. The separation mechanisms of hydrogels in wastewater purification are multifaceted, involving size exclusion, charge interactions, electrostatic forces, functional group interactions, and the influence of environmental conditions. Together, these processes increase the effectiveness of pollutant removal, which makes hydrogels a viable wastewater treatment option [86]. Functional mechanisms of hydrogels in different sectors are discussed explicitly here.

### 3.1. Application in Adsorption

**Figure 7:**
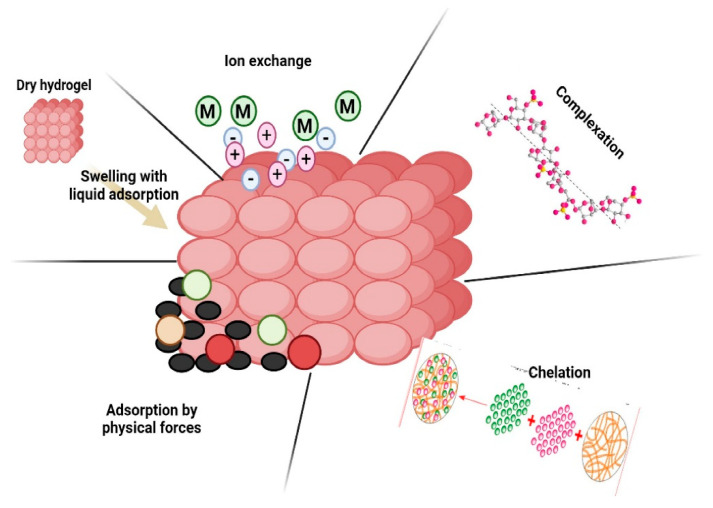
Different mechanisms of hydrogel adapt for contaminant removal [28].

Using techniques including ion exchange, complexation, chelation, and adsorption by physical forces, as illustrated in Figure 7, hydrogels aid in the removal of pollutants, dependent on their characteristics and ambient variables like pH and ionic strength [87]. By altering chemical interactions, functional groups in hydrogels have an impact on their ability to absorb contaminants. For instance, the complexity of the nitrogen atoms in the hydrogel structure is primarily responsible for the adsorption of lead ions [88]. After adsorbing pollutants, the hydrogel’s crystalline structure changes, suggesting that amorphous areas are important to this process [9]. The pH of the solution affects cationic hydrogels’ ability to readily absorb water and permit the entry of negatively charged metal ions [89]. Therefore, hydrogels’ structure and interactions with contaminants are related to how well they remove pollutants. Table 2 lists efficient hydrogels applied in different dye and heavy metal adsorptions.

Wastewater treatment involves the removal of dyes, heavy metals, micropollutants, and pharmaceutical contaminants [90]. Common Heavy metal ions, including uranium, arsenic, copper, cadmium, chromium, lead, and mercury, are harmful to health due to their toxicity and ability to accumulate in living organisms [91]. Heavy metals may be visually detected by functionalized hydrogels, which expand their use in monitoring. Metal–organic frameworks (MOFs) and other nanomaterials can be added to hydrogels to improve their adsorption capabilities. The goal of ongoing research is to enhance the effectiveness and use of hydrogels in the removal of heavy metals [92].

Suyash Pandey et al. reported the novel sodium alginate-derived maleic acid N,N methylenebisacrylamide-acrylic acid (SMNA) hydrogels, which were tested for removing heavy metals from wastewater and showed the best performance, with high swelling ratios and removal rates of 96.38% for copper and 94% for cobalt ions, along with strong adsorption capacities [93].

**Figure 8:**
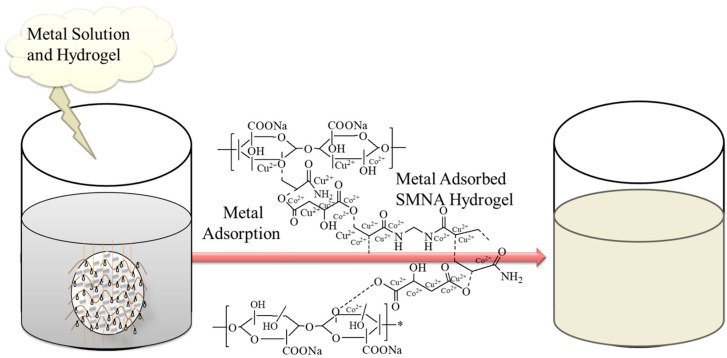
Adsorption of Cu^2+^ and Co^2+^ by SMNA hydrogel.

Figure 8 shows a thorough procedure for the adsorption mechanism of Cu^2+^ and Co^2+^ ions onto the SMNA hydrogel. The cross-linked network of the SMNA hydrogel contains a number of functional groups, such as -OH and -COO^−^. Through a combination of chemical and physical interactions, these functional groups are essential for collecting Cu^2+^ and Co^2+^ ions. The hydrogel effectively forms a link with the metal ions in the polluted water by using these functional groups. Strong Cu^2+^ and Co^2+^ ion adsorption is made possible by the hydrogel’s chemical structure, indicating how well the SMNA hydrogel removes heavy metal pollutants. Therefore, effective removal of the metal ions from polluted water is made possible by the adsorption mechanism of Cu^2+^ and Co^2+^ ions onto the SMNA hydrogel, which includes interactions through functional groups in the hydrogel’s structure [93]. Dyes are a major source of pollution used in many industries, including cosmetics, textiles, and paints. Anionic, cationic, and non-ionic dyes are the three categories into which they may be divided. The kind of dye, the water environment, and the adsorbent’s modification all affect how well the dye is removed. Treating complicated water pollution requires the development of multifunctional adsorbents that are capable of removing both cationic and anionic dyes [94,95,96]. Certain hydrogels may be made to function well with both kinds of dyes and can be reused repeatedly without losing their efficacy. By dissolving dyes when exposed to light, photocatalytic elements such as TiO_2_ and magnetic particles may be added to hydrogels to enhance dye removal. These materials’ adsorption capacity is limited, though, and careful design is required to preserve their efficacy [97,98].

Hussain et al. provided insightful information about the creation of SA/SiO_2_/ZnO hydrogel nanocomposites for efficient removal of malachite green dye [99]. Notable advantages include the thorough experimental design and in-depth adsorption mechanism investigation. For these promising adsorbents to be used in the real world and commercialized, it will be essential to solve the practical issues of pH sensitivity, possible ZnO-NP leaching, and scalability. For substantial ecological cleanups, future research should concentrate on improving composition, expanding the applicability of pollutants, and guaranteeing environmental safety and economic viability [99]. Heavy metals and dyes may be selectively removed by Guipi Residue/Chitosan/Acrylamide/Acrylic acid hydrogels; however, the adsorption efficiency is insufficient [100]. 

**Table 2:** Application of hydrogel in dye and metal adsorption.

Hydrogel Composition	Properties	Field of Application	Function	Reference
SA/SiO_2_/ZnO-NPs	The malachite green dye adsorption in hydrogel is endothermic and spontaneous	Dye adsorption	The maximum adsorption capacity of 322.58 mg/g	[99]
Guipi Residue/chitosan/ Acrylamide/Acrylic acid	Selective removal of heavy metals and dyes	Selective Removal of Heavy Metals and Dyes	Adsorption capabilities 314.6 mg/g for Pb^2+^, 289.1 mg/g for Cd^2+^, 106.4 mg/g RhB and 94.8 mg/g for MO dyes	[100]
Iron(III) chloride hexahydrate/sodium alginate	Rapidly remove toxic contaminants (dyes) from aquatic food matrices	Dye adsorption	The maximum adsorption capacity is 315 mg/g	[101]
Nanocellulose/(2-acrylamido-2-methylpropane sulphonic acid)	Adsorb both the cationic and anionic dyes	Dye adsorption	The maximum adsorption capacity is 357.143 mg/g	[96]
Acrylic acid/Acrylamide (Dimethylamino) ethyl methacrylate/Maleic acid (PAA/PAM/[DMAEMA]MA)	Hydrogel can adsorb both anionic and cationic dyes.	Dye adsorption	Adsorption capacity for methylene blue (MB), rhodamine B (RHB), Congo red (CR), eosin B (EB) were 489.1, 463.2, 465.5 and 462 mg/g (amount of dye adsorbed per gram of hydrogel),	[102]
H_2_TiO_3_/Poly (Acrylamide)	Exhibits excellent mechanical properties, adsorption performance, and recyclability	Metal Adsorption	Li^+^ adsorption capacity of 25.4 mg/g	[103]
Sodium alginate/acrylamide/maleic acid	Exhibits higher swelling and a spontaneous adsorption.	Metal Adsorption	Maximum adsorption capacities of 568.54 mg/g for Cu^2+^ and 342.42 mg/g for Co^2+^ ions	[93]
Cu-based Sodium alginate/chitosan/cellulose nanofibril composite	Shows spontaneous and endothermic adsorption	Metal adsorption	The maximum adsorption capacity could reach 531.38 mg/g	[92]
Acorus calamus leaf lignin/acrylamide(ACL-PAM)	Hydrogel exhibit chemisorption as the dominant mechanism	Metal adsorption	The maximum adsorption capacities were 340.45 mg/g for Pb (II) and 287.38 mg/g for Cd(II)	[104]
Bacterial cellulose based thermosensitive amidoximated (BCOU).	Hydrogel is thermosensitive	Adsorption: Uranium	Adsorption capacity is 485.89 mg/g after five cycles of adsorption and desorption.	[105]

While hydrogels, especially the PAA/PAM/[DMAEMA]MA hydrogel, have several benefits for dye adsorption, they also have certain disadvantages. With capacities of up to 489.1 mg/g for methylene blue (MB), rhodamine B (RHB), Congo red (CR), and eosin B (EB), the synthesized hydrogel exhibits a high adsorption capacity for a variety of dyes. This demonstrates how well it works to extract substantial amounts of dye from water. The effective contact areas between the hydrogel and the dyes are increased by the porous network structure of the hydrogel, which promotes dye adsorption. This hydrogel can concurrently adsorb both cationic and anionic dyes, in contrast to many other previously described hydrogel adsorbents that are single selective for either cations or anions. This increases its suitability for treating wastewater with mixed dyes. The hydrogel’s adsorption capability tends to saturate after around half an hour, indicating that it can absorb dye ions quite quickly. Practical applications benefit from its quick adsorption. The preparation technique is quite straightforward because hydrogel is created in a single pot utilizing ionic liquids as crosslinking agents. Dye may be efficiently adsorbed by the hydrogel adsorbent at room temperature and pH values close to neutral, which are frequently preferred for real-world uses. pH has a major impact on the hydrogel’s adsorption behavior. When compared to neutral circumstances, its adsorption capacity (Qe) is significantly reduced in very acidic (pH = 3) and strongly alkaline (pH = 11) environments. This restriction raises the possibility that, in some wastewater streams, pH modification may be required for best results. For some dyes, a temperature rise of up to 30 °C can enhance adsorption; however, temperatures over this point will disrupt the hydrogel’s three-dimensional network structure and impair the chelating action, which will decrease its adsorption capacity. This suggests a range of temperatures for best results. The development of highly effective inorganic dye adsorbents is often difficult, as seen by the limited adsorption capacity of previous adsorbents such as graphene oxide modified zeolite, despite the hydrogel’s generally high adsorption capacity. Even if this particular hydrogel seeks to address this, it draws attention to a typical obstacle in the industry [102]. The H_2_TiO_3_@PAM hydrogel solves important issues with conventional powdered ion sieves and provides a number of benefits for lithium ion (Li^+^) adsorption. Nonetheless, there are several disadvantages associated with preserving ideal material qualities. Poor fluidity, permeability, and recovery problems that are typical of powdered lithium-ion sieves are successfully addressed by the hydrogel form. Li^+^ desorption is facilitated, and the overall adsorption efficiency is improved by the hydrophilic groups in polyacrylamide, particularly the polar amide groups (-CONH_2_). This is accomplished by increasing the rate at which lithium ions are captured, decreasing resistance, and creating hydrogen bonds with water molecules. An adsorption performance of 32.8 mg/g was attained with the ideal HTO@PAM-4 ratio. The hydrogel’s strong tensile strength (0.19 MPa for HTO@PAM-4) indicates its resilience and capacity to preserve structural integrity, both of which are essential for real-world uses. With a water contact angle of 8.9°, the material exhibits strong hydrophilicity, which makes it easier for the active sites and Li^+^ ions in solution to interact. After six cycles, the HTO@PAM-4 hydrogel retains 77% of its maximal adsorption capability (25.4 mg/g), indicating high recyclability. It is appropriate for complicated brines due to its strong selectivity for Li^+^ over other coexisting metal ions such as Na^+^, K^+^, Mg^2+^, and Ca^2+^. It was constructed using an in situ polymerization process that is inexpensive, easy to use, and requires fewer stages. The material’s large specific surface area and continuous porous structure are advantageous for Li^+^ adsorption and transport. A uniform hydrogel cannot develop if the fraction of HTO particles is too large (x > 4), which results in a notable reduction in mechanical characteristics. Overuse of the cross-linking agent (MBA) can make a material overly brittle and hard, which lowers its tensile strength and deformation capacity before breaking [103]. A potential adsorbent for the removal of heavy metals is the ACL-PAM hydrogel, which is made from acrylamide and lignin from Acorus calamus leaves. Although there are certain drawbacks, its design includes several beneficial elements. Excellent adsorption capabilities for heavy metal ions, particularly Pb(II) and Cd(II), are demonstrated by the hydrogel. According to the Langmuir model, it reached maximum adsorption capacities of 287.38 mg·g^−1^ for Cd (II) and 340.45 mg·g^−1^ for Pb(II). This large capacity is essential to the effective removal of pollutants. ACL-PAM and other hydrogels have a high specific surface area (SSA) and a highly porous three-dimensional (3D) structure. This design gives metal ions several accessible locations, which improves mass transfer and adsorption efficiency. In particular, the produced ACL-PAM hydrogel displays a dense, asymmetrical, and intricately linked network of pores. Hydroxyl (-OH), amino (-NH_2_), carboxyl, and carbonyl (C=O) groups are among the several surface functional groups found in the hydrogel. Effective removal is made possible by these groups’ strong complexation and electrostatic interactions with metal ions. After five cycles, the hydrogel demonstrated good regeneration ability, keeping a significant amount of its adsorption capability. For example, after five cycles, removal efficiencies for Pb (II) and Cd (II) were 84.1% and 70.7%, respectively, suggesting favorable regeneration capability. For wastewater treatment to be sustainable, this reusability is essential. Strong mechanical strength and good heat stability are features of the ACL-PAM hydrogel. Its strong three-dimensional network structure, generated by excellent crosslinking, helps to its stability and inhibits dissolution in aqueous solutions. A simple and environmentally friendly method is used to create a hydrogel from biomass resources (Acorus calamus leaf lignin). Because of this, it is an ecologically safe and sustainable adsorbent. By increasing its internal pore volume and facilitating the passage of metal ions inside the gel matrix, the hydrogel’s exceptional swelling capacity improves adsorption efficacy. Although hydrogels have benefits over some natural biomass materials, these materials’ practical uses are restricted by problems such as pore blockage, quick site saturation, and limited ion diffusion. Even if the porous nature of the ACL-PAM hydrogel attempts to counteract this, binding site saturation still happens, which causes the adsorption capacity to plateau as the concentration of pollutants rises. Excess hydrogel particles tend to combine and form bigger clusters at increasing doses. This aggregation can lower the adsorption capacity per unit mass of hydrogel by reducing the effective specific surface area and impeding the interaction between metal ions and active functional groups. The hydrogel shows strong regeneration, but after several cycles, its removal effectiveness drops somewhat (for example, after five cycles, it went from 98.5% to 84.1% for Pb (II) and from 98.0% to 70.7% for Cd (II). This decrease is ascribed to partial metal ion desorption, which might obstruct some active adsorption sites. pH has a significant impact on adsorption performance; for Pb (II) and Cd (II), the best efficiency occurs in particular pH ranges (pH 4.0–5.5 for Pb (II) and 4.0–8.0 for Cd (II)). The hydrogel surface becomes strongly protonated in extremely acidic circumstances (pH = 2.0), which results in substantial electrostatic repulsion with positively charged metal ions and drastically lowers adsorption effectiveness [104]. Aside from several benefits for uranium adsorption, the thermosensitive BCOU hydrogel, which is made from amidoximated bacterial cellulose, has several intrinsic disadvantages pertaining to its use and characteristics. Up to 580.89 ± 2.67 mg/g of uranyl ions (UO_2_^2+^) may be adsorbed by BCOU under ideal circumstances (pH 4.5, 55 °C, 90 min contact duration, and 1.0 mg/mL UO_2_^2+^ concentration). Compared to other cellulose derivatives and their non-imprinted equivalent (NBCOU), this capacity is noticeably higher. After five cycles of adsorption and desorption at 55 °C, the hydrogel retains 83.65% of its initial adsorption capacity (485.89 mg/g), demonstrating remarkable reusability. Because it lowers operating costs and material waste, this is essential for real-world applications. Since BCOU is a thermosensitive hydrogel, temperature affects how it behaves. At higher temperatures (above its volume phase change temperature, or VPTT), it experiences a reversible phase transition and contracts in volume. Because the hydrogel expands when cooled, it facilitates the release of deposited UO_2_^2+^ ions, which is advantageous for both adsorption (because higher temperatures might speed up endothermic adsorption) and regeneration. Compared to acid-based techniques, the effective desorption of uranium ions with less environmental impact is made possible using Na_2_CO_3_/H_2_O_2_ as an eluent. In comparison to non-imprinted materials, BCOU exhibits greater adsorption selectivity due to the creation of specialized recognition sites for UO_2_^2+^ ions by the ion-imprinting process utilized in its manufacture. This is essential for uranium extraction from complicated mixes with competing metal ions, such as saltwater. As the foundation material, bacterial cellulose (BC) has benefits over microcrystalline cellulose (MCC) because of its distinct three-dimensional fibrous network, which improves interaction with reactants and affords more active sites for adsorption. Additionally, BC is made via industrial fermentation, which is less impacted by environmental conditions and does not require land resources. Although thermosensitivity has its benefits, severe hydrogel shrinkage at temperatures higher than its final phase transition might limit the amount of space available for UO_2_^2+^ penetration, which lowers the adsorption capacity. This suggests that for the best results, careful temperature control is required. Reusability is good; however, with time, adsorption capacity and desorption rates may be marginally decreased due to structural changes in the gel network caused by repeated adsorption–desorption cycles, such as molecular chain breaking and imprinting site loss. The adsorption capacity may be somewhat lower in solutions with additional metal ions than in solutions containing solely UO_2_^2+^, despite its greater selectivity because of the ion imprinting. This is explained by other metals competing with it for surface adsorption sites via electrostatic or coordination interactions. Higher temperatures are often advantageous for uranium adsorption due to the endothermic nature of BCOU’s uranium adsorption. Although this can be controlled, in large-scale applications, energy input could be necessary to maintain ideal temperature conditions [105].

### 3.2. Application in Agriculture

**Figure 9:**
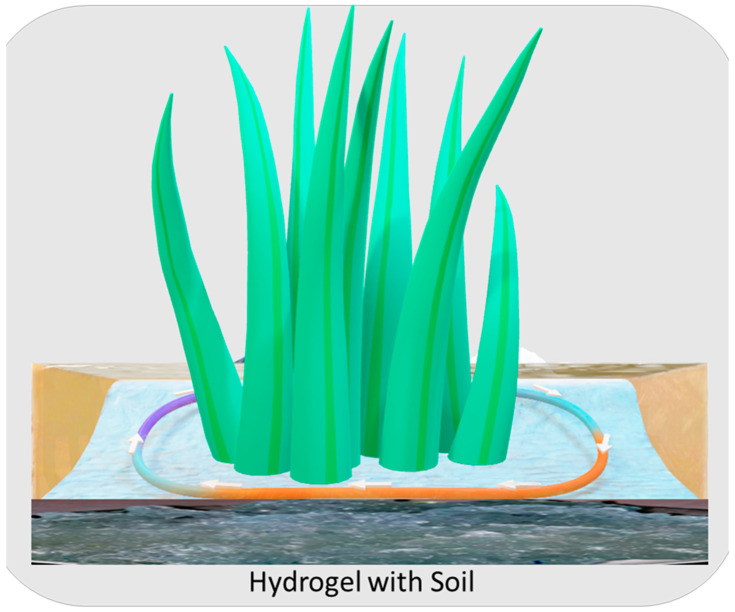
Slow supply of water and nutrients from hydrogel to plant.

The main source of food production, agriculture, guarantees that people have access to vital nutrients. Global food security is directly impacted, particularly considering climate change, which modifies precipitation patterns and intensifies extreme weather events that jeopardize agricultural output. National economies greatly benefit from the agriculture sector. Millions of people throughout the world can find work thanks to it, especially in underdeveloped nations where agriculture frequently serves as the foundation of the economy [106]. By optimizing resource utilization, precision agriculture and other cutting-edge techniques increase the effectiveness of water and fertilizer application. This lessens the impact on the environment by increasing production and decreasing the need for artificial input. To repair damaged land and guarantee long-term agricultural resilience, a move towards regenerative farming techniques is required. For sustainable food systems, this entails boosting carbon sequestration and enhancing soil health [107]. Currently, direct watering techniques are used in most agricultural systems, which can result in substantial water loss. Crop water shortages might result from this inefficiency, particularly in areas with already scarce water supplies. Although targeted sprinkling systems can reduce water loss, their equipment costs are high. This expensive obstacle may keep many farmers from implementing more effective irrigation techniques, which would exacerbate problems with the water supply [107]. Concern over soil desertification, which impacts agricultural areas’ ability to retain water, is rising. The soil’s capacity to retain water decreases with deteriorating quality, which makes crop production and sustainability even more challenging. Sustainable farming methods that enhance water management are desperately needed to address these issues. This entails investigating substitutes for conventional watering techniques and improving soil characteristics to better hold onto moisture [108]. Super absorbent polymers (SAPs) have the potential to significantly increase soil water retention. Farmers may improve soil moisture levels and lower the frequency and amount of irrigation required by implementing SAPs, which have a significant capacity to absorb and hold water [109,110]. Hydrogels, a kind of SAP, can be used as soil supplements and water suppliers to greatly increase plant survival and growth, as shown in Figure 9. In dry and semi-arid regions, where water management is essential for successful farming, they aid in increasing the soil’s water content and retention [108,111].

**Figure 10:**
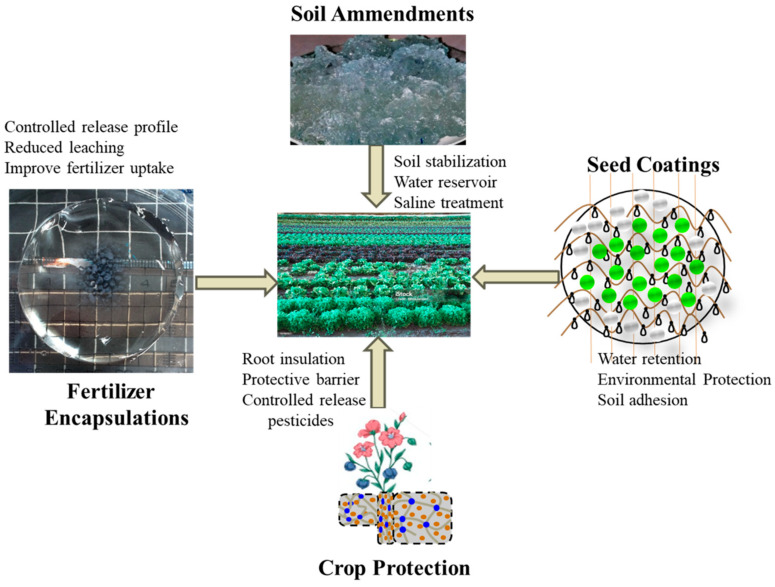
Different way of Application of hydrogel in agriculture.

Depending on environmental factors including moisture and temperature, fertilizer release rates might vary greatly. For example, studies show that the impact of temperature on fertilizer release rate is more consistent with Nutricote fertilizers than with others, such as Polyon. Crop development and output may be impacted by this fluctuation if it results in uneven nutrient availability. For different controlled-release fertilizers (CRFs), the rate of fertilizer release can be postponed by 20 to 40 days. Particularly during critical growth phases, this delay may impede adequate nutrient availability, which is essential for optimal plant growth [112]. Temperature variations have a significant impact on fertilizer release efficiency. Lower temperatures (5 °C to 15 °C) result in minimal fertilizer release, whereas intermediate temperatures (20 °C to 30 °C) show steady-state release. In colder regions or seasons, this temperature dependency may reduce fertilizer efficacy [113]. Concern over fertilizer use’s long-term sustainability is rising. One possible remedy is the creation of smart biodegradable hydrogels (SBH) for fertilizer coating; however, there are still issues with making sure that these substances can efficiently satisfy plant nutrient requirements while being ecologically benign [114]. Enhancing fertilizer effectiveness requires the capacity to regulate fertilizer delivery through a variety of variables, including pH, temperature, and biological activity. It is still difficult to achieve this control in real-world applications, though [115]. Application of hydrogels for supplying water and fertilizer into agriculture can be classified into four categories presented in Figure 10. Table 3 presents effective hydrogels for adsorption of heavy metals and dyes from wastewater and other sources.

When used in agriculture, hydrogel, especially those made of natural biopolymers, offer a few advantages as well as certain drawbacks. Hydrogels reduce water loss during infiltration processes by greatly increasing the soil’s maximum water holding capacity (MWHC). As a result, the soil’s accessible water content rises, better satisfying plants’ water needs. For instance, depending on the concentration, the L/KJ/SA hydrogel raised MWHC by 2.98% to 8.96%. Essential nutrients such as nitrate nitrogen, ammonium nitrogen, total phosphorus, and accessible potassium might be less likely to leak from the soil when hydrogels are used. For example, the L/KJ/SA hydrogel decreased the leaching of these nutrients by 14.60% to 38.83%, in different proportions. This is because water and soil nutrients may both enter the hydrogel’s molecular network, minimizing fertilizer penetration and leaching. Plant growth can be extended by the L/KJ/SA hydrogel, especially when drought stress is present. They assist plants in dealing with drought by enhancing their photosynthetic efficiency and influencing intracellular regulators like proline and decreasing sugar. When compared to control groups, tobacco plants treated with L/KJ/SA hydrogels exhibited noticeably better photosynthetic rates. Bio-based hydrogels are thought to be more ecologically benign and more degradable in soil than many synthetic hydrogels derived from petroleum products (such as polyacrylamide and acrylic acid). After 120 days of being buried in soil, the L/KJ/SA hydrogel showed good degradability, breaking down by 20% while still supplying enough water for plant development cycles. Because many commercial hydrogels contain petroleum-derived ingredients, they may have harmful effects on the respiratory and neurological systems of humans or animals. The L/KJ/SA hydrogel and other bio-based hydrogels are made to be non-toxic. The saturated hydraulic conductivity of soil can be reduced by adding hydrogels. This is due to hydrogels’ fluid colloid properties, which can inhibit water penetration, increase friction between soil particles, and clog soil pores, all of which reduce water loss during the infiltration process. Certain biogels, especially those composed mostly of polysaccharides, have a high rate of degradation, which may not be ideal for uses where plants need a longer-term water supply. One research study, for example, discovered that after only two weeks, 43.3% of the polysaccharide had degraded. Pure hydrogels’ useful duration in soil may be limited by their weak mechanical qualities and rapid breakdown. Even though hydrogels usually increase the availability of water, some—such as commercial acrylamide hydrogels—may hold water in a way that is mainly inaccessible to plants, particularly when water suction is strong [116]. Compared to P(AA) and P(AA-AM) hydrogels, the P(AA-AM-AMPS) hydrogels show a much greater swelling ratio, up to 509 g/g in distilled water. This characteristic is essential for enhancing soil water retention, which in turn promotes plant development. This copolymer is injectable, which enables it to enter deeper layers through tiny fissures in sand or soil particles, in contrast to conventional superabsorbent polymers (SAPs), which are administered as granules or powder. This makes it easier for nutrients and water to reach plant roots directly. By managing the crosslinking behavior, injectability is attained, allowing enough time for injection procedures prior to gelation. The copolymer can penetrate wet sandstones and securely attach them together because of its excellent wet adhesive qualities. Because of this, it shows promise for use as plant roots in sandstone fixing and water retention. Strong wet adhesion is promoted by its functional groups interacting directly with substrates through the replacement of interface water. Ester bonds may be hydrolyzed in hot water (80 °C) to break down the cross-linked structure of P(AA-AM-AMPS) to create a sol. New cross-linking agents can then be added to this sol to re-gel it, enabling material recycling. While injectable superabsorbent resins offer advantages, their application in agriculture often involves high moisture levels, which present a challenge in achieving effective underwater crosslinking while maintaining high water absorption. The danger of macromolecules diffusing into the water during underwater gelation can be reduced by raising the copolymer or cross-linking agent concentration. Though it tends to stabilize over lengthy durations (e.g., 8–10 h), the adhesion strength to substrates like aluminium might diminish with prolonged immersion in water (e.g., from 2 h to 4 h), despite being robust at first [117]. Due largely to their remarkable capacity for water absorption and retention, hydrogels, also known as superabsorbent polymers (SAPs), have demonstrated encouraging uses in agriculture. But a few things can affect how successful they are. SAPs greatly increase the soil’s and sand’s ability to retain water. According to experiments, sand with 0.1 weight percent SAPs were able to hold onto water longer than sand without SAPs; after nine days, the latter lost almost all its water content, while the former was still able to hold onto 10 weight percent. Similarly, compared to plain soil, which hardened and broke after 20 days, soils containing SAPs absorbed more water at first and kept their wet, continuous shape. The hydrogels significantly improve the germination of seeds. Research using maize seeds showed that seeds treated with SAPs had much greater germination energy than seeds not treated with SAPs. This is explained by the abundant water supply that the SAPs’ capacity to absorb and hold onto a lot of water provides. SAPs aid in the development of immature plants in addition to germination. After 15 days, soil with 0.2 weight percent SAPs had a positive impact on the plant weights of the roots and leaves, suggesting that they might aid in the overall growth of the plant. SAPs have a lot of promises for agricultural use in dry and desert areas, where water shortage is a big problem because of their excellent water retention qualities. The water’s specific conductance has a significant impact on SAPs’ water absorbency. When the specific conductance of the water increases, especially below 500 μs/cm, its absorbency rapidly diminishes. The efficacy of SAPs may be diminished in fluids with a higher electrolyte concentration, such as tap water, river water, or rainwater, which differ in their electrolyte composition, even if the decline is only marginally more than this threshold [118]. Strawberries’ preservation effect is greatly enhanced by the *Perilla frutescens* L. essential oil (PLEO) hydrogel beads, which result in a decreased decay rate (15.71%) after 5 days of storage when compared to control groups. This is explained by PLEO’s antioxidants and antibacterial qualities, which preserve fruit quality and increase shelf life. The hydrogel beads work well to prevent weight loss (3.29%) and preserve the toughness of strawberries (1.75 kg/cm^2^). This suggests that the hydrogel maintains the texture of the fruit and helps stop moisture loss. Additionally, the treated strawberries maintained higher levels of total phenols and organic acids, which are essential for antioxidant capacity and nutritional value. Strong antibacterial activities were indicated by the considerable reduction in germs of the hydrogel beads. Since microbial contamination is the main cause of spoiling, this is a crucial process by which the hydrogel keeps the strawberries fresh. The antioxidant qualities of PLEO, which include its high monoterpene and sesquiterpene content, aid in scavenging free radicals and postponing the ageing and degradation of fruit. According to sensory assessments, strawberries treated with PLEO hydrogel beads had better color and flavor. Additionally, the hydrogel preserved the fruit’s firmness and general appeal to consumers. A safer, more natural, and less harmful substitute for chemical preservatives that might leave residues on fruits is the use of plant essential oils like PLEO. Hydrogel beads are made using microencapsulation technology, which helps enclose active ingredients in a protective matrix and protects them from harmful environmental factors, including light, temperature, and oxidation. Additionally, this allows for the regulated release of PLEO components, guaranteeing ongoing antibacterial and antiseptic properties. Although the sensory performance was generally excellent, the treated strawberries’ odour was slightly lacking, most likely because PLEO has a unique fragrance. Customers who are sensitive to the scent of the essential oil may find this to be a factor in their approval. External variables might adversely affect the efficacy of essential oil molecules, necessitating microencapsulation to safeguard them. Although efficient, achieving maximum encapsulation efficiency requires rigorous research and optimization of the encapsulation process, including variables like sodium alginate content, chitosan concentration, and core-to-wall ratio [119]. Water management and soil conditioning are the two main benefits that the biodegradable water hyacinth cellulose-graft-poly(ammonium acrylate-co-acrylic acid) polymer hydrogel (PHG) provides for agricultural applications. Nevertheless, certain features also provide possible disadvantages or things to think about. In distilled water, the hydrogel may expand up to 165 times its own dry weight, demonstrating its tremendous absorption capacity. This enables it to hold onto a sizable quantity of soil moisture. This capacity to retain moisture is essential for agricultural water efficiency because it can delay the release of absorbed water, extending the time that it is available to plants. Research revealed that hydrogel-amended soil was better at retaining moisture than unamended soil. The hydrogel’s biodegradability is a major benefit over synthetic hydrogels, which are frequently non-biodegradable and may be hazardous to the environment. The cellulose-grafted copolymer’s biodegradability in soil was demonstrated by degradation tests, which revealed a greater mass loss in comparison to the copolymer without cellulose. The hydrogel was shown to be broken down by soil microbial isolates, as evidenced by the increased ammonium ion buildup in the cellulose-grafted copolymer. Soil microbial isolates hydrolyze amide-N as part of the degradation process, which raises the soil’s mineral-N concentration. Water hyacinth, an invasive non-native plant that poses serious environmental and economic challenges in many areas, including Kenya, is used in the synthesis of this hydrogel. An efficient and advantageous method of controlling this infamous plant is to use water hyacinth as a raw material, which lowers production costs and gives the hydrogel biodegradability. Because of its capacity to retain water and its biodegradability, hydrogel may find use as a slow-release fertilizer carrier and soil conditioner. Amide-N is hydrolyzed to increase the mineral-N content of the soil, which is good for plant development. The type, concentration, and presence of ions all affect the hydrogel’s ability to swell (water absorbency). Particularly with multivalent cations like Al^3+^ and Ca^2+^, which can neutralize the charge on the hydrogel and result in a more cross-linked network with less water retention, the water absorbency decreased in salt solutions. The hydrogel’s water absorbency is also pH-sensitive; at low pH values, less swelling is seen because of the large percentage of undissociated -COOH groups. Because of this pH sensitivity, the pH of the soil might affect how well it performs. Although biodegradable, the rate of decomposition varies. Despite being much shorter than the hydrogel without cellulose (173 weeks), the cellulose-grafted hydrogel’s half-life of 27 weeks still suggests a very sluggish degradation process. A few variables, including the crystallinity of cellulose and the existence of amide-N as a microbial food supply, might affect this rate. Although water hyacinth has been used to save expenses, the high cost of making bio-based hydrogels using cellulose derivatives may still prevent them from being widely used [122]. While using a hydrogel based on biodegradable starch as a seed covering for maize has several benefits, especially in situations where water is scarce, there are also certain disadvantages and restrictions. The rate of seed emergence was greatly accelerated by the hydrogel coating, particularly in the presence of moderate water stress (77% field capacity, FC). This suggests that when the water supply is less than ideal, hydrogel aids in seed germination. When there is enough moisture present, such as after irrigation, the hydrogel collects water; when the soil dries up, it gradually releases the water. Even after the surrounding soil has dried, coated seeds can still absorb water because of the long-term retention and gradual release of water close to the seed. This guarantees a steadier supply of water for early seedling development and germination. The hydrogel is biodegradable since it is made of potato starch that has been modified with succinate. Compared to synthetic superabsorbent polymers (SAPs), which do not break down and may be hazardous to the environment, this is a more environmentally friendly option. The selected hydrogel has an excellent ability to absorb water and is simple to produce. At lower water supply levels (65% FC) and higher water supply levels (92%, 82% FC), the hydrogel covering only slightly improved seed emergence. When soil moisture levels are high, the hydrogel’s ability to retain water is no longer necessary. On the other hand, if the water content is too low, the hydrogel cannot absorb enough water or lose it to the dry soil too soon to be of any use to the seed. The dry weights of the roots and shoots of maize plants after 14 and 21 days of growth were not substantially impacted by the hydrogel covering. This implies that the hydrogel’s contribution to the plant’s water needs diminishes during seed emergence and early root growth. This is probably because the tiny amount of water that the hydrogel surrounding the seed retains is less significant when the plant’s growing root system has access to water from a bigger volume of soil. When plants were removed to assess biomass, fungus development was seen on the surface of several seeds. The hydrogel’s ability to retain water may be impacted by its capacity to act as a substrate for fungus due to its biodegradability. Fungicide treatment as a component of the seed coating mixture could be required for this. Compared to pure water, a hydrogel’s ability to absorb water is greatly diminished in saline conditions. Moreover, it has very little capacity to absorb water when subjected to mechanical strain. These drawbacks show how little the hydrogel material can withstand the mechanical and osmotic pressures seen in agricultural soil.

Studies relevant to a certain species are necessary since the impact of hydrogel seed coverings might differ depending on the kind of seed. For example, whereas hydrogel coatings enhanced maize emergence under mild stress, other research revealed that they may hinder seed aeration and decrease emergence in other crops, such as Common Bean and Russian Wildrye, because of the extra water close to the seed [123]. Superabsorbent hydrogel (SAH) based on poly(vinylpyrrolidone) (PVP) has a few benefits for agricultural applications, especially for plants under drought stress, but it also has certain drawbacks that need more research. By raising soil moisture content, hydrogel aids in soil water retention. This is essential for maximizing the use of water resources in agriculture, particularly when there is a shortage. It can absorb water hundreds to thousands of times its mass, demonstrating exceptional swelling capacity and water retention qualities. Over time, the hydrogel makes it easier for vital fertilizers like potassium, phosphate, and nitrogen to be released gradually. By using this controlled release technique, the needless environmental traditional fertilizers’ contamination is reduced. This results in increased nutrient efficiency, a consistent flow of nutrients, less nutrient runoff or leaching, and less upkeep needed for fertilizer application. When the hydrogel was sprayed on Pisum sativum plants that were experiencing drought stress, the plants’ overall performance was greatly enhanced. It successfully reduced the negative impacts of drought stress by increasing biomass, chlorophyll content, and photosynthetic efficiency. Shoot length, root length, fresh and dry weights, and leaf number were among the morphological characteristics that hydrogel enhanced. Through an increase in yield indices including grain quantity, grain weight, 100-grain weight, and pod number, it also improved reproductive activity. Through the reduction of hydrogen peroxide and malondialdehyde levels and the enhancement of carotenoid and chlorophyll pigments, the hydrogel showed a protective role against oxidative stress. Given that the hydrogel is made with easily accessible and reasonably priced basic ingredients, the production technique appears to be both scalable and reasonably priced. The untreated hydrogel initially retained more water and continued to do so throughout the observation time in comparison to the treated hydrogels, even though the treated ones displayed good swelling. In certain cases, the hydrogel’s internal structure may be disrupted by the alkali treatments (NaOH or KOH), which might result in faster water release and reduced retention percentages. Despite being biodegradable, the PVP-based SAH’s long-term destiny and its environmental effects in agricultural ecosystems—particularly regarding non-target species and soil microbiomes—need careful research. As a model crop, Pisum sativum was the focus of the current investigation. To show the hydrogel’s wider application, its effectiveness must be assessed over a larger variety of economically significant crop species and cultivars. The study recommends investigating more environmentally friendly multipurpose hydrogels that have even greater water retention, the capacity to act as carriers for anti-phytopathogenic chemicals, and the ability to release other vital fertilizers (such as calcium, magnesium, and iron) gradually [124].

Dipankar Das et al. made the superabsorbent biodegradable hydrogel from natural materials carboxymethylcellulose sodium salt (NaCMC) and hydroxyethyl cellulose (HEC), using citric acid (CA) as a crosslinker [121]. In comparison to unmodified soil (UMS), which has a porosity of 53% and a density of 1.16 g/cm^3^, modified soil with hydrogel has an excellent porosity of 57% and a lowered density of 1.06 g/cm^3^, which allows plants (tomato and cucumber) to use water more efficiently, which is essential for their growth and health. Significant increases in growth and photosynthesis were seen in plants cultivated in the amended soil, suggesting that the hydrogel facilitates better nutrient and water absorption [121]. Paola Calcagnile et al. introduced a biodegradable superabsorbent poly (lactic acid)-cellulose hydrogel for agriculture that helps control the release of potassium nitrate fertilizer and water efficiently [120]. Experiments on two varieties of Mediterranean crops revealed that this substance enhances the soil’s capacity to retain water, particularly in sandy red soil, without impairing plant development, indicating that agriculture is advantageous, especially in regions with scarce water supplies. Therefore, agriculture-friendly hydrogels are the candidates for boosting the production of crops.

### 3.3. Application in Biomedical Engineering

**Figure 11:**
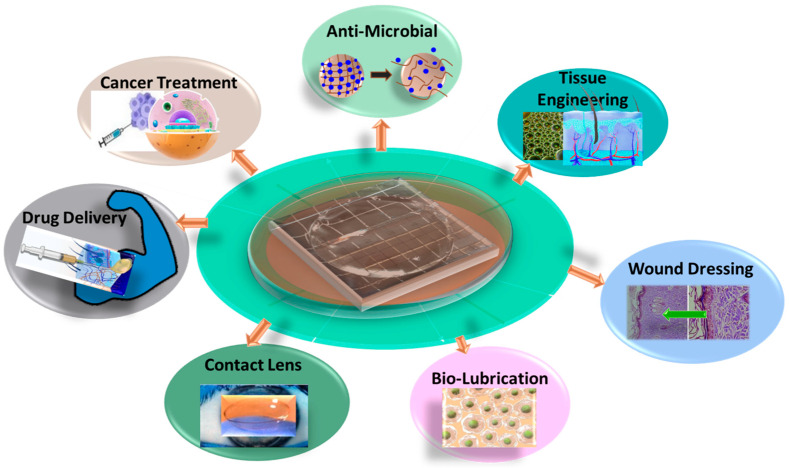
Hydrogels in different biomedical applications.

Biomedical engineering integrates science and engineering to enhance healthcare by creating novel goods and services, such as simulation software and medical equipment. Prosthetics and monitoring devices are examples of health instruments that have advanced as a result of this field’s convergence of biology, medicine, and technology [125]. Biomaterials that can provide cells with particular mechanical, biological, and structural signals are in greater demand. Enhancing these materials’ capacity for regeneration in a variety of applications requires this customization [126]. Hydrogels’ special qualities and adaptability have made them indispensable materials in different fields of biomedical engineering, as shown in Figure 11. The high-water content of hydrogels makes them very similar to the extracellular matrix (ECM) that is naturally present in biological tissues. This resemblance aids in establishing an atmosphere that is favorable for tissue regeneration and cell proliferation. Hydrogels’ mechanical and structural characteristics can be modified to resemble those of real tissues. This resemblance is essential for tissue engineering applications, where the scaffold’s mechanical characteristics might affect tissue development and cell behavior. Since hydrogels may interact with biological systems without triggering an unfavorable immune response, they are frequently biocompatible. This characteristic is essential for uses where materials need to be safe for the body, such as medicine delivery and cell encapsulation [127]. The development of materials with improved cytocompatibility and controlled degradation, which are essential for their use in biomedical domains including drug delivery and tissue engineering, has been the focus of recent developments in dynamic hydrogels. The dynamic hydrogels are safe for biological applications because of their outstanding cytocompatibility. Their stability and degradation rates may be adjusted by temperature and pH, providing adaptability for a range of biomedical applications. Their degradation can be initiated by selective chemical cleavage of trithiocarbonate groups [128,129].

Chunqing Lin et al. used L929 fibroblast cells and the MTT test to assess the cytocompatibility of the P(NIPAM-co-DAAM)-b-PAM-b-P(DAAM-co-NIPAM) hydrogels [130]. Relative cell viability was assessed after 24, 48, and 72 h of incubation in hydrogel macerating solutions. All studied hydrogels (H2, H4, and H6) and various mass ratios of H4 to medium (2.5, 5.0, and 10.0 mg/mL) demonstrated cell viability of more than 95%. These results were further supported by live/dead labelling, which revealed no discernible red fluorescence (dead cells) and demonstrated that rounded cells could stick to the hydrogel and multiply over time. After 12, 24, and 48 h of incubation, more than 95% of the cells were found to be viable. These results show that hydrogels may act as a platform for cell carriers and are non-cytotoxic to L929 cells, indicating their potential in biomedical applications.

Reversible addition–fragmentation chain-transfer (RAFT) polymerisation was used to create the triblock copolymers, which included a trithiocarbonate group in the midst of the polymer chains and were intended to be degradable hydrogels. Nucleophilic reagents such as ethanolamine may cleave this group, causing deterioration. According to the experimental findings, hydrogel H4 remained slightly inflated in the absence of ethanolamine but broke down in two hours when it was present. This demonstrates that the hydrogel’s breakdown is made possible by the cleavage of the trithiocarbonate groups [130]. Hydrogels can be engineered to release genes or medications in a regulated way. Because it enables prolonged release over time, this capacity is very advantageous for targeted medicines and can improve therapeutic efficacy. In additive manufacturing methods, hydrogels may be utilized to construct intricate structures that are packed with cells. This capacity creates new opportunities for the development of scaffolds and implants that are customized to meet the demands of each patient [131,132]. Table 4 includes some hydrogels effectively used in different fields of biomedical engineering. Numerous hydrogels can react to changes in pH or temperature. Utilizing this responsiveness, intelligent materials that adjust to their environment may be developed, enhancing their usefulness in biological applications [133]. Polymers in hydrogel production for biomedical applications include synthetic polymers—Poly(meth)acrylates, Polyethylene Glycols (PEGs), Poly(vinyl alcohols) (PVAs), Poly(vinylpyrrolidones) (PVPs), Poly(lactic-co-glycolic acid) (PLGA), Poly(urethanes), and natural polymers—Hyaluronic Acid, Alginate, Chitosan, etc. Hydrogels also contain natural proteins, including albumin, collagen, elastin, and fibrin, because of their capacity to promote cell development and biocompatibility. The characteristics of both natural and manufactured polymers are frequently improved via chemical modification. Modifications can increase their porosity, elasticity, mechanical strength, and drug release properties, which makes them more appropriate for particular biological uses [134]. Gradient hydrogels are specialized materials that exhibit a non-uniform structure, meaning their properties change gradually over space rather than being uniform throughout. Traditional hydrogels, on the other hand, have an isotropic (uniform) structure. These hydrogels can have gradients that are continuous (smooth transitions) or abrupt (step-like shifts). This adaptability enables customized qualities that may be changed for certain uses [135,136,137]. Gradient hydrogels are very helpful in industries like pharmaceuticals for controlled-release medication delivery systems and tissue engineering for usage as scaffolds. Understanding intercellular interactions can be aided by their intricate internal architecture [138]. Gradient hydrogels are frequently developed with inspiration from natural materials to mimic their advantageous qualities for use in medicinal settings. Since a thorough knowledge of these methodologies is still missing, recent research has concentrated on the strategies utilized to build and characterize these gradients [139]. Drugs can be released from hydrogels under regulated conditions. Because of their porous nature, medicinal medicines can diffuse gradually, extending the drug’s residence duration at the target location and reducing systemic adverse effects. This is especially helpful for medicine delivery systems when accurate dosage is essential [140,141]. Hydrogels are scaffolds used in tissue engineering that promote cell adhesion and growth. Growth factors and extracellular matrix proteins, which promote tissue regeneration, can be included in their design. Hydrogels’ adjustable mechanical characteristics enable customization according to the particular tissue type being addressed [142]. Because hydrogels can keep an environment wet, which is necessary for the best possible healing, they are utilized in wound dressings. They can also provide a barrier against infections while allowing for gas exchange. To further shield the wound site, some hydrogels are made to release antibacterial chemicals [143]. Numerous hydrogels are capable of in situ gelation, which allows them to be injected as a liquid and subsequently harden at the intended location. This characteristic facilitates administration and guarantees that the hydrogel adheres to the unique anatomy of the region being treated, improving. Hydrogels could modify physiological reactions by interacting with biological systems. For instance, by affecting biological processes like cytokine expression and macrophage behavior, certain hydrogels might reduce inflammatory reactions or encourage healing [134].

A unique 3D-printed gelatin–alginate hydrogel was developed by Yuan Ma et al. to support stem cells for improved healing by mimicking the rigidity of skin. The stem cells grew and moved more efficiently with the support of this scaffold, which demonstrated high stability. In order to address skin injuries and possibly other tissue problems, the novel scaffold enhanced blood vessel formation and wound healing [144]. By concentrating on their structure and interactions with cancer cells, Aleksandra A. Patlay et al. created stable and non-toxic hydrogel films and nanoparticles to deliver cancer medications using low-esterified pectin [145]. These materials showed good properties for use in brain tumor surgery, did not damage healthy cells, and could inhibit the development of glioblastoma cells, making them safe for targeted medication administration. The study also looked at ways to create pectin-based small particles (NPs), which can be improved to more effectively target certain cancer cells and ensure patient safety and efficacy.

**Table 4:** Application of hydrogels in different fields of biomedical engineering.

Hydrogel Composition	Properties	Field of Application	Function	Reference
Alginate/Aloe vera	Hydrogel is transparent in both dry and wet state	Biomedical Engineering	Hydrogel films potentially explored as wound dressing for dry and exuding wounds.	[146]
Pectin/Sodium alginate/Gelatin	Hydrogel shows ideal printability and shape fidelity	Tissue engineering	Hydrogels show repairing osteochondral defects due to its anti-inflammatory effect and fibrosis inhibition.	[147]
Gelatin–alginate hydrogel	Hydrogel exhibits good stability and biocompatibility	Skin regeneration	Hydrogel shows better angiogenesis and wound healing effects.	[144]
Pectin/Cellulose	Hydrogel is stable and possesses favorable biocompatibility	Hemorrhage	Within three minutes, the hydrogel-treated liver incision had reduced bleeding.	[148]
Silk fibroin/collagen	The 3D scaffold displaying biochemical gradients along longitudinally oriented microchannels.	Tissue Engineering	Hydrogel can be used to develop advanced devices for treating severe nervous system injuries	[149]
Gelatin/ methacrylamide	Epidermal Hydrogel shows tunable mechanical, degradation, and biological properties	Epidermal Tissue Engineering	Hydrogels show superior cell viability (>90%).	[150]
Sulfate/poly (γ-glutamic acid)	Hydrogel exhibits great viscoelastic, toughness	Cartilage tissue engineering	It features a gradient porosity structure in addition to high biocompatibility (almost no dead cells in the live/dead staining experiment).	[139]
Multidimensional crosslinking gradient PNIPAM/Laponite	Hydrogel is gradient showing a slower release rate than non-gradient hydrogel	Drug delivery	Compared to the non-gradient hydrogel, the drug release rate is 0.71 times slower.	[138]
Silk/PEDOT: PSS	Hydrogel shows good shear-thinning properties	Cell transplantation	High capacity to facilitate the development of intricately linked neural networks from encapsulated neurones and astrocytes produced from human induced pluripotent stem cells (hiPSCs).	[151]
Selenium/Polyurethane	Thermo-Sensitive Hydrogel	Diabetic wound healing	Hydrogel has a strong wound-healing effect by encouraging collagen production, maturation, and reepithelialization.	[152]

Alginate/Aloe vera hydrogel films have been created for use in biomedicine, namely for medication delivery and wound healing. Despite certain drawbacks, they provide a number of benefits because of their intrinsic qualities and the complementary impacts of their constituent parts. Since hydrogels made from natural polymers like alginate and aloe vera are biocompatible and biodegradable, they are being studied extensively for use in biomedical applications. Their characteristics are comparable to those of human tissues. In aqueous liquids, hydrogels—three-dimensional networks of hydrophilic polymers—can expand without losing their structural integrity. They can absorb exudate from wounds thanks to their swelling ability, which stops maceration. Aloe vera improves the films’ hydrophilic qualities and increases their capacity to absorb water. Natural hydrogels’ smoothness, high water content, elasticity, and malleability make them appealing for treating wounds. They offer a wet environment that aids in healing and prevents the wound from drying up. The alginate films’ calcium ions, which are liberated by ion exchange with sodium ions in wound exudate, help to create a hydrophilic gel that keeps the surrounding area wet and has haemostatic qualities, which make them effective for bleeding wounds. For wound healing, alginate gels’ ability to integrate and release a variety of therapeutic ingredients is essential. Aloe vera promotes collagen synthesis and fibroblast proliferation while also having antibacterial, antiseptic, and anti-inflammatory qualities. These substances have the ability to permeate the wound bed and promote healing. High transparency in the films created is crucial for wound healing since it enables visual examination of the damage without the need to remove the film. The aloe vera ingredient enhances the transparency of the film. During In Vitro degradation testing, the study found that films with a greater Aloe vera content significantly lost weight, suggesting that they are more prone to hydrolytic disintegration. The breaking of degradable connections caused by enhanced water penetration into the hydrogel network is the cause of this accelerated breakdown. A biological use requires degradation, but a pace that is too quick might shorten the film’s lifespan or prevent continuous release [146]. The use of hydrogels in tissue engineering is growing because of their special qualities that resemble the extracellular matrix found in nature. But there are certain disadvantages to their use in addition to some major benefits. By precisely mimicking the physical and physiological characteristics of the natural extracellular matrix, hydrogels can provide an environment that is conducive to tissue formation and cell proliferation. For example, pectin-based hydrogels have demonstrated biocompatibility and biofunctionality, promoting metabolic activity and cell proliferation while preserving the distinctive spherical shape of healthy chondrocytes. For tissue engineering applications to be effective, they make it possible for excellent cell survival and consistent cell dispersion across the scaffold. Hydrogels’ mechanical strength and toughness, for example, may be adjusted to meet the needs of different tissues and physiological environments. With their perfect printability and form fidelity, hydrogels may be used in 3D bioprinting processes to produce complex, biomimetic tissue structures. Hydrogels made from agricultural byproducts, such as pectin from potato pulp, can be broadly accessible, affordable, and sustainable substitutes for items made from fossil fuels. Some hydrogels, such as those that include pectin RG-I, have shown promise in reducing fibrogenesis and inhibiting inflammation, which is advantageous for tissue regeneration, particularly in diseases like osteochondral abnormalities. The ability of pectin-based hydrogels to absorb and hold onto water is crucial for cartilage tissue engineering scaffolds because it replicates the natural aqueous extracellular matrix environment. Certain ingredients, such as pectin RG-I, can increase the liquidity of pre-cross-linked hydrogels, which may cause the scaffold to disintegrate during In Vitro growth. This further emphasizes the difficulty of regulating viscosity to guarantee uniform cell dispersion free from clumping or gravitational settling. Although the development of new tissue requires breakdown, an imbalance may be detrimental. If the disintegration rate is too quick, the scaffold may not properly hold cells or provide mechanical support for long enough. The study noticed that certain hydrogels grew exceedingly soft over time, albeit they retained form and integrity. Research on certain biological elements that may be added to bioinks to concurrently promote osteogenesis and chondrogenesis is scarce, despite the success of bioprinting [147]. Hybrid hydrogel, which is made of cellulose and pectin, shows a number of encouraging haemostasis properties in addition to several issues that warrant more study. Within three minutes, hydrogel-treated liver wounds show decreased bleeding due to the hydrogel’s strong haemostatic action. It successfully prevents blood loss, most likely as a result of the bleeding tissue’s exterior covering, quick sealed filling, and endogenous coagulation. By speeding up the erythrocyte and platelet aggregation process, the hydrogel creates a barrier that stops tissue fluid and cell leakage. In order to aid in haemostasis, its nanostructures both stimulate platelets and absorb percolate. For ten days, mice’s primary organs (heart, liver, spleen, lung, and kidney) do not exhibit any pathological lesions or evident abnormalities due to hydrogel, showing that it is non-toxic. It encourages cell division; treated cells proliferate more quickly, and 3T3 cells have a survival rate of more than 95%, indicating good biocompatibility. The hydrogel exhibits favorable immunological compatibility and avoidance of macrophage development, as seen by the high proliferation of RAW 264.7 cells, which are macrophages. Blood clot formation is accelerated by the hydrogel’s dense network structure and spongy, porous characteristic, which increase surface area and improve the ability to absorb water liquids. It preserves the crystal structures of Cellulose I and II as well as its chemical linkages (such as OH, CH, C=O, -CH_2_, CO, C1-H, and β-glycosidic bonds). With pectin marginally raising its breakdown temperature, the hydrogel exhibits good thermal stability, holding steady even when body temperature stimulation occurs (30–50 °C). When employed alone, pectin, a component of the hydrogel, may lose its mechanical qualities and integrity under physiological settings. By mixing it with cellulose, the composite hydrogel seeks to solve this issue. Even though the particular pectin/cellulose hydrogel exhibits outstanding biocompatibility, its wider application is limited because oxidized regenerated cellulose, a substance frequently found in hydrogels, can still result in postoperative abscesses, tumors, or haematomas. More research is needed to determine how hydrogel affects skin lipids and skin barrier repair [148]. By combining the SF/Col hydrogel with directed freezing and customized 3D printing, scaffolds that exhibit continuous biochemical gradients along longitudinally orientated microchannels may be created. This is appropriate for nerve healing and closely resembles the circumstances for axonal sprouting in vivo. An important development in brain tissue engineering is this cue integration. For up to 28 days, the hydrogel can maintain its bioactivity while controlling the release of nerve growth factor (NGF). In order to encourage nerve regeneration, this prolonged release is essential. Ionic attraction between silk and NGF, particular interactions between bioactive compounds and hydrophobic areas of silk fibroin, and decreased chain mobility are all thought to be responsible for the regulated release kinetics. The bioactivity of NGF within the scaffold is preserved by the low temperature maintained during the directed freezing and lyophilization processes. Since collagen is a key structural component of the extracellular matrix, adding it to the silk fibroin solution increases the scaffold’s biocompatibility. Additionally, it makes the silk solution more viscous, which helps to create a steady gradient inside the mould. The SF/Col scaffold works in concert to support directed nerve regeneration and axon elongation, especially when it is designed with both NGF gradients and orientated microchannels (G-NGF + OS). Compared to either cue alone, this combined direction is more effective. The potential for therapeutic translation of the G-NGF + OS scaffold was demonstrated by in vivo experiments that demonstrated its ability to speed up the recovery of sensory and motor functions in rats with sciatic nerve damage. Customized biomedical devices with intricate 3D structures and biochemical cues may be created thanks to the robotics-based 3D printing method, which makes programming simple and allows for exact control of flow rates. Although the technique has several benefits, it is still difficult to create 3D scaffolds that incorporate topographical guiding cues and biochemical gradients. Diffusion-based techniques have trouble producing continuous gradients in 3D scaffolds that are centimetres long because of capillary effects, and conventional techniques like as microfluidic devices are difficult to scale for direct in vivo implantation. The existing approach requires a specially designed 3D printer with two separate propulsion systems and accurate flow rate control. The SF/Col scaffold broke down more quickly in the presence of protease XIV than it did in PBS, suggesting that enzymatic activity in the biological milieu may cause degradation to occur more quickly. Although hydrogel greatly aided in the regeneration of sensory neurones, the rise in retrogradely labelled motoneurons was only a trend and did not reach statistical significance when compared to other groups. This implies that particular motor neurone regeneration may still be difficult even when overall functional recovery has improved [149]. For epidermal tissue engineering, gelatin methacrylamide (GelMA) hydrogels provide a number of benefits, chief among them being their biocompatibility and adjustable characteristics. There are several restrictions, nonetheless, especially with regard to the thickness and complete development of the restored epidermis. By altering the hydrogel concentration and photopolymerization duration, GelMA hydrogels provide fine control over their mechanical and degrading properties. This adaptability is essential for meeting the various needs of various body locations and wound kinds. Degradation periods can vary from a few days to several months, and their elastic and compressive moduli can be varied from a few kPa to hundreds of kPa. They are perfect candidates for skin regeneration because of their strong tunability. Compared to conventional collagen hydrogels, which frequently break down within a month, higher concentrations of GelMA (e.g., 20%) show noticeably slower rates of degradation and can last up to eight weeks, which is advantageous for long-term wound healing. GelMA hydrogels’ intrinsic biocompatibility is demonstrated by their excellent cell viability (over 90%) at all concentrations. They efficiently promote the adhesion, growth, and differentiation of keratinocytes. Improved cell adhesion and proliferation are the results of higher GelMA concentrations. Crosslinked GelMA’s transparency makes it simple to see how cells behave when they are seeded or encapsulated on the hydrogel. Similarly to the normal epidermis, GelMA hydrogels can aid in the restoration of a multilayered epidermis with sufficient barrier properties. GelMA hydrogels are photocrosslinkable polymers that can undergo a permanent solidification process by the chemical reaction of methacrylamide groups. When exposed to light, the hydrogel prepolymer solution may be applied to wound sites of different forms and quickly crosslinked in situ, enabling the best possible moulding to the contour of the wound. This is especially helpful for trauma wounds that are large and irregular. Although GelMA hydrogels facilitate the development of a stratified epidermis, the immature terminal differentiation of HaCaT cells may result in a slightly disorganised structure in the reconstituted epidermis. Due to either immature terminal differentiation or fewer degrees of stratification, the resistance values of the rebuilt epidermis on GelMA were found to be lower than those of human skin. The less mature differentiation shown may be due in part to the fibroblasts’ lack of paracrine signalling during in vitro culture, indicating that in vivo settings may be necessary for complete maturity [150]. With its ability to replicate the natural bioelectrical environment of neural tissue, the electrically conductive injectable silk/PEDOT: PSS hydrogel has several benefits for improving the creation of neural networks, especially when it comes to spinal cord repair. But there were also certain shortcomings and areas that needed more research. The hydrogel can replicate the natural bioelectrical environment because of its electrical conductivity, which is in the range of around 0.2–1.2 S/m and comparable to that of spinal cord (1–10 S/m). This is essential for fostering synaptic function and cell-to-cell transmission. In order to promote the formation of functioning neurones, it can also offer an appropriate platform for therapeutic localised electrical stimulation. The hydrogel formulations have a flow point below 100 Pa, have strong shear-thinning qualities, and are readily injected with a conventional hand force. Cell transplantation benefits from this less intrusive method. Under physiological settings, the hydrogel’s elastic modulus (~10–60 kPa) remains comparable to that of the spinal cord, demonstrating its flexibility. By embedding iPSC-derived neuronal cells, this mechanical characteristic facilitates the development of neural networks and ensures optimal injectability. Encapsulating hiPSC-derived cortical neurones and astrocytes in these hydrogels resulted in cell survival rates of at least 80%. By encouraging the growth of an intricate, linked network of lengthy axonal processes and accelerating synaptogenesis, hydrogel aids in the maturation of cortical neurones. Due to their softer microenvironment (storage modulus ~1000–2000 Pa) and increased porosity, which promotes the transfer of nutrients and oxygen, the GoPS-modified ECHs in particular demonstrated comparatively enhanced cell survival and strong axonal projections. In comparison to unmodified versions (~30–80%), GoPS-modified ECHs showed better structural recoverability (~70–90%), which is crucial for preserving the integrity of the cellular network following injection. Even after 31 days of incubation in PBS, the hydrogels showed increased mechanical resilience and retained their capacity to conduct current, suggesting that they are suitable for long-term cell culture applications including electrical stimulation. Despite being largely biocompatible, the SF/PEDOT06PeG group had possible toxicity problems, as seen by the round and strained appearance of cells when they were encased within and cultivated on its surface. This implies that PeGDE could become more reactive in a humid environment, which could result in higher cytotoxicity and worse side effects. PEDOT: PSS’s hydrophilic PSS chains raise questions about its cytocompatibility since they have the potential to breakdown or delaminate and have cytotoxic consequences. Neuronal network development was worse in the hydrogel formulation containing the most unaltered PEDOT: PSS (SF/PEDOT12). This can restrict the functional connection between neurons because of its greater rigidity and more compact hydrogel network with reduced porosity [151]. The selenium-containing polyurethane (SePU) thermo-sensitive hydrogel (SePU/HBC) exhibits promise characteristics for diabetic wound healing, but like any treatment, it has both advantages and potential problems. In diabetic wounds, hydrogel dramatically accelerates collagen synthesis, re-epithelialization, and wound maturation. Additionally, it promotes skin appendage and blood vessel regeneration, both of which are essential for efficient healing. Through the unfolded protein response (UPR), SePU/HBC reduces endoplasmic reticulum stress (ERS) and prevents apoptosis. This is especially helpful in the hyperglycaemic setting of diabetes, which frequently makes cellular dysfunction and ERS worse. It greatly lowers inflammatory cell infiltration and inhibits pro-inflammatory cytokine output, while enhancing anti-inflammatory genes, ultimately regulating inflammation. Human skin fibroblasts (HSFs), which are vital for tissue development and wound healing, proliferate, migrate, and adhere when hydrogel is present. Because SePU/HBC is thermosensitive, it quickly gels at physiological temperatures, making injections and administration into the wound site simple. With no discernible cytotoxicity to HSFs in vitro and no discernible inflammation in major organs in vivo, it demonstrates high biocompatibility and safety. The hydrogel exhibits improved flowability and self-healing properties while remaining stable in the face of external stresses. Cell development is compatible with its porous structure. One of the main ingredients in hydrogel, selenium, has bimodal biological effects that vary with concentration. This indicates that in order to attain the best possible treatment results, careful concentration control is required. For example, compared to higher doses, smaller concentrations (0.2 and 0.5 µg/mL) had less proliferative effects on HSFs. In the early healing phase, hydrogel promotes fibroblast migration and tissue creation by exhibiting a quicker initial breakdown rate than HBC alone. However, by day 14, the degradation rates converge. Although the long-term degradation profile and its consequences for prolonged release after 14 days are not specifically mentioned as a disadvantage, it is implied that the benefit may lessen after this time. Although the findings show that SePU/HBC has therapeutic efficacy in reducing ERS and preventing apoptosis through ATF6-mediated UPR activation, more investigation is required to fully understand the molecular processes and how they interact with other cellular signaling pathways. This implies that our existing knowledge is not all-inclusive [152].

### 3.4. Application in Electronic Devices

**Figure 12:**
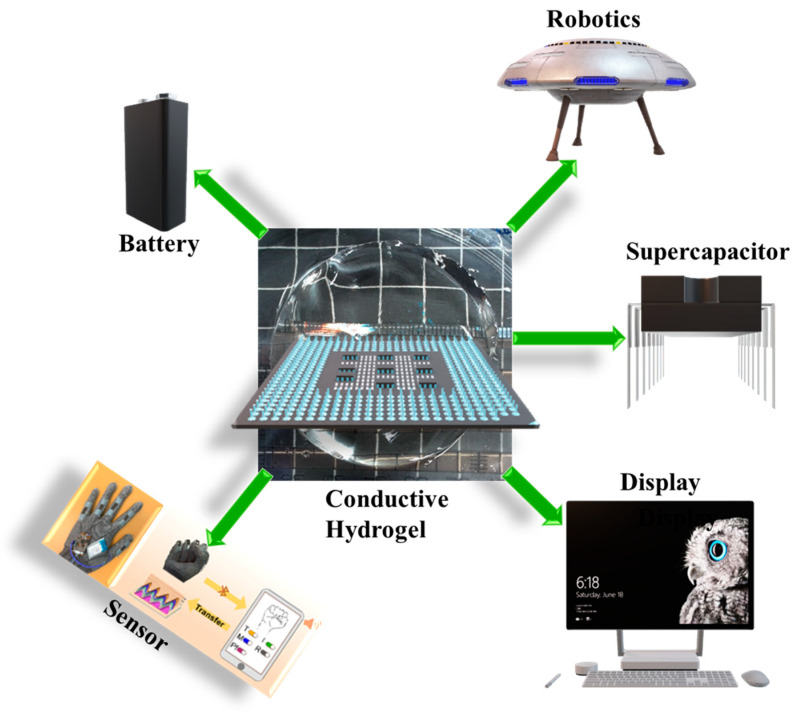
Application of hydrogel in different electronic devices.

The electrical conductivity of traditional conductors, such as copper (Cu), aluminium (Al), and silver (Ag), has led to their widespread usage in electronic equipment. On an electrical conductivity scale of 0 to 100, silver has the highest rating (100), followed by copper (97) and gold (76). Their performance and use in contemporary technology, however, can be impacted by a number of limitations. Despite having high conductivity, copper may corrode easily, which might reduce its usefulness in electrical applications. Over time, this vulnerability becomes less dependable because of its lack of passivation oxides. The corrosion resistance of aluminium makes it a popular choice, although its conductivity is lower. Defects, including porosity, contaminants, and shrinkage, can result from conventional conductor production methods like extrusion and hot rolling. These flaws may impair the conductors’ functionality and integrity, which might result in electronic device failures [153]. Moreover, traditional electronic devices have a few serious drawbacks that reduce their usefulness and efficacy, especially when it comes to health monitoring. Traditional devices sometimes employ a limited variety of materials, which might limit their adaptability to various settings and uses. The creation of increasingly sophisticated and useful wearable technology may be hampered by this lack of material variety. The dynamic and fluctuating variables that users may experience, such as variations in temperature, humidity, and physical activity levels, are sometimes not taken into consideration by traditional devices. In practical situations, this omission may result in performance that is unreliable. Therefore, these drawbacks highlight the necessity of developing wearable and soft electronics that are more capable of adjusting to the intricacies of human physiology and changing environmental circumstances [154]. Hydrogels are increasingly being used in electrical devices because of their unique properties and benefits to help overcome the restrictions. It is possible to create conductive hydrogels to have favorable electrical characteristics, which are crucial for electronic applications. By adding substances like conductive polymers, carbon-based nanomaterials, and metallic nanoparticles, they can be rendered conductive. The operation of many electronic parts, including sensors and energy storage devices, depends on this conductivity. Hydrogels have high mechanical flexibility, biocompatibility, and self-healing characteristics, which make them appropriate for use in flexible and wearable electronics. Their capacity to mimic the mechanical characteristics of biological tissues makes it possible for them to be more seamlessly integrated into wearable devices and other applications where flexibility is essential. Hydrogels can be processed easily, allowing for straightforward fabrication techniques, which facilitate the development of complex structures in flexible electronics. Hydrogels are used in many different types of electrical devices, including wearable sensors, energy storage systems, touch panels, memristors, textile sensors, batteries and supercapacitors, displays, robotics, and environmental sensors [155]. Figure 12 presents the applicability of different hydrogels in different electric devices.

The main benefits of hydrogels are their flexibility and stretchability. They are perfect for wearable devices that must adjust to the motions of the body since they can bend and stretch without losing their electrical conductivity. The selection of the crosslinking density and polymer matrix frequently results in this flexibility. This characteristic is essential for applications requiring movement and flexibility in flexible electronics, such as artificial muscles and skins. Table 5 listed some recent applications of hydrogels in electronic devices. Numerous hydrogels can safely interact with biological systems and stimulus-responsive environments because they are biocompatible and react to environmental stimuli like pH or temperature changes. This characteristic is especially crucial for applications in medical equipment, including physiological signal monitoring sensors. Their adaptability to uneven surfaces further improves their utility in medical environments [156,157,158,159]. Ionic Conductor Hydrogels are superior ionic conductors because of their water-retentive polymer network. This characteristic enables them to efficiently convey electrical impulses, much like biological tissues do. Water’s conductivity is improved by the presence of mobile ions, which is crucial for electrical applications. Hydrogels are very transparent to visible light because of their optical characteristics, which are comparable to those of water.

Daihui Zhang et al. developed Cellulose Biomimetic Hydrogels (CBH) to mimic the properties of human skin, making them highly suitable for sensor applications [160]. CBH has outstanding stretchability (up to 914.3%), high toughness (up to 4.3 MJ/m^3^), and good elasticity, low modulus, and self-stiffening behavior. For wearable electronics and medical monitoring, these characteristics are essential for integration with delicate biological tissues like skin. The material may be twisted and knotted without injury. With exceptional fatigue resistance and elastomer-like behavior, it maintains an elastic recovery of 93% to 94% following many cycle tests, and even 92.7% following 100 cyclic tensile tests. This shows how durable it is for frequent motion. CBH’s resistance to swelling, a typical problem with other hydrogels that can deteriorate mechanical and electrical capabilities and change the geometry of devices, is a major benefit. Swelling is reduced by the porous, non-swelling cellulose skeleton. With its volume expansion restricted to 10.5% in water and 29.2% in PBS, CBH exhibits noticeably lower swelling ratios than polyacrylamide (PAM), suggesting great efficacy in reducing hydrogel swelling and less susceptibility to environmental fluctuations. Conductive CBH may be created via in situ polymerization of aniline, allowing it to transfer mechanical deformation into electrical impulses. It can track human movements even in an aquatic environment since it is a sensitive and reliable strain sensor. Depending on the strain range, the gauge factor (GF) might be anywhere between 1.7 and 0.5. High sensitivity and dependability are demonstrated by the sensor’s quick reaction times (250 ms) and extremely reversible and repeatable signal responses throughout stretching/releasing cycles. It is appropriate for soft electronics in water-based systems, including diving gear or internal body devices, since it sustains a steady signal output in an aquatic environment. Hela cells sustain great vitality (over 85% at a concentration of 20 mg/mL) when co-cultured with the hydrogel, demonstrating CBH’s strong biocompatibility. Wearable electronics and biological applications require this. CBH wastes a significant amount of energy during the initial loading-unloading cycle, and a clear hysteresis loop is seen. This suggests early structural alterations, including crystalline domain deformation and partial fracture of the supramolecular network. On the other hand, later cycles have better flexibility and smaller hysteresis loops [160]. 2.142. With a notable mechanical stress of 0.29 MPa and an elongation at break of 1385%, the CMM gel shows enhanced mechanical strain. This is explained by a multi-dynamic cross-linking network that is created by interactions at the molecular level, such as electrostatic interactions, hydrogen bonds, and the addition of tannic acid (TA) and MXene. At about 95%, the CMM gel has a remarkable self-healing efficiency. This gives the gel a longer service life and greater resilience to external damage by allowing it to regain its mechanical and conductivity qualities even after being sliced. When two pieces are brought together, conductivity is promptly restored, demonstrating the speed at which the self-healing process occurs. When dissolved in NaCl solution, the gel’s remarkable recyclability allows it to be remoulded into various shapes while maintaining its mechanical and sensing capabilities. This function encourages sustainable growth and drastically lowers electronic waste. As a very sensitive strain sensor, the CMM gel can detect handwriting, individual physiological states, such as breathing, and both small and large body movements. Under cyclic strain tests, it exhibits steady resistance changes and quick reactivity (220 ms/230 ms). Additionally, the hydrogel has antimicrobial qualities, good adhesion, and low-temperature tolerance (it does not freeze at −20 °C and stays flexible). It may also be utilized in all-solid-state capacitors as a solid electrolyte. The gel’s recyclable nature and the utilization of the renewable polysaccharide carrageenan make it a potentially less expensive and more ecologically friendly product. In comparison to the gel without these additions, the elongation at break decreases (from 1880% to 1385%) even though the inclusion of TA and MXene greatly increases mechanical stress. Despite the gel’s strong capacitance retention, PANI degradation causes a progressive drop in specific capacitance after extended usage (2000 cycles for the capacitor application, for example). Even while self-healing works, the repaired area could still have faint traces, which could be the result of manual macroscopic procedures. Relative resistance changes are frequently less than 1% when used for handwriting sensing without a porous sponge below, making minor changes challenging to notice and perhaps resulting in mistakes or misclassifications. When combined with a porous sponge, sensitivity is greatly increased [161]. Green zinc/galactomannan-based hydrogels as quasi-solid aqueous electrolytes for Dye-Sensitized Solar Cells (DSSCs) present notable advantages and some limitations. The hydrogel electrolytes attain an extraordinary open-circuit voltage (Voc) of up to 750 mV, which is equivalent to high-performing organic DSSCs and much higher than the 0.4–0.6 V often seen in aqueous DSSCs. For a-DSSCs to be integrated with energy storage systems and intelligent Internet of Things (IoT) devices, this high Voc is essential. The ‘completely green’ design of these hydrogels’ manufacture involves the use of natural ingredients such as biocompatible zinc salts and galactomannan, a natural polysaccharide. Ethanol is utilized sparingly in several stages of the synthesis process, which uses environmentally benign techniques such as mild temperatures (below 40 °C) and mostly aqueous solutions. By doing this, hazardous waste products and volatile organic compounds (VOCs) are avoided. Galactomannans are inexpensive, plentiful, and environmentally benign polysaccharides. Another environmentally acceptable and sustainable substance is zinc oxide (ZnO), which is readily recyclable and disposed of after being synthesized in an environmentally favorable manner. The creation of aesthetically pleasing and semi-transparent devices is facilitated by hydrogel electrolytes. With an impressive Average Visible Transmittance (AVT) of up to 22% in the ideal circumstances, the hydroxysulphate gelator (A-series) in particular offers remarkable transparency. Applications such as Building Integrated Photovoltaics (BIPV) might benefit from this functionality. A crucial characteristic of wave-selective or semi-transparent devices is their good LUE. The LUE is more than twice as high as the xanthan gum reference device, reaching 0.35%. The hydrogel creates a quasi-solid electrolyte (QSE), which enhances the long-term stability of DSSCs and solves the leakage problem that liquid electrolytes frequently face. Compared to the reference cell’s steady 5 mA cm^−2^, the hydrogel-based DSSCs exhibit comparatively low photocurrent density (Jsc) values, never surpassing 4 mA cm^−2^. The primary cause of this reduced current density, particularly for G-A3-I, is charge diffusion restriction. According to investigations utilizing Electrochemical Impedance Spectroscopy (EIS), the diffusion resistance (Rw) increases with the quantity of galactomannan (GM) in the gels. This means that ions have a harder time diffusing through the electrolyte because of the increased viscosity. It was shown that higher GM concentrations had a negative impact on solar efficiency. Some gels, like G-A1-I and G-A2-I, exhibit steady performance right after assembly, whereas others, like G-A3-I, need to age for up to a day before reaching their optimum efficiency. This is probably because increased viscosity prevents diffusion. In comparison to the reference device, the G-AX-I systems often exhibit inferior stability; after 500 h, the G-A1-I loses around 50% of its initial efficiency [163]. For flexible zinc-manganese oxide (Zn-MnO_2_) batteries, Jiang et al. presented a highly compressible hydrogel electrolyte, a soybean protein isolate (SPI)/polyacrylamide (PAAM) composite [166]. Although this hydrogel has many benefits, especially when it comes to flexible electronics, there are several things to keep in mind. For flexible electronics, the hydrogel’s high elasticity and compressibility are essential. Even under extreme mechanical stimulation, including bending and pounding, it can sustain substantial compression (up to 96% strain) and continue to produce power steadily. The mechanical strength, toughness, and fatigue resistance of the hydrogel are greatly enhanced by the addition of SPI nanoparticles to the PAAM network. These nanoparticles help the material recuperate by serving as stress dissipation sites. Even after 10 compression cycles at 96% strain, the battery retains a sufficient specific capacity without experiencing any notable changes in appearance or electrochemical decrease. Additionally, it retains certain capacities under variable strain and exhibits stable charge/discharge properties. The hydrogel electrolyte is equivalent to liquid electrolytes and suited for Zn-MnO_2_ batteries due to its better ionic conductivity (50.8 mS cm^−1^). After 500 charge/discharge cycles, the battery made with this hydrogel has a respectable specific capacity (299.3 mA h g^−1^) and a satisfactory capacity retention rate (78.2%). By offering superior adherence to electrode materials, the hydrogel electrolyte reduces corrosion and separation and enhances cycle stability. Hydrogel electrolytes are safer than traditional liquid electrolytes since they have fewer leakage issues. Utilizing inexpensive and environmentally friendly protein nanoparticles (SPI) adds to the hydrogel electrolyte’s sustainable qualities. Although the hydrogel electrolyte exhibits good electrochemical performance, the battery’s specific capacity was marginally lower than that of a battery that used a liquid electrolyte. Due to potential moisture loss from the electrolyte over extended operation, the specific capacity may gradually drop. The SPI concentration affects the hydrogel’s compressive strength; if the mass ratio of AAm to SPI is higher than a particular threshold (0.15 mass ratio), nanoparticle aggregation may cause the strength to drop [164]. For touch panel applications, the new nanocomposite hydrogel made of poly(N,N’-dimethylacrylamide)-titanium dioxide (PDMAA-TiO_2_) offers several noteworthy benefits along with a few small disadvantages, mostly associated with operating subtleties. The hydrogel has excellent softness, stretchability, resolution, reaction time, and instantaneous functionality recovery in the event of injury. It is still functioning even when stretched to an area strain of more than 1100%. TiO_2_ nanoparticles and PDMAA chains form hydrogen bonding, which gives them exceptional fatigue resistance and self-healing properties. Because of its ability to self-heal, it can both electrically and structurally recover from damage caused by extreme strain, even after being totally severed. The touch panel has excellent operational stability, sustaining a constant touching current plateau and baseline current even after several touches. Due to its biocompatibility, the hydrogel may be used in epidermal touch panels and integrated with the human body. Because of its transparency, which is essential for display applications, visual material may be shown behind the panel. One of the desired characteristics of next-generation touch panels is the hydrogel-based touch panel’s low parasitic capacitance. In both its original and greatly expanded configurations, it offers quick and accurate touch-sensing. Accurate location detection is made possible by the fact that the amount of current generated by touch depends on the distance from the electrode. At first, the nonlinearity of the panel’s resistance caused distortion close to the edge. However, this problem was greatly mitigated by using 3D printing to optimize the panel with a four-terminal layout. Although the touch screen remains functional when stretched, the baseline current rises as a result of the hydrogel’s surface area expanding. In spite of this, the system is still able to recognize touch signals and maintain its existing touch response [166]. For soft robotic applications, the hydrogel composed of carbon nanotubes (CNTs) and poly(N-isopropylacrylamide) (PNIPAM) has a few noteworthy benefits, especially when considering the jellyfish-like miniature soft robot (JMSR). It does, however, have several drawbacks that must be fixed for wider applicability. The hydrogel’s ability to react to visible light makes it useful for soft robot remote control and untethered actuation. Operation is made simpler by avoiding direct contact with actuators. Compared to other light sources, visible light is also seen to be more ecologically beneficial for both people and marine life. When exposed to visible light, the composite hydrogel has a rapid bending angular velocity of up to 65.72°/s, particularly when the concentration of CNT is 3 mg/mL. The addition of CNTs improves the hydrogel’s absorption of visible light, which accelerates temperature rise and shape change. Additionally, CNTs increase the water’s diffusion coefficient inside the hydrogel network, which speeds up deformation. Even after 200 cycles of light-induced bending, the composite hydrogel exhibits remarkable performance stability and no discernible deterioration, demonstrating its durability for recurrent usage. The JMSR made of this hydrogel is versatile in a variety of settings, as seen by its ability to perform motion modes other than swimming, such as walking and leaping. By adjusting the light source, the JMSR can move small objects in a specific direction, demonstrating its versatility for manipulation jobs. Although the JMSR exhibits some degree of directional controllability when walking, further work is required to increase the overall controllability of its movement direction to expand its range of applications. The robot’s Reynolds number decreases with further shrinkage, making mobility more challenging. One major obstacle to future miniaturization is overcoming the difficulties posed by low Reynolds numbers [167]. Xiaoyu Luo et al. reported the 3D-printed hydrogel-based Flexible electrochromic devices (FECDs), which exhibit high optical contrast, reaching up to 54% at 360 nm [168]. They have outstanding cycle stability; even after 10,000 s, their electroactivity decreases by less than 5%. Additionally, after 5000 cycles of bending, the devices exhibit good mechanical stability, with an ideal contrast drop of less than 19%. Even under severe bending, the device’s components stay firmly joined. Direct writing 3D printing with several materials enables smooth interface integration and efficient fabrication of patterned FECDs with continuous production capability. This procedure is efficient, economical, and adaptable. Interlayer compatibility during integrated manufacturing is ensured by the constant printing settings of the electrochromic and electrolyte layer inks. In order to intuitively show biological signals, the FECDs can be affixed to the skin. Their ability to show distinct reversible colors with minimum voltage makes them appropriate for adaptive camouflage systems. Additionally, the gadgets have the potential to be smart windows, providing tinted and clear states for privacy and limiting solar radiation. Multilayer electrochromic device integrated manufacturing is a synergistic optimization process that is somewhat sophisticated. There are still difficulties in producing integrated FECDs using 3D printing, especially when it comes to choosing electrochromic inks and the intricate multi-layer flexible electrochromic device printing procedure. For multi-layer FECD printing to guarantee correct printing, form fidelity, and smooth interface integration, multi-material inks with varying viscoelasticities must be carefully coordinated. Higher air pressures and larger print tips are necessary for continuous and uninterrupted ink extrusion since low air pressures and smaller print tips might cause ink blockage and discontinuities. Carefully limiting the ideal printing ranges is necessary to avoid problems like poor form fidelity and diffusion on the substrate. Applications where visibility is crucial, such as touchpads and screens, benefit from this transparency. Hydrogels’ mechanical characteristics can be adjusted to fit certain uses. Their elastic moduli may be modified to meet the needs of the device, ranging from 1 kPa to over 100 kPa [169]. Jingsi Chen et al. created a new hydrogel by incorporating multiple hydrogen bonds and using 2-ureido-4[1H]-pyrimidinone (UPy) groups as cross-linking points to the brittle polyaniline/poly (4-styrenesulfonate) (PANI/PSS) networks. The hydrogel has a linear response (gauge factor = 3.4) to external strain (≈300%) and is elastic, self-healing, and conducts electricity well (13 S/m). This hydrogel is helpful for wearable technology since it is simple to inject and form. It can precisely track a wide range of human motions, including speaking and eating, as well as more subtle movements like bending fingers or wrists [162]. Conventional hydrogels freeze in frigid temperatures, losing important properties like conductivity and self-healing. Glycol and other antifreeze chemicals are employed; however, they must function better at low temperatures. Using zwitterionic poly (ionic liquid) (PIL), Ziyang Liu et al. developed a novel kind of hydrogel that exhibits exceptional stretchability, self-healing, and strong conductivity—even at extremely low temperatures. The PIL gel is helpful for a variety of applications and is opening the door for more sophisticated skin designs since it can be used in several sensing modes and integrated with soft robotic devices [170].

### 3.5. Application as Edible Hydrogels

Polymer networks known as edible hydrogels are safe for ingestion by humans and can swell in water. Typically, natural polymers are used to create edible hydrogels. Edible hydrogels’ exceptional ability to withstand shrinking in acidic environments is one of their most notable characteristics. This is especially crucial because many conventional hydrogels don’t work well in the acidic environment of the stomach. These natural polymer-based hydrogels provide a safe substitute for synthetic polymer-based hydrogels, which are frequently inedible and may be harmful to health. They are meant to be eaten, and by making you feel full, they may help you regulate your body weight. These hydrogels have been shown to be safe for oral administration in animal experiments, including mice and rats. According to the investigations, the test subjects behaved normally, and there were no notable negative impacts during the trial [171]. Table 6 presents a few hydrogels and their application in different sectors. There are several uses for edible hydrogels, especially in the fields of nutrition and health. The prevention and treatment of obesity is one of the main uses for edible hydrogels. By absorbing water and expanding in the digestive system, these hydrogels contribute to a sense of fullness without increasing caloric intake. They are a helpful tool for people trying to properly control their weight since this technique can lower food consumption. Additionally, edible hydrogels can be used to transport drugs or nutrients. They can efficiently supply vital nutrients because of their capacity to expand and preserve structural integrity in the gastrointestinal system, which may be very helpful for people who might find it difficult to follow conventional dietary guidelines. The hydrogels have demonstrated the ability to lower inflammatory reactions and hepatic fat buildup in obese mice, suggesting their potential to improve general health and metabolic disorders linked to obesity. Edible hydrogels have applications in the food business beyond health, including preservation and the development of low-calorie food items [172].

The edible hydrogels of sodium carboxymethylcellulose, sodium alginate, and citric acid have great potential because of their biocompatibility, biodegradability, and capacity to treat medical conditions. For broad use, issues with their mechanical stability, pH sensitivity, and long-term structural integrity in demanding physiological conditions must be properly resolved [175]. Edible hydrogels can aid in weight management by maintaining volume and elasticity within the digestive system without adding calories. Longer satiety results from this since it lowers the diet’s calorie density and increases the flexibility of food that is consumed. Calcium carbonate microparticles, mineralized pectin/poly (ethylene glycol) hydrogels, were developed by Yi Le et al. and are thought to be safe for weight control because all of their ingredients are edible [176]. During the digestive process, they have a swelling rate that is roughly double that of the original gels and may reach mechanical strengths of up to 100 times. They provide a longer-lasting and more potent sensation of fullness by maintaining a specific volume and high flexibility throughout the whole digestive tract. Previous cellulose-based hydrogels frequently had poor gastrointestinal retention and mechanical flaws, which might result in minimal fullness triggering and little physical pressure on the stomach and intestines. Certain hydrogels are sensitive to pH; in stomach fluid, the crosslinked network may shrink due to the protonation of carboxylate ions, reducing the swelling ratio and raising the chance of gastric ejection.

**Figure 13:**
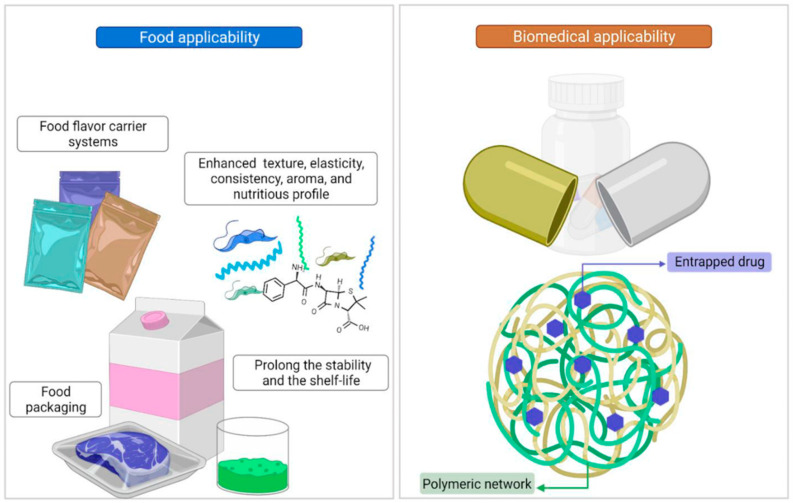
Application of edible hydrogel in food preservation and biomedical [180].

According to Alex Keller et al. the body can digest and reabsorb edible devices, even those composed of hydrogels. Because of this, they are perfect for routine GI monitoring or therapy, resolving problems such as capsule retention that arise with conventional electronic capsules that contain harmful ingredients. Hydrogels are biocompatible by nature and may be made biodegradable. In addition, they react to outside stimuli like electrical or environmental changes. Many popular hydrogel-forming polymers, such as gelatin, gellan gum, and alginate, are essential components of foods like marshmallows, ice cream, and yoghurt. Thus, hydrogels are inexpensive and widely available. The soft, rubbery texture of hydrogels when swelled in water makes it easy for them to replicate the elastic modulus of biological tissue. When optimized using techniques like post-gelation ion addition, they can also be conductive and mechanically strong. For use in edible devices, most hydrogels generally need reinforcing since they are mechanically weak. Strength is frequently compromised by conventional means of imparting conductivity, even if techniques like interpenetrating polymer networks (IPNs) might improve mechanical qualities. In general, hydrogels have very little or no electrical conductivity. Conduction-inducing techniques, including the addition of conductive polymers or fillers, frequently call for dehydration, which works against hydrogels that are intended to swell. Ionic conduction provides a solution, although it can be difficult to achieve sufficient conductivity without compromising mechanical strength, particularly if ions are introduced before gelation [178]. The potential for extending shelf-life and offering antibacterial properties makes chitosan/zinc oxide/thyme oil edible hydrogels an interesting development in food packaging. But for them to be widely used, issues with possible paradoxical antibacterial effects, sensory effects, and the requirement for more study for the best formulation and scalable production must be resolved [179]. How does hydrogel function in food/meat preservation? Because hydrogels regulate moisture levels and act as a protective barrier, hydrogels are important for maintaining the quality of meat. Hydrogels successfully stop meat products from losing moisture. This is important because dehydration can cause meat to stiffen and lose its texture. According to the study, meat might retain its quality by using hydrogels to prevent it from stiffening at higher temperatures. The use of hydrogels aids in regulating the cold meat’s temperature. To keep meat from spoiling, hydrogels can slow down the rise in core temperature that occurs when it is taken out of cold storage and placed in warmer environments. Because temperature changes might happen during storage and transit, this is very crucial. Meat’s chromaticity and lightness are preserved by hydrogels. After being exposed to greater temperatures and refrigeration, it was found that the meat covered with hydrogels changed less in color and brightness than the uncoated samples. This implies that meat’s aesthetic appeal, which is crucial for customer approval, may be preserved by hydrogels. Hydrogels have self-healing capabilities, so even if the coating is harmed (for example, by cuts), it may reattach and continue to provide protection. Maintaining protection throughout handling and storage requires this. Food preparation is made more convenient by the hydrogels’ easy removal at high temperatures. This characteristic is crucial for making sure the coating does not obstruct the cooking process. The hydrogel’s ability to absorb meat fluids without becoming too swollen might jeopardize its effectiveness. According to the study, hydrogels’ swelling rate rose as their gelatin content rose, suggesting that stability and moisture retention can be balanced [174]. Figure 13 presents the edible hydrogel-based packaging and biomedical applicability, which preserves the meat and drugs.

To lessen the carbon dioxide footprint associated with conventional packaging materials, Aris E. Giannakas et al. created a new edible hydrogel for food packaging using sodium alginate and glycerol [173]. In comparison to uncoated cheese, these edible active coatings showed a decrease in the mesophilic microbial population of almost one log_10_ unit (cfu/g) when applied to typical Greek spread cheese. Although oral protein delivery is a convenient and comfortable method for patients, it is hindered by the gastrointestinal tract’s digestive enzymes. To get around this, Meenakshi Gautam et al. created a highly bioavailable colon-specific protein delivery method. They mineralized Calcium carbonate microparticles in situ within a pectin/poly (ethylene glycol) (PEG) edible hydrogel and bovine serum albumin (BSA) used as a model drug. Protein release experiments and in vitro swelling tests demonstrated the hydrogel’s capacity to release protein precisely at the colon [177].

### 3.6. Application of Hydrogels in Thermoelectric Generators

**Figure 14:**
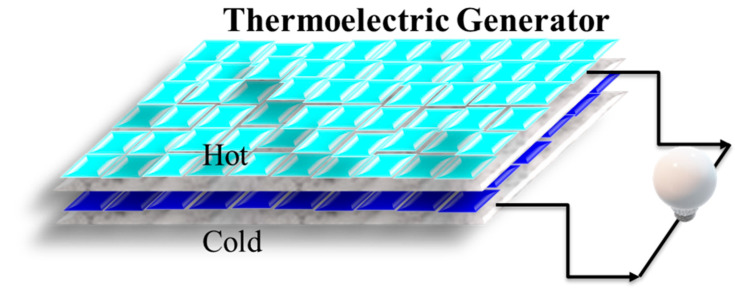
Thermoelectric generator.

The use of hydrogels in thermoelectric generators (TEGs) is growing because of their special qualities and adaptability in turning heat energy into electrical energy. In particular, they have the capacity to extract low-grade waste heat from the environment and human body, which holds great promise for wearable technology, energy storage, and applications involving human–machine interaction [181,182]. Figure 14 shows the simple module of a thermoelectric generator.

Through the Seebeck effect, hydrogels can act as electrolytes, promoting ion movement beneath a temperature gradient to produce voltage. The ion type, electrolyte content, and ionic conductivity of the hydrogel all affect how effective this procedure is. Electric current may be generated under a temperature gradient by the efficient transport of mobile ions. Hydrogels transform heat energy into electrical energy by facilitating oxidation and reduction reactions at various electrodes. These reactions are fueled by temperature gradients, where one component of the hydrogel functions as an oxidizing agent and another as a reducing agent, producing an electric current. These reactions can generate electricity in hydrogels that include redox-active molecules like ferricyanide and ferrocene [183]. When there is a temperature differential between the hot and cold sides of a TEG, hydrogels can effectively dissipate heat by absorbing and evaporating moisture from the surrounding environment. Because of their high water content and porous polymer network, they have poor thermal conductivity, which makes it possible for them to efficiently absorb and disperse heat, increasing the overall efficiency of the device [184,185]. Compared to conventional thermoelectric materials, hydrogel-based thermoelectric materials frequently show high Seebeck coefficient values, usually in the tens of mV K^−1^. Effective energy conversion depends on this high thermopower [186].

**Figure 15:**
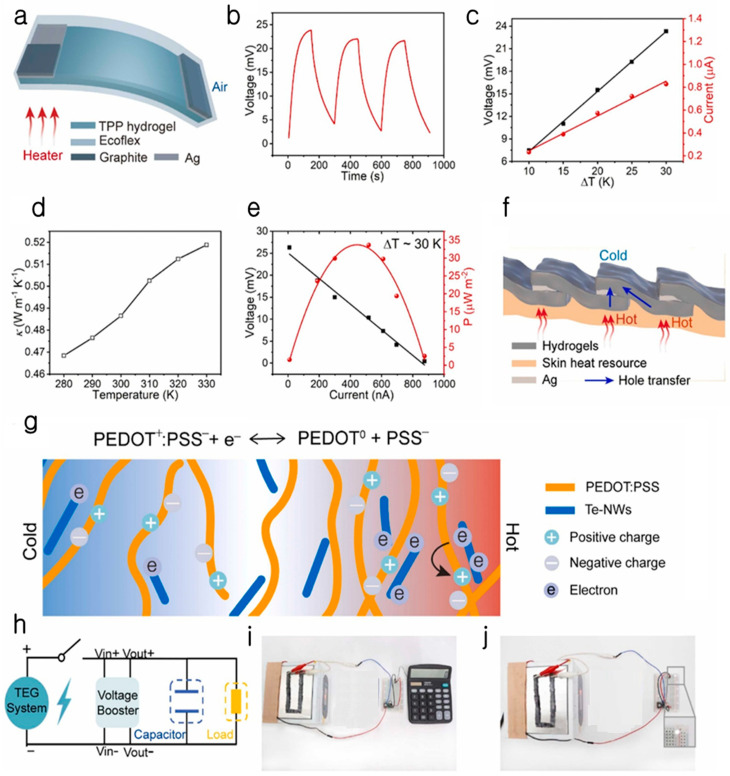
TEG’s thermoelectric characteristics, the TPP hydrogel module’s construction, and an example of how to use a thermoelectric system to power electrical devices. (**a**) Structure, (**b**) output voltages of the TEG at a temperature difference of 30 K. (**c**) Dependence of the output voltage and current on applied temperature differences. (**d**) Thermal conductivities of the hydrogels at different temperatures. (**e**) Current-voltage curve and output power of the TEG under a temperature difference of 30 K. (**f**) Construction of the hydrogel TEG module. (**g**) Thermoelectric processes and the impact of varying Te-NWs doping concentrations on the hydrogel’s thermoelectric characteristics. (**h**) Equivalent circuit for a TEG system with a power management unit. (**i**) an electronic calculator and (**j**) a white LED (Reused with permission from the reference [190]).

Hydrogels are perfect for stretchy and wearable electronics because of their superior biocompatibility and tissue-like physicochemical characteristics. Because of their innate flexibility, they can adapt to uneven surfaces, enhancing performance and interaction with heat sources [187]. Hydrogels can be made to stick to a variety of surfaces, including human skin, which makes them appropriate for wearable technology and guarantees effective heat transmission. This characteristic is very helpful for continuously gathering energy from the human body [188]. Certain hydrogels have the capacity to mend themselves, which enables them to regain their original characteristics following injury. This solves the problem of performance degradation over time and increases the longevity and dependability of hydrogel-based TEGs. In human–machine interfaces, hydrogels can function as transducers, translating tactile or temperature changes into electrical impulses that can be used for control or communication [182,189]. Electrical conductivity inside the TEG depends on the formation of a conductive network, which can be achieved by incorporating conductive polymers, like PEDOT: PSS, into hydrogels. Tellurium nanowires (Te-NWs) are one thermoelectric material that may be doped into hydrogels to produce composites with superior mechanical and thermoelectric properties. Sitao Kong et al., for instance, created tellurium-nanowire-doped PEDOT:PSS/polyvinyl alcohol (TPP) hydrogel (Figure 15), which has a huge Seebeck coefficient of 787 µV K^−1^, which is much higher than that of conventional thermoelectric materials based on conductive polymers [190]. The hotter side of the hydrogel accumulates more charge carriers (electrons or holes) than the cooler side when a temperature differential is introduced across it. An electrical current is produced when charge concentration propels the diffusion of charge carriers from the hot side to the cold side. By using the formula S = ΔV/ΔT, the Seebeck coefficient (S) measures the magnitude of the output electrical voltage (ΔV) produced by a certain temperature differential (ΔT). In this case, p-type thermoelectric materials, tellurium-nanowire-doped PEDOT: PSS/polyvinyl alcohol (TPP) hydrogels, collect positive charges (holes) on the hot side. When PEDOT:PSS occurs, the hot side’s PEDOT^+^ is easily converted back to PEDOT by absorbing electrons (a reduction process). PEDOT^+^ and PSS can have their electrostatic attraction weakened by component interactions, such as the hydrogen bonding between PSS (from PEDOT:PSS) and PVA. This weakening makes it easier for PEDOT^+^ to undergo a reduction process, which significantly raises the Seebeck coefficient. Low thermal conductivity values, such as 0.468 W m^−1^ K^−1^, are displayed by these hydrogels. Enhancing phonon scattering and decreasing lattice thermal conductivity are two benefits of the porous composite construction [190]. Hydrogel is a significant material for harvesting low-grade heat energy from sources like the human body because of its exceptional thermoelectric qualities, which include low thermal conductivity and high Seebeck coefficients, along with its great stretchability. The application of different hydrogels in thermoelectric applications is shown in Table 7.

Jing Liu et al. reported hydrogels, based on poly(3,4-ethylenedioxythiophene): poly (styrene sulfonate) (PEDOT:PSS) which offer unique characteristics for wearable thermoelectric (TE) energy harvesting applications [192]. PEDOT:PSS is a promising organic electron material that is ideal for wearable energy harvesting devices since it is flexible and non-toxic, outperforming several inorganic materials. One major benefit of using PEDOT:PSS-based hydrogels to produce wearable technology is their ease of processing. Because of their distinctive structural characteristics, PEDOT:PSS-based hydrogel fibers are appealing for uses such as energy storage and stress sensors. The gelation process of PEDOT:PSS allows for the creation of any shapes, which is advantageous for a variety of wearable forms. The gelation process may greatly increase the electrical conductivity of PEDOT:PSS hydrogel fibers, particularly when post-treated with organic solvents like ethylene glycol (EG) and dimethyl sulfoxide (DMSO). For example, one study found that PEDOT:PSS fibers had an electrical conductivity of 58.6 S·cm^−1^, three orders of magnitude greater than hydrogel fibers that had been previously reported. This enhancement, which results in a more continuous electrically conductive grid, is ascribed to the elimination of insulation from PSS chains. Despite the special qualities of hydrogels, it has been determined that the electrical conductivity of PEDOT:PSS fibers in existing hydrogels is inadequate for efficient wearable TE energy harvesting. This calls for more conductivity enhancements. When discharged into deionized water or onto a glass substrate, as-formed PEDOT:PSS hydrogel fibers may be brittle and find it challenging to retain their whole fiber structure. Their practical use in flexible wearable technology may be hampered by their brittleness. Early techniques for creating hydrogels frequently produced a porous structure as a result of freeze-drying. This loose structure has a detrimental effect on electrical conductivity and is associated with a low solid concentration of PEDOT:PSS in solution. In order to get around this, methods like condensing the initial PEDOT: PSS volume are used to increase the mass concentration and prevent needless porosity [192]. Hydrogels, especially those based on polyvinyl alcohol (PVA) and gelatin methacrylate (GelMA), have some disadvantages as well as clear benefits when used in thermoelectric generators (TEGs). Hydrogels can create ionic thermoelectric (i-TE) materials, which are distinguished by their poor heat conductivity and high thermopower (S). These characteristics improve their thermoelectric performance, which makes them effective in producing power from low-quality waste heat. For wearable technology that must adapt to body motions and reduce the chance of breakage, hydrogels provide advantageous mechanical qualities, including flexibility. Small-angle X-ray scattering (SAXS) shows that the microstructure of hydrogels has a role in their mechanical resilience. Wearable TEG applications can benefit from the exceptional toughness of the GP5 hydrogel, which is a particular blend of GelMA and PVA. Hydrogel-based i-TE materials provide a more sustainable and environmentally acceptable alternative to some inorganic thermoelectric materials that have significant toxicity, which is crucial for wearable human waste-heat harvesting devices. Because of its speedy photo-crosslinking properties, gelatin methacrylate (GelMA) may be used in bioprinting and to speed up and stabilise the gelation process required for TEG manufacturing. Continuous power production and thermal voltage stability depend on the hydrogel matrix’s high ionic conductivity (e.g., ≈108 mS cm^−1^) and effective charge transfer when infiltrated with redox couples such as Fe(CN)_6_^3-/4-^. The quality of the contact between the hydrogel and the electrode is enhanced when TEGs containing hydrogels are created via 3D printing. Additionally, this makes the process of fabricating TEGs easier. High crosslinking density in GelMA with a high degree of substitution (DS) can produce a hydrogel that is inherently brittle and stiff. To create a sufficiently strong and flexible material, other polymers, such as PVA, must be added. Although PVA increases flexibility, too many PVA segments in the polymer matrix can cause the hydrogel to become uneven, as the GP3 hydrogel demonstrates. If covalently cross-linked GelMA does not completely cover the PVA segments, the mechanical characteristics may drastically deteriorate [193]. 

## 4. Scope of Improvement and Future Prospect

Although hydrogels have advanced significantly in recent years, there are still a number of areas that might be improved and opportunities for the future to fulfil a variety of needs in daily life. Currently, hydrogels have a drawback, including fatigue and cracking when subjected to heavy loads in harsh environments. Their network structure and chemical makeup are the causes of these problems. One intriguing way to get around these restrictions and improve the hydrogel’s resilience and longevity is to form double crosslinked networks. Low conductivity might present an unexpected difficulty when using hydrogels in real-time applications. Because of their increased surface area and synergistic interactions, conducting materials like nanofillers can be used to reinforce hydrogels, improving their conductivity, mechanical strength, and optoelectronic characteristics. To increase conductivity, nanofillers such as conducting polymers, metal oxide/sulfide/phosphate, graphene, carbon nanotubes, and carbon dots can be added. Hydrogels and electroactive materials need to be balanced carefully for flexible electrochemical devices. Additionally, nanofillers can enhance connection, ionic transport, and cycle stability in the porous networks of hydrogel electrolytes. Specialized hydrogels are perfect for a variety of uses, such as medication administration, electronics, sensing, cell imaging, and tissue engineering. Further applications of highly conducting hybrid hydrogels include 3D printing, tissue creation, soft robotics, and artificial skin. Hydrogels that conduct ions are very useful for limited bioelectronics. New and exciting avenues for the large-scale development of ionic hydrogels include advanced manufacturing techniques like inkjet printing and three-dimensional printing. Solar cells and thermoelectric generators are the most recent, prospective, and pioneering research fields with many limitations. Incorporation of conductive hydrogels in these fields can extend the performance and efficiency. Radiation methods are mostly used for hydrogel preparation, giving pure hydrogels that are convenient to use. The improved characteristics of radiation-grafted copolymers (RGCs) make them highly promising for drug delivery applications as well as separation and purification. Nevertheless, a few obstacles must be overcome to reach their full potential and increase their commercialization.

When subjected to ionizing radiation during preparation, some backbone polymers—particularly natural ones like starch, carrageenan, and chitosan, as well as synthetic ones like PP and PTFE—degrade significantly. This deterioration shortens the functional copolymers’ lifetime and decreases their mechanical and chemical stability, rendering them unsuitable for real-world uses. Although crosslinking might lessen this deterioration, it complicates and increases the cost of the production process and needs careful optimization to preserve the required flexibility and swelling. RGC performance can be adversely affected by the unequal and inhomogeneous distribution of side-chain grafts, which carry the functional groups that cause separation, especially in membrane forms. In electrochemical cells, for example, membranes with irregular functionalized grafts may result in high internal resistance, which might lower power output or increase consumption. Despite their superior laboratory performance, RGC materials’ relatively expensive cost has prevented their commercial acceptance. The price of irradiation services and beginning materials is partially to blame for this. Routine use and the investigation of novel materials and methods are restricted by the dependence on nuclear research institutes and manufacturers for irradiation facilities, especially Co-60 sources that need lengthy processing periods for bulk grafting. Even while electron accelerators speed up processing, there are still issues with their availability. Future research should concentrate on developing RGCs that are more resilient to separation issues than traditional materials. Creating functional polymers with enhanced permselectivity, high conductivity, mechanical integrity, chemical stability, fouling resistance, and heat resistance is part of this. Using more effective grafting processes, such as emulsion systems, which may drastically reduce irradiation doses and monomer use, is one way to cut manufacturing costs. Costs can also be decreased by utilizing inexpensive backbone polymers, such as waste fibers, non-woven textiles, and natural polymers, particularly when exposed to low radiation doses. To increase their use in both business and research, more affordable, potent electron accelerators with the right safety features must be developed. It is crucial to choose the right functional groups and ligands to increase the selectivity and adsorption capability of ion exchangers and chelating agents. Product development from laboratories to industrial sizes may be facilitated by seamlessly combining RGC technology with other disciplines such as chemical, mechanical, and electrical engineering. This calls for a multidisciplinary strategy to guarantee ongoing cost-effectiveness and repeatability.

## 5. Conclusions

The review elaborately explains the fundamental concept of hydrogel preparation and mechanisms in different fields. Recent research on hydrogels for versatile applications has been critically reviewed to depict the advantages and drawbacks. Physical, chemical, and hybrid bonding are among the bonding types used in hydrogel synthesis. Solution casting, solution mixing, bulk polymerization, radiation techniques, free radical processes, and the development of interpenetrating networks are examples of common pathways. Important properties of synthesized hydrogels include swellability, biocompatibility, biodegradability, mechanical strength, and stimuli sensitivity. Their utility in biological and electrochemical applications depends on these qualities. Innovative functional hydrogels play vital roles in many fields of application, especially in water purification, biomedical engineering, agriculture, electronics, and food preservation and packaging. In each individual application field, hydrogel uses its specific characteristic. Hydrogels can respond to temperature, pH, ionic strength, electric fields, and biological molecules, which makes them valuable for smart applications. Their properties, particularly their conductivity, self-healing capabilities, and mechanical robustness, are being improved by ongoing research, opening the door for increasingly sophisticated, flexible, and intelligent electronic systems. After all, this review will build fundamental knowledge of hydrogel chemistry.

## Figures and Tables

**Table 3:** Application of different hydrogels in agriculture.

Hydrogel Composition	Properties	Field of Application	Function	Reference
Lignin/Konjaku flour/Sodium Alginat (L/KJ/SA)	Hydrogels influence chemical and physical properties of soil, such as maximum water holding capacity	Water supply in soil	Hydrogel degrades by 20% in 120 days	[116]
Acrylic acid/acrylamide/2-acrylamido-2-methylpropane sulfonic acid P(AA-AM-AMPS)	Hydrogel is economically injectable superabsorbent.	Water retention	Maximum swelling 509 g/g.	[117]
Acrylic acid/Acrylamide	Superabsorbent	Water supply to plant root	In 15 days, plant roots and leaves are positively impacted by soil containing 0.2% SAPs	[118]
Sodium alginate/chitosan-based *Perilla frutescens* L. essential oil	Hydrogel can reduce the number of microorganisms	Strawberries preservation	The strawberries’ ability to be preserved was greatly enhanced by hydrogel beads. The look was satisfactory after five days of storage.	[119]
Poly(lactic acid)/cellulose	Hydrogel can act as water and fertilizer reservoir in agricultural	Water and fertilizer reservoir in agricultural	The hydrogel substance is useful for altering the soil’s capacity to retain water, especially when it comes to red soil.	[120]
Carboxymethylcellulose/sodium salt/hydroxy ethyl cellulose	Hydrogel is superabsorbent and biodegradable	Plant growth	When selected plants were cultivated in changed soil as opposed to unmodified soil, crucial characteristics including photosynthetic capacity (16.74%), plant growth rate (3.77%), and seedling viability (4.51%) increased.	[121]
Water hyacinth cellulose-graft-poly (ammonium acrylate-co-acrylic acid) (cellulose-g-PAM-co-AA)	Hydrogel displays biodegradability	Water retention	Hydrogel has the capacity to absorb and hold onto water in the soil.	[122]
Succinate-modified potato starch	Hydrogel is biodegradable	Corn seed coating to enhance early development	The hydrogel coating’s contribution to the plant’s water needs diminishes during seed emergence and root growth.	[123]
Poly (vinyl pyrrolidone)	Hydrogel is multifunctional and superabsorbent	Water retention in plants	Water conservation, regulated nutrient delivery, and enhanced plant performance during drought stress are all made possible by the hydrogel.	[124]

**Table 5:** Application of hydrogels in different electronic devices.

Hydrogel Composition	Properties	Field of Application	Function	Reference
Cellulose	Hydrogel shows tunable swelling and biocompatibility.	Sensor	Applied in strain sensor. The gauge factor within the range of 90% to 600% is 1.7.	[160]
Carrageenan/PDMAPS, MXene/Tannic acid (CMM).	Hydrogel exhibits enhanced mechanical strain.	Sensor	Exhibits swift responsive time (220 ms/230 ms).	[161]
Polyaniline/poly (4- styrene sulfonate	Hydrogel exhibits stretchability and rapid self-healing capability.	Flexible electronics	Complete self-healing within 30 s upon damage	[162]
Zinc/ galactomannan	Hydrogel is highly efficient and sustainable	Quasi solid aqueous dye sensitized solar cells	Display an unprecedent open circuit voltage (VOC) as high as 750 mV.	[163]
Soybean protein isolate nanoparticles (SPI) cross-linked polyacrylamide	Hydrogel is highly compressible	As electrolyte for flexible Zn-MnO_2_ battery	Exhibits specific capacity (299.3 mA h g^−1^) and desirable capacity retention rate (78.2%) after 500 charge/discharge cycles.	[164]
Poly(*N*,*N*′-dimethylacrylamide)/silica	Ultra-stretchable and sensitive	Soft touch panel	Ultra-high stretchability > 3800%.	[165]
Poly(N,N-dimethylacrylamide)–titanium dioxide nanocomposite hydrogel.	Exhibits high stretchability and instant functionality Self-healing.	Semiconductive touch panel.	The panel is stretched to more than 1100% areal strain.	[166]
Poly(N-isopropylacrylamide)/carbon nanotubes	Thermal-responsive	Soft Robot	The robot can move at a maximum speed of 3.37 mm/s.	[167]
Viologen/polyvinyl alcohol	3D-printable	Wearable Displays	Shows high optical contrast (up to 54% at 360 nm), nice cycling stability (less than 5% electroactivity reduction after 10,000 s)	[168]

**Table 6:** Application of different edible hydrogels.

Hydrogel Composition	Properties	Field of Application	Performance/Efficiency	Reference
Alginate/glycerol/thymol/halloysite	Biocompatible	Edible coatings for dairy products.	Reduce the mesophilic microbial population over one log10 unit (cfu/g) compared to uncoated cheese	[173]
Sodium carboxymethylcellulose/Citric acid/hyaluronic acid	Highly expandable	Obesity treatment	Preventing and treating obesity	[172]
Gelatin/potassium ion-mediated k-carrageenan	Capable of self-healing and anti-freezing	Fresh meat preservation	Protecting meat with a E value of 50.4 and 17.53 N of shear force	[174]
Chitosan-polyacrylate-curdlan hydrogel	Shows extremely high swelling	Weight Control	Potentially reducing food intake and aiding in weight control.	[175]
Sodium carboxymethylcellulose/citric acid/sodium alginate	Mechanically strong	Obesity management	Potential for applications in weight loss interventions and long-term drug delivery	[176]
Calcium carbonate microparticles-mineralized pectin/poly (ethylene glycol)	Hydrogel shows dynamic nature	Colon specific protein delivery	Potential drug carrier in releasing protein at the colon site	[177]
Gellan gum/gelatin	Conductive and edible	Edible electrodes	Potential for detecting digestive pressure abnormalities such as intestinal motility disorders	[178]
Chitosan/zinc oxide/thyme oil	Antimicrobial hydrogel	Food coating	Extend shelf-life tofu sausages	[179]

**Table 7:** Different hydrogels in thermoelectric application.

Hydrogel Composition	Properties	Field of application	Function	Reference
Carboxylated cellulose nanofibers, Ag_4_Te, Cu_1.5_Te	Printable	Flexible thermoelectric (TE) devices	High power density of 1.278 W m^−2^ under a temperature difference of 50 K.	[191]
PEDOT:PSS	Highly Conductive	Wearable Thermoelectric Energy Harvesting	A power density of 481.2 μW·cm^−2^ with a temperature difference of ~60 K	[192]
Gelatin Methacrylate/polyvinyl alcohol	3D Printable	Wearable Thermoelectric Generators	A power density of 4.0 mWm^−2^ under a temperature gradient of 2.5 K	[193]
Poly (vinyl alcohol), ferric/ferrous chloride	Good mechanical property	Wearable Thermocells	A power density of 90 nW under a temperature gradient of 20 K	[182]
Graphene oxide/modified acrylamide ionogel	Self-healable	Ionogel Thermoelectric Films	A power density of 1.32 mW cm^−2^ under a temperature gradient of 20 K	[183]
polyacrylamide/sodium carboxymethyl cellulose	Bi-functional	Ionic Thermoelectric Supercapacitor	Storage capability (~1.3 mC, DT 10 K)	[188]
PEDOT: PSS/polyvinyl alcohol/Tellurium-nanowire	Highly stretchable	Low-grade heat energy harvesting	Can achieve a voltage output of 138 mV when it is applied on the human arm	[190]
Bi_0_._5_Sb_1_._5_Te_3_/PEDOT: PSS	Flexible	Thermoelectric film and device	generating thermovoltage of ~7.7 mV with the human wrist as the thermal source	[185]

## Data Availability

Not applicable.

## References

[1] Priya A.S., Premanand R., Ragupathi I., Bhaviripudi V.R., Aepuru R., Kannan K., Shanmugaraj K. (2024). Comprehensive Review of Hydrogel Synthesis, Characterization, and Emerging Applications. J. Compos. Sci..

[2] Sikdar P., Uddin M.M., Dip T.M., Islam S., Hoque M.S., Dhar A.K., Wu S. (2021). Recent Advances in the Synthesis of Smart Hydrogels. Mater. Adv..

[3] Sahu K., Chakma S. (2024). Recent Trends on Hydrogel Development and Sustainable Applications: A Bibliometric Analysis and Concise Review. Polym. Bull..

[4] Castro-Dominguez B., Gröls J.R., Alkandari S., Perge L., Sierra-Avila C., Ramirez H.Z., de Lima Fontes M., Yamada C., Lazarini S.C., Silva J.M. (2025). Biopolymers and Biocomposites: A Comprehensive Review of Feedstocks, Functionalities, and Advanced Manufacturing Techniques for Sustainable Applications.

[5] Kogje M., Satdive A., Mestry S., Mhaske S.T. (2025). Biopolymers: A Comprehensive Review of Sustainability, Environmental Impact, and Lifecycle Analysis.

[6] Pocurull E., Fontanals N., Calull M., Aguilar C. (2019). Environmental Applications. Liq. Extr..

[7] Wampler F.M. (1988). Formation of Diacrylic Acid During Acrylic Acid Storage. Plant/Oper. Prog..

[8] Sadeghi M., Hosseinzadeh H. (2010). Synthesis and Super-Swelling Behavior of a Novel Low Salt-Sensitive Protein-Based Superabsorbent Hydrogel: Collagen-g-Poly(AMPS). Turk. J. Chem..

[9] Ahmed E.M. (2015). Hydrogel: Preparation, Characterization, and Applications: A Review. J. Adv. Res..

[10] Nasef M.M., Güven O. (2012). Radiation-Grafted Copolymers for Separation and Purification Purposes: Status, Challenges and Future Directions. Prog. Polym. Sci..

[11] Badsha M.A.H., Khan M., Wu B., Kumar A., Lo I.M.C. (2021). Role of Surface Functional Groups of Hydrogels in Metal Adsorption: From Performance to Mechanism. J. Hazard. Mater..

[12] Bhuyan M.M., Jophous M., Jeong J.H. (2022). Synthesis and Characterization of Gamma Radiation-Induced (3-Acrylamidopropyl) Trimethylammonium Chloride-Acrylic Acid Functional Superabsorbent Hydrogel. Polym. Bull..

[13] Bashir S., Hina M., Iqbal J., Rajpar A.H., Mujtaba M.A., Alghamdi N.A., Wageh S., Ramesh K., Ramesh S. (2020). Fundamental Concepts of Hydrogels: Synthesis, Properties, and Their Applications. Polymers.

[14] Ho T.C., Chang C.C., Chan H.P., Chung T.W., Shu C.W., Chuang K.P., Duh T.H., Yang M.H., Tyan Y.C. (2022). Hydrogels: Properties and Applications in Biomedicine. Molecules.

[15] Majcher M.J., Hoare T., Jafar Mazumder M., Sheardown H., Al-Ahmed A. (2019). Applications of Hydrogels. Functional Biopolymers. Polymers and Polymeric Composites: A Reference Series.

[16] Paudel A., Crum A.N., Wang Y. (2024). A Full Metal-Free Flexible Ammonium-Ion Battery with Biodegradable Hydrogel Electrolyte. J. Mater. Chem. A.

[17] Zhao R., Yan X., Lin H., Zhao Z., Song S. (2025). Mechanical Tough, Stretchable, and Adhesive PEDOT:PSS-Based Hydrogel Flexible Electronics towards Multi-Modal Wearable Application. Chem. Eng. J..

[18] Ullah F., Othman M.B.H., Javed F., Ahmad Z., Akil H.M. (2015). Classification, Processing and Application of Hydrogels: A Review. Mater. Sci. Eng. C.

[19] Han Q., Li J., Candiloro Z.P.J., Cai X., Su Y., Li H., Tran N., Zhai J., Martin A.V., Drummond C.J. (2025). Alginate-Ionic Liquid Injectable Hydrogels Supporting Protein Crystallization. Int. J. Biol. Macromol..

[20] Gulrez S.K., Al-Assaf S., Phillips G.O. (2011). Hydrogels: Methods of Preparation, Characterisation and Applications. Progress in Molecular and Environmental Bioengineering—From Analysis and Modeling to Technology Applications.

[21] Cook K.R., Head D., Dougan L. (2023). Modelling Network Formation in Folded Protein Hydrogels by Cluster Aggregation Kinetics. Soft Matter.

[22] Liu Y., Zhao W.J., Li J.L., Wang R.Y. (2015). Distinct Kinetics of Molecular Gelation in a Confined Space and Its Relation to the Structure and Property of Thin Gel Films. Phys. Chem. Chem. Phys..

[23] Behera S., Mahanwar P.A. (2020). Superabsorbent Polymers in Agriculture and Other Applications: A Review. Polym. Technol. Mater..

[24] Burkert S., Schmidt T., Gohs U., Dorschner H., Arndt K.F. (2007). Cross-Linking of Poly(N-Vinyl Pyrrolidone) Films by Electron Beam Irradiation. Radiat. Phys. Chem..

[25] Mishra A., Omoyeni T., Singh P.K., Anandakumar S., Tiwari A. (2024). Trends in Sustainable Chitosan-Based Hydrogel Technology for Circular Biomedical Engineering: A Review. Int. J. Biol. Macromol..

[26] Schmidt R.F., Lutzki J., Dalgliesh R., Prévost S., Gradzielski M. (2025). PH-Responsive Rheology and Structure of Poly(Ethylene Oxide)-Poly(Methacrylic Acid) Interpolymer Complexes. Macromolecules.

[27] Hameed H., Faheem S., Paiva-Santos A.C., Sarwar H.S., Jamshaid M. (2024). A Comprehensive Review of Hydrogel-Based Drug Delivery Systems: Classification, Properties, Recent Trends, and Applications. AAPS PharmSciTech.

[28] Zu Y., Zhang Y., Zhao X., Shan C., Zu S., Wang K., Li Y., Ge Y. (2012). Preparation and Characterization of Chitosan-Polyvinyl Alcohol Blend Hydrogels for the Controlled Release of Nano-Insulin. Int. J. Biol. Macromol..

[29] Yammine P., El Safadi A., Kassab R., El-Nakat H., Obeid P.J., Nasr Z., Tannous T., Sari-Chmayssem N., Mansour A., Chmayssem A. (2025). Types of Crosslinkers and Their Applications in Biomaterials and Biomembranes. Chemistry.

[30] Li Y., Zhou T., Yu Z., Wang F., Shi D., Ni Z., Chen M. (2020). Effects of Surfactant and Ionic Concentration on Properties of Dual Physical Crosslinking Self-Healing Hydrogels by Hydrophobic Association and Ionic Interactions. New J. Chem..

[31] Mathew A.P. (2013). Interpenetrating Polymer Networks: Processing, Properties and Applications. Adv. Elastomers.

[32] Haque S.N., Bhuyan M.M., Jeong J.-H. (2024). Radiation-Induced Hydrogel for Water Treatment. Gels.

[33] Hu W., Wang Z., Xiao Y., Zhang S., Wang J. (2019). Advances in Crosslinking Strategies of Biomedical Hydrogels. Biomater. Sci..

[34] Naranjo-alcazar R., Bendix S., Groth T. (2023). Injectable Systems for Bioprinting and Tissue Engineering. Gels.

[35] Parhi R. (2017). Cross-Linked Hydrogel for Pharmaceutical Applications: A Review. Adv. Pharm. Bull..

[36] Ricciardi R., Gaillet C., Ducouret G., Lafuma F., Lauprêtre F. (2003). Investigation of the Relationships between the Chain Organization and Rheological Properties of Atactic Poly(Vinyl Alcohol) Hydrogels. Polymer.

[37] Bhattarai N., Gunn J., Zhang M. (2010). Chitosan-Based Hydrogels for Controlled, Localized Drug Delivery. Adv. Drug Deliv. Rev..

[38] Meenakshi, Ahuja M. (2015). Metronidazole Loaded Carboxymethyl Tamarind Kernel Polysaccharide-Polyvinyl Alcohol Cryogels: Preparation and Characterization. Int. J. Biol. Macromol..

[39] Devi V. K. A., Shyam R., Palaniappan A., Jaiswal A.K., Oh T.-H., Nathanael A.J. (2021). Self-Healing Hydrogels: Preparation, Mechanism and Advancement in Biomedical Applications. Polymers.

[40] Comaposada J., Gou P., Marcos B., Arnau J. (2015). Physical Properties of Sodium Alginate Solutions and Edible Wet Calcium Alginate Coatings. LWT—Food Sci. Technol..

[41] Dodero A., Pianella L., Vicini S., Alloisio M., Ottonelli M., Castellano M. (2019). Alginate-Based Hydrogels Prepared via Ionic Gelation: An Experimental Design Approach to Predict the Crosslinking Degree. Eur. Polym. J..

[42] Afonin A.V., Rusinska-Roszak D. (2024). Quantification of Hydrogen Bond Energy Based on Equations Using Spectroscopic, Structural, QTAIM-Based, and NBO-Based Descriptors Which Calibrated by the Molecular Tailoring Approach. J. Mol. Model..

[43] Chen S., Wang Z. (2023). Using Implicit-Solvent Potentials to Extract Water Contributions to Enthalpy—Entropy Compensation in Biomolecular Associations. J. Phys. Chem. B.

[44] Kinoshita M., Hayashi T. (2017). Unified Elucidation of the Entropy-Driven and -Opposed Hydrophobic Effects. Phys. Chem. Chem. Phys..

[45] Tabor R.F., Grieser F., Dagastine R.R., Chan D.Y.C. (2014). The Hydrophobic Force: Measurements and Methods. Phys. Chem. Chem. Phys..

[46] Naito H., Sumi T., Koga K. (2024). The Nature of the Hydrophobic Interaction Varies as the Solute Size Increases from Methane’s to C60′s. J. Chem. Phys..

[47] Nichifor M. (2023). Role of Hydrophobic Associations in Self-Healing Hydrogels Based on Amphiphilic Polysaccharides. Polymers.

[48] Makowski M. (2020). In Fl Uence of Ionic Strength on Hydrophobic Interactions in Water: Dependence on Solute Size and Shape. J. Phys. Chem. B.

[49] Wilding N.B., Turci F. (2025). Origin of the Inverse Temperature Dependence of Hydrophobic Attraction. Phys. Rev. Res..

[50] Liu Y.-Z., Chen Y.-N., Sun Q. (2024). The Dependence of Hydrophobic Interactions on the Shape of Solute Surface. Molecules.

[51] Akiyoshi K., Kang E.C., Kurumada S., Sunamoto J., Principi T., Winnik F.M. (2000). Controlled Association of Amphiphilic Polymers in Water: Thermosensitive Nanoparticles Formed by Self-Assembly of Hydrophobically Modified Pullulans and Poly(N-Isopropylacrylamides). Macromolecules.

[52] Zhang C., Demco D.E., Rimal R., Köhler J., Vinokur R., Singh S., Tenbusch J., Möller M. (2023). Hydrophobic Interaction within Sparsely N-Alkylated Poly(N-Vinylacetamide)s Enables Versatile Formation of Reversible Hydrogels. Macromolecules.

[53] Valencia G.A., Zare E.N., Makvandi P., Gutiérrez T.J. (2019). Self-Assembled Carbohydrate Polymers for Food Applications: A Review. Compr. Rev. Food Sci. Food Saf..

[54] Li Y.K., Guo C.G., Wang L., Xu Y., Liu C.Y., Wang C.Q. (2014). A Self-Healing and Multi-Responsive Hydrogel Based on Biodegradable Ferrocene-Modified Chitosan. RSC Adv..

[55] Duan J., Jiang J., Li J., Liu L., Li Y., Guan C. (2015). The Preparation of a Highly Stretchable Cellulose Nanowhisker Nanocomposite Hydrogel. J. Nanomater..

[56] Appel E.A., Tibbitt M.W., Webber M.J., Mattix B.A., Veiseh O., Langer R. (2015). Self-Assembled Hydrogels Utilizing Polymer-Nanoparticle Interactions. Nat. Commun..

[57] Ashfaq A., Clochard M.C., Coqueret X., Dispenza C., Driscoll M.S., Ulański P., Al-Sheikhly M. (2020). Polymerization Reactions and Modifications of Polymers by Ionizing Radiation. Polymers.

[58] Espinosa-rodriguez A., Sanchez-parcerisa D., Ib P., Antonio J., Mazal A., Fraile L.M., Ud M. (2022). Radical Production with Pulsed Beams: Understanding the Transition to FLASH. Int. J. Mol. Sci..

[59] Belkhir K., Riquet G., Becquart F. (2022). Review Article Polymer Processing under Microwaves. Adv. Polym. Technol..

[60] Taimur S., Rehman S., Ellahi M., Rizwan S. (2024). Materials Advances Polyamidoxime Brushes as an Efficient Catalytic System for Nitroarene Reduction. Mater. Adv..

[61] Mantha S., Pillai S., Khayambashi P., Upadhyay A., Zhang Y. (2019). Smart Hydrogels in Tissue Engineering and Regenerative Medicine. Materials.

[62] More A.P., Chapekar S. (2023). Irradiation Assisted Synthesis of Hydrogel: A Review.

[63] Krstić J., Spasojević J., Radosavljević A., Perić-Grujić A., D Strok Signurić M., Kačarević-Popović Z., Popović S.S.S. (2014). In Vitro Silver Ion Release Kinetics from Nanosilver/Poly(Vinyl Alcohol) Hydrogels Synthesized by Gamma Irradiation. J. Appl. Polym. Sci..

[64] Šrámková P., Kučka J., Kroneková Z., Lobaz V., Šlouf M., Mičušík M., Šepitka J., Kleinová A., Chorvát D., Mateášik A. (2023). Electron Beam Irradiation as a Straightforward Way to Produce Tailorable Non-Biofouling Poly(2-Methyl-2-Oxazoline) Hydrogel Layers on Different Substrates. Appl. Surf. Sci..

[65] Rosiak J.M., Ulański P. (1999). Synthesis of Hydrogels by Irradiation of Polymers in Aqueous Solution. Radiat. Phys. Chem..

[66] Krishna Murthy S., Veerabhadraiah Basavaraj B., Srinivasan B. (2023). Microwave Assisted Vanillin Crosslinked Chitosan/Polycarbophil Superporous Hydrogels for Biomedical Application: Optimization and Characterization. Mater. Today Proc..

[67] Nizam El-Din H.M., El-Naggar A.W.M. (2014). Radiation Synthesis of Magnetic Sensitive Ferrogels from Poly(Ethylene Glycol) and Methacrylic Acid as Drug Delivery Matrices for Vitamin B12. Des. Monomers Polym..

[68] Călina I., Demeter M., Scărișoreanu A., Abbas A., Raza M.A. (2025). Role of Ionizing Radiation Techniques in Polymeric Hydrogel Synthesis for Tissue Engineering Applications. Gels.

[69] Jeong J.O., Park J.S., Kim E.J., Jeong S.I., Lee J.Y., Lim Y.M. (2020). Preparation of Radiation Cross-Linked Poly(Acrylic Acid) Hydrogel Containing Metronidazole with Enhanced Antibacterial Activity. Int. J. Mol. Sci..

[70] Bhuyan M.M., Jeong J.H. (2023). Synthesis and Characterization of Gamma Radiation Induced Diallyldimethylammonium Chloride-Acrylic Acid-(3-Acrylamidopropyl) Trimethylammonium Chloride Superabsorbent Hydrogel. Gels.

[71] Licea-Claveríe A., Salgado-Rodríguez R., Lugo-Medina E., Arndt K.F. (2008). Incorporation of Acid Polymethacrylates into Temperature Sensitive Hydrogels Using Electron Beam Irradiation. Polym. Bull..

[72] Bayat M., Nasri S. (2019). Injectable Microgel-Hydrogel Composites “Plum Pudding Gels”: New System for Prolonged Drug Delivery.

[73] Mihoub M., Hamri S., Bouchaour T., Popa M., Popa D.M., Bedjaoui Alachaher L., Hamcerencu M. (2022). An Interpenetrating Polymer Network Hydrogel Based on Cellulose, Applied to Remove Colorant Traces from the Water Medium: Electrostatic Interactions Analysis. Polymers.

[74] Jaramillo-Quiceno N., Duque Carmona A.S., Serna J.S., Carmona D.M., Torres-Taborda M., Hincapié-Llanos G.A., Marín J.F.S., Álvarez-López C. (2024). Enhancing Soil Water Retention and Plant Growth with Thermal Crosslinked Silk Sericin-Based Hydrogel. J. Environ. Chem. Eng..

[75] Ekenseair A.K., Boere K.W.M., Tzouanas S.N., Vo T.N., Kasper F.K., Mikos A.G. (2012). Structure-Property Evaluation of Thermally and Chemically Gelling Injectable Hydrogels for Tissue Engineering. Biomacromolecules.

[76] Jafari A., Hassanajili S., Azarpira N., Bagher Karimi M., Geramizadeh B. (2019). Development of Thermal-Crosslinkable Chitosan/Maleic Terminated Polyethylene Glycol Hydrogels for Full Thickness Wound Healing: In Vitro and in Vivo Evaluation. Eur. Polym. J..

[77] Moreira Teixeira L.S., Feijen J., van Blitterswijk C.A., Dijkstra P.J., Karperien M. (2012). Enzyme-Catalyzed Crosslinkable Hydrogels: Emerging Strategies for Tissue Engineering. Biomaterials.

[78] Qi Y., Wang F., Liu J., Wang C., Liu Y. (2025). Enzyme-Mediated Hydrogelation for Biomedical Applications: A Review. Int. J. Biol. Macromol..

[79] Zhu C., Lei H., Fan D., Duan Z., Li X., Li Y., Cao J., Wang S., Yu Y. (2018). Novel Enzymatic Crosslinked Hydrogels That Mimic Extracellular Matrix for Skin Wound Healing. J. Mater. Sci..

[80] Kim S.H., An Y.H., Kim H.D., Kim K., Lee S.H., Yim H.G., Kim B.G., Hwang N.S. (2018). Enzyme-Mediated Tissue Adhesive Hydrogels for Meniscus Repair. Int. J. Biol. Macromol..

[81] Lin J.W., Jiang G.L., Liang C.X., Li Y.M., Chen X.Y., Zhang X.T., Tang Z.S. (2023). Laccase-Induced Gelation of Sugar Beet Pectin–Curcumin Nanocomplexes Enhanced by Genipin Crosslinking. Foods.

[82] Gantumur E., Sakai S., Nakahata M., Taya M. (2017). Cytocompatible Enzymatic Hydrogelation Mediated by Glucose and Cysteine Residues. ACS Macro Lett..

[83] Chen Y., Li Y., Yang X., Cao Z., Nie H., Bian Y., Yang G. (2021). Glucose-Triggered in Situ Forming Keratin Hydrogel for the Treatment of Diabetic Wounds. Acta Biomater..

[84] Rusu A.G., Nita L.E., Simionescu N., Ghilan A., Chiriac A.P., Mititelu-Tartau L. (2023). Enzymatically-Crosslinked Gelatin Hydrogels with Nanostructured Architecture and Self-Healing Performance for Potential Use as Wound Dressings. Polymers.

[85] Schmitt C., Turgeon S.L. (2011). Protein/Polysaccharide Complexes and Coacervates in Food Systems. Adv. Colloid Interface Sci..

[86] Alsaka L., Alsaka L., Altaee A., Zaidi S.J., Zhou J., Kazwini T. (2025). A Review of Hydrogel Application in Wastewater Purification. Separations.

[87] Moreno-Rivas S.C., Ibarra-Gutiérrez M.J., Fernández-Quiroz D., Lucero-Acuña A., Burgara-Estrella A.J., Zavala-Rivera P. (2024). PH-Responsive Alginate/Chitosan Gel Films: An Alternative for Removing Cadmium and Lead from Water. Gels.

[88] Zhou D., Zhang L., Guo S. (2005). Mechanisms of Lead Biosorption on Cellulose/Chitin Beads. Water Res..

[89] Song Y., Gotoh T., Nakai S. (2021). Synthesis of Oxidant Functionalised Cationic Polymer Hydrogel for Enhanced Removal of Arsenic (III). Gels.

[90] Zhang Z., Lu Y., Gao S., Wu S. (2025). Sustainable and Efficient Wastewater Treatment Using Cellulose-Based Hydrogels: A Review of Heavy Metal, Dye, and Micropollutant Removal Applications. Separations.

[91] Han Z., Wang N., Zhang H., Yang X. (2017). Heavy Metal Contamination and Risk Assessment of Human Exposure near an E-Waste Processing Site. Acta Agric. Scand. Sect. B Soil Plant Sci..

[92] Zhao H., Sun J., Du Y., Zhang M., Yang Z., Su J., Peng X., Liu X., Sun G., Cui Y. (2023). In-Situ Immobilization of CuMOF on Sodium Alginate/Chitosan/Cellulose Nanofibril Composite Hydrogel for Fast and Highly Efficient Removal of Pb2+ from Aqueous Solutions. J. Solid State Chem..

[93] Pandey S., Pande P.P., Chaurasiya A., Kashaudhan K., Chaudhary A. (2025). Development of Eco-Friendly Sodium Alginate-Based Hydrogel for the Effective Removal of Copper(II) and Cobalt(II) from Contaminated Water. Colloid Polym. Sci..

[94] Aoudi B., Boluk Y., Gamal El-Din M. (2022). Recent Advances and Future Perspective on Nanocellulose-Based Materials in Diverse Water Treatment Applications. Sci. Total Environ..

[95] Han S., Xie H., Zhang L., Wang X., Zhong Y., Shen Y., Wang H., Hao C. (2023). High-Performance Polyethylenimine-Functionalized Lignin/Silica Porous Composite Microsphere for the Removal of Hexavalent Chromium, Phosphate and Congo Red from Aqueous Solutions. Ind. Crops Prod..

[96] Jamwal P., Chauhan G.S., Kumar P., Kumari B., Kumar K., Chauhan S. (2023). A Study in the Synthesis of New Pinus Wallichiana Derived Spherical Nanocellulose Hydrogel and Its Evaluation as Malachite Green Adsorbent. Sustain. Chem. Pharm..

[97] Nguyen B.C., Truong T.M., Nguyen N.T., Dinh D.N., Hollmann D., Nguyen M.N. (2024). Advanced Cellulose-Based Hydrogel TiO2 Catalyst Composites for Efficient Photocatalytic Degradation of Organic Dye Methylene Blue. Sci. Rep..

[98] Shruthi S., Vishalakshi B. (2024). Development of Banana Pseudo Stem Cellulose Fiber Based Magnetic Nanocomposite as an Adsorbent for Dye Removal. Int. J. Biol. Macromol..

[99] Hussain S., Salman M., Al-Ahmary K.M., Ahmed M. (2025). Synthesis of Potential Adsorbent for Removal of Malachite Green Dye Using Alginate Hydrogel Nanocomposites. Int. J. Biol. Macromol..

[100] Yin X., Xu P., Wang H. (2024). Efficient and Selective Removal of Heavy Metals and Dyes from Aqueous Solutions Using Guipi Residue-Based Hydrogel. Gels.

[101] Cheng L., Lu Y., Li P., Sun B., Wu L. (2025). Metal–Organic Framework (MOF)-Embedded Magnetic Polysaccharide Hydrogel Beads as Efficient Adsorbents for Malachite Green Removal. Molecules.

[102] Gao H., Jiang J., Huang Y., Wang H., Sun J., Jin Z., Wang J., Zhang J. (2022). Synthesis of Hydrogels for Adsorption of Anionic and Cationic Dyes in Water: Ionic Liquid as a Crosslinking Agent. SN Appl. Sci..

[103] You R., He Z., Xue F., Ju S. (2025). Construction of Robust H_2_TiO_3_@PAM Hydrogel Ion-Sieve via in-Situ Polymerization for Li+ Adsorption. Surf. Interfaces.

[104] Ji K., Li R., Zhou H., Xia M., Sun F. (2025). Synthesis and Characterization of Calamus-Based Polyacrylamide Hydrogel for Heavy Metal Adsorption. Polym. Bull..

[105] You Y., Liu X., Yang W., Zhang X., Qin Z., Yin X. (2025). Thermosensitive Hydrogel from Amidoximated Bacterial Cellulose for Uranium Adsorption. Cellulose.

[106] Mohan R.N.V.J., Rayanoothala P.S., Sree R.P. (2024). Next-Gen Agriculture: Integrating AI and XAI for Precision Crop Yield Predictions. Front. Plant Sci..

[107] Kabato W., Getnet G.T., Sinore T., Nemeth A., Molnár Z. (2025). Towards Climate-Smart Agriculture: Strategies for Sustainable Agricultural Production, Food Security, and Greenhouse Gas Reduction. Agronomy.

[108] Skrzypczak D., Jarzembowski Ł., Izydorczyk G., Mikula K., Hoppe V., Mielko K.A., Pudełko-Malik N., Młynarz P., Chojnacka K., Witek-Krowiak A. (2021). Hydrogel Alginate Seed Coating as an Innovative Method for Delivering Nutrients at the Early Stages of Plant Growth. Polymers.

[109] Abdel Hakiem A.A., Abdel Moneam Y.K., Said H.M., Senna M.M.H. (2022). Radiation Induced Biodegradable Polymer Blends for Growth Promotion of Corn Plants. Polym. Renew. Resour..

[110] Jahan M., Nassiri Mahallati M. (2020). Can Superabsorbent Polymers Improve Plants Production in Arid Regions?. Adv. Polym. Technol..

[111] Dingley C., Cass P., Adhikari B., Daver F. (2024). Application of Superabsorbent Natural Polymers in Agriculture. Polym. Renew. Resour..

[112] Aravamudhan A., Ramos D.M., Nada A.A., Kumbar S.G. (2014). Natural Polymers: Polysaccharides and Their Derivatives for Biomedical Applications.

[113] Xu Y. (2017). Hierarchical Materials. Modern Inorganic Synthetic Chemistry.

[114] Aspinall G.O. (1983). Classification of Polysaccharides.

[115] Ghobashy M.M. (2019). The Application of Natural Polymer-Based Hydrogels for Agriculture.

[116] Song B., Liang H., Sun R., Peng P., Jiang Y., She D. (2020). Hydrogel Synthesis Based on Lignin/Sodium Alginate and Application in Agriculture. Int. J. Biol. Macromol..

[117] Yan J., Ye L., Tang W., Xia N., Long Z., Yan C., Zhao X. (2025). Development of Injectable Superabsorbent Resin with Wet Adhesion Based on P(AA-AM-AMPS) Copolymer for Agricultural Application. Eur. Polym. J..

[118] Chen P., Zhang W., Luo W., Fang Y. (2004). Synthesis of Superabsorbent Polymers by Irradiation and Their Applications in Agriculture. J. Appl. Polym. Sci..

[119] Wang Y., Zhao Y., Guo Y., Han W., Zhang Z., Hou T., Li H., Li H., Wang Q. (2024). Preparation of Perilla Frutescens L. Essential Oil Hydrogel Beads and Preservation Application Research in Strawberry. Heliyon.

[120] Calcagnile P., Sibillano T., Giannini C., Sannino A., Demitri C. (2019). Biodegradable Poly(Lactic Acid)/Cellulose-Based Superabsorbent Hydrogel Composite Material as Water and Fertilizer Reservoir in Agricultural Applications. J. Appl. Polym. Sci..

[121] Das D., Chingakham N., Sarma M., Basu S., Bhaladhare S. (2025). Cellulose-Based Biodegradable Superabsorbent Hydrogel: A Sustainable Approach for Water Conservation and Plant Growth in Agriculture. Int. J. Biol. Macromol..

[122] Rop K., Mbui D., Njomo N., Karuku G.N., Michira I., Ajayi R.F. (2019). Biodegradable Water Hyacinth Cellulose-Graft-Poly(Ammonium Acrylate-Co-Acrylic Acid) Polymer Hydrogel for Potential Agricultural Application. Heliyon.

[123] Pathak V., Ambrose R.P.K. (2020). Starch-Based Biodegradable Hydrogel as Seed Coating for Corn to Improve Early Growth under Water Shortage. J. Appl. Polym. Sci..

[124] Ghobashy M.M., Amin M.A., Mustafa A.E., El-diehy M.A., El-Damhougy B.K., Nady N. (2024). Synthesis and Application of a Multifunctional Poly (Vinyl Pyrrolidone)-Based Superabsorbent Hydrogel for Controlled Fertilizer Release and Enhanced Water Retention in Drought-Stressed Pisum Sativum Plants. Sci. Rep..

[125] Javaid M., Haleem A., Singh R.P., Suman R. (2023). Sustaining the Healthcare Systems through the Conceptual of Biomedical Engineering: A Study with Recent and Future Potentials. Biomed. Technol..

[126] Lim H.L., Hwang Y., Kar M., Varghese S. (2014). Smart Hydrogels as Functional Biomimetic Systems. Biomater. Sci..

[127] Hacker M.C., Nawaz H.A. (2015). Multi-Functional Macromers for Hydrogel Design in Biomedical Engineering and Regenerative Medicine. Int. J. Mol. Sci..

[128] Vo N.T.N., Huang L., Lemos H., Mellor A.L., Novakovic K. (2021). Genipin-Crosslinked Chitosan Hydrogels: Preliminary Evaluation of the in Vitro Biocompatibility and Biodegradation. J. Appl. Polym. Sci..

[129] Segneanu A.E., Bejenaru L.E., Bejenaru C., Blendea A., Mogoşanu G.D., Biţă A., Boia E.R. (2025). Advancements in Hydrogels: A Comprehensive Review of Natural and Synthetic Innovations for Biomedical Applications. Polymers.

[130] Lin C., Chen L., He Y., Xiang W., Nie Y., Cai B., Guo Z. (2024). Injectable, Self-Healing and Degradable Dynamic Hydrogels with Tunable Mechanical Properties and Stability by Thermal-Induced Micellization. RSC Adv..

[131] Nimmo C.M., Shoichet M.S. (2011). Regenerative Biomaterials That “Click”: Simple, Aqueous-Based Protocols for Hydrogel Synthesis, Surface Immobilization, and 3D Patterning. Bioconjug. Chem..

[132] Cho E., Lee J.S., Webb K. (2012). Formulation and Characterization of Poloxamine-Based Hydrogels as Tissue Sealants. Acta Biomater..

[133] Qin X.H., Ovsianikov A., Stampfl J., Liska R. (2014). Additive Manufacturing of Photosensitive Hydrogels for Tissue Engineering Applications. BioNanoMaterials.

[134] Zöller K., To D., Bernkop-Schnürch A. (2025). Biomedical Applications of Functional Hydrogels: Innovative Developments, Relevant Clinical Trials and Advanced Products. Biomaterials.

[135] Jo H., Yoon M., Gajendiran M., Kim K. (2020). Recent Strategies in Fabrication of Gradient Hydrogels for Tissue Engineering Applications. Macromol. Biosci..

[136] Gadjanski I. (2018). Recent Advances on Gradient Hydrogels in Biomimetic Cartilage Tissue Engineering. F1000Research.

[137] Xie W., Duan J., Li J., Qi B., Liu R., Yu B., Wang H., Zhuang X., Xu M., Zhou J. (2021). Charge-Gradient Hydrogels Enable Direct Zero Liquid Discharge for Hypersaline Wastewater Management. Adv. Mater..

[138] Xu P., Tan Y., Wang X., Xu H., Wang D., Yang Y., An W., Xu S. (2020). Multidimensional Gradient Hydrogel and Its Application in Sustained Release. Colloid Polym. Sci..

[139] Liu X., Liu S., Yang R., Wang P., Zhang W., Tan X., Ren Y., Chi B. (2021). Gradient Chondroitin Sulfate/Poly (γ-Glutamic Acid) Hydrogels Inducing Differentiation of Stem Cells for Cartilage Tissue Engineering. Carbohydr. Polym..

[140] Yasuda T. (2010). Hyaluronan Inhibits Prostaglandin E2 Production via CD44 in U937 Human Macrophages. Tohoku J. Exp. Med..

[141] Chen Y.T., Hou C.H., Hou S.M., Liu J.F. (2014). The Effects of Amphiregulin Induced Mmp-13 Production in Human Osteoarthritis Synovial Fibroblast. Mediat. Inflamm..

[142] Wei W., Li H., Yin C., Tang F. (2020). Research Progress in the Application of in Situ Hydrogel System in Tumor Treatment. Drug Deliv..

[143] Basuodan R., Basu A.P., Clowry G.J. (2018). Human Neural Stem Cells Dispersed in Artificial ECM Form Cerebral Organoids When Grafted in Vivo. J. Anat..

[144] Ma Y., Wang Y., Chen D., Su T., Chang Q., Huang W., Lu F. (2023). 3D Bioprinting of a Gradient Stiffened Gelatin-Alginate Hydrogel with Adipose-Derived Stem Cells for Full-Thickness Skin Regeneration. J. Mater. Chem. B.

[145] Patlay A.A., Belousov A.S., Silant’ev V.E., Shatilov R.A., Shmelev M.E., Kovalev V.V., Perminova I.V., Baklanov I.N., Kumeiko V.V. (2023). Preparation and Characterization of Hydrogel Films and Nanoparticles Based on Low-Esterified Pectin for Anticancer Applications. Polymers.

[146] Pereira R., Mendes A., Bártolo P. (2013). Alginate/Aloe Vera Hydrogel Films for Biomedical Applications. Procedia CIRP.

[147] Chang H.-C., Jørgensen B., Di Silvio L., Gurzawska-Comis K. (2023). 3D Bioprinted Pectin-Based Hydrogel as Sustainable Biomaterials for Musculoskeletal Tissue Engineering. Sustain. Mater. Technol..

[148] Chen W., Yuan S., Shen J., Chen Y., Xiao Y. (2021). A Composite Hydrogel Based on Pectin/Cellulose via Chemical Cross-Linking for Hemorrhage. Front. Bioeng. Biotechnol..

[149] Huang L., Gao J., Wang H., Xia B., Yang Y., Xu F., Zheng X., Huang J., Luo Z. (2020). Fabrication of 3D Scaffolds Displaying Biochemical Gradients along Longitudinally Oriented Microchannels for Neural Tissue Engineering. ACS Appl. Mater. Interfaces.

[150] Zhao X., Lang Q., Yildirimer L., Lin Z.Y., Cui W., Annabi N., Ng K.W., Dokmeci M.R., Ghaemmaghami A.M., Khademhosseini A. (2016). Photocrosslinkable Gelatin Hydrogel for Epidermal Tissue Engineering. Adv. Healthc. Mater..

[151] Borah R., O’Sullivan J., Suku M., Spurling D., Diez Clarke D., Nicolosi V., Caldwell M.A., Monaghan M.G. (2025). Electrically Conductive Injectable Silk/PEDOT: PSS Hydrogel for Enhanced Neural Network Formation. J. Biomed. Mater. Res. Part A.

[152] Zhai M., Huang Z., Xianyu B., Bai X., Wan Z., Fu Y., Xu H., Lv L., Zhou Y. (2025). Selenium-Containing Polyurethane Thermo-Sensitive Hydrogel Accelerates Diabetic Wound Healing by Activating Unfolded Protein Response. Aggregate.

[153] Ujah C.O., Kallon D.V.V., Aigbodion V.S. (2022). Overview of Electricity Transmission Conductors: Challenges and Remedies. Materials.

[154] Niu Y., Liu H., He R., Li Z., Ren H., Gao B., Guo H., Genin G.M., Xu F. (2020). The New Generation of Soft and Wearable Electronics for Health Monitoring in Varying Environment: From Normal to Extreme Conditions. Mater. Today.

[155] Dutta T., Chaturvedi P., Llamas-Garro I., Velázquez-González J.S., Dubey R., Mishra S.K. (2024). Smart Materials for Flexible Electronics and Devices: Hydrogel. RSC Adv..

[156] Wang J., Yang B., Jiang Z., Liu Y., Zhou L., Liu Z., Tang L. (2024). Recent Advances of Conductive Hydrogels for Flexible Electronics. Electron. Mater..

[157] Qin C., Lu A. (2021). Flexible, Anti-Freezing Self-Charging Power System Composed of Cellulose Based Supercapacitor and Triboelectric Nanogenerator. Carbohydr. Polym..

[158] Long Y., Bai M., Liu X., Lu W., Zhong C., Tian S., Xu S., Ma Y., Tian Y., Zhang H. (2022). A Zwitterionic Cellulose-Based Skin Sensor for the Real-Time Monitoring and Antibacterial Sensing Wound Dressing. Carbohydr. Polym..

[159] Xu Z., Zhou F., Yan H., Gao G., Li H., Li R., Chen T. (2021). Anti-Freezing Organohydrogel Triboelectric Nanogenerator toward Highly Efficient and Flexible Human-Machine Interaction at −30 °C. Nano Energy.

[160] Zhang D., Jian J., Xie Y., Gao S., Ling Z., Lai C., Wang J., Wang C., Chu F., Dumont M.-J. (2022). Mimicking Skin Cellulose Hydrogels for Sensor Applications. Chem. Eng. J..

[161] Zhao J., Wang H., Song X., Sun Y., Zhang X., Zheng J., Hu R. (2024). A Self-Healing and Recyclable Carrageenan Based Gel Based on Multi-Dynamic Interactions for Multifunctional Sensor. Nano Energy.

[162] Chen J., Peng Q., Thundat T., Zeng H. (2019). Stretchable, Injectable, and Self-Healing Conductive Hydrogel Enabled by Multiple Hydrogen Bonding toward Wearable Electronics. Chem. Mater..

[163] Segura Zarate A.Y., Gontrani L., Galliano S., Bauer E.M., Donia D.T., Barolo C., Bonomo M., Carbone M. (2024). Green Zinc/Galactomannan-Based Hydrogels Push up the Photovoltage of Quasi Solid Aqueous Dye Sensitized Solar Cells. Sol. Energy.

[164] Jiang D., Lu N., Li L., Zhang H., Luan J., Wang G. (2022). A Highly Compressible Hydrogel Electrolyte for Flexible Zn-MnO2 Battery. J. Colloid Interface Sci..

[165] Han C., Yang F., Guo X., Bai Y., Liu G., Sun H., Wang P., Liu W., Wang R. (2021). Ultra-Stretchable Self-Healing Composite Hydrogels as Touch Panel. Adv. Mater. Interfaces.

[166] Guo X., Yang F., Liu W., Han C., Bai Y., Sun X., Hao L., Jiao W., Wang R. (2021). Skin-Inspired Self-Healing Semiconductive Touch Panel Based on Novel Transparent Stretchable Hydrogels. J. Mater. Chem. A.

[167] Yin C., Wei F., Fu S., Zhai Z., Ge Z., Yao L., Jiang M., Liu M. (2021). Visible Light-Driven Jellyfish-like Miniature Swimming Soft Robot. ACS Appl. Mater. Interfaces.

[168] Luo X., Wan R., Zhang Z., Song M., Yan L., Xu J., Yang H., Lu B. (2024). 3D-Printed Hydrogel-Based Flexible Electrochromic Device for Wearable Displays. Adv. Sci..

[169] Yang C., Suo Z. (2018). Hydrogel Ionotronics. Nat. Rev. Mater..

[170] Liu Z., Wang Y., Ren Y., Jin G., Zhang C., Chen W., Yan F. (2020). Poly(Ionic Liquid) Hydrogel-Based Anti-Freezing Ionic Skin for a Soft Robotic Gripper. Mater. Horiz..

[171] Huang J., Zhang Z., Jiang H. (2023). Edible Hydrogels with Shrinkage Tolerance in Acids and Stomach-Friendly Mechanical Moduli. Appl. Mater. Today.

[172] Yang H., Yang H., Zhu C., Fan D., Deng J. (2023). Highly Expandable Edible Hydrogels for the Prevention and Treatment of Obesity through Dietary Intervention. Food Hydrocoll..

[173] Giannakas A.E., Zaharioudakis K., Kollia E., Kopsacheili A., Avdylaj L., Georgopoulos S., Leontiou A., Karabagias V.K., Kehayias G., Ragkava E. (2023). The Development of a Novel Sodium Alginate-Based Edible Active Hydrogel Coating and Its Application on Traditional Greek Spreadable Cheese. Gels.

[174] Cheng Y., Xu J., Zhang R., Lin J., Zhou M., Qin X., Wang K., Zhou Y., Zhu Q., Jin Y. (2024). Development of Multi-Cross-Linking, Rapid Curing, and Easy Cleaning, Edible Hydrogels for Meat Preservation. Food Hydrocoll..

[175] Wang K., Wu P., Hu Z., Chen X.D. (2025). PH-Sensitive, Edible Chitosan-Based Hydrogels with Extremely High Swelling and Reinforced Mechanics: A Chemical-Free Crosslinking Strategy for Gastric Retention. Food Hydrocoll..

[176] Le Y., Li H., Liao X., Wu Y., Zhang M., Jiang Y., Li L., Zhao W. (2024). Edible Hydrogel with Dual Network Structure for Weight Management. Food Res. Int..

[177] Gautam M., Santhiya D. (2019). In-Situ Mineralization of Calcium Carbonate in Pectin Based Edible Hydrogel for the Delivery of Protein at Colon. J. Drug Deliv. Sci. Technol..

[178] Keller A., Pham J., Warren H., In het Panhuis M. (2017). Conducting Hydrogels for Edible Electrodes. J. Mater. Chem. B.

[179] Kollia E., Kopsacheili A., Birlia C., Nikolaou D., Avdylaj L., Giannakas A.E., Salmas C.E., Tomasevic I., Brennan C., Proestos C. (2025). Chitosan-Zinc Oxide Thyme Oil-Based Edible Antimicrobial Hydrogel Coatings: Application on Pork and Tofu Sausages. Int. J. Food Sci. Technol..

[180] Pascuta M.S., Varvara R.A., Teleky B.E., Szabo K., Plamada D., Nemeş S.A., Mitrea L., Martău G.A., Ciont C., Călinoiu L.F. (2022). Polysaccharide-Based Edible Gels as Functional Ingredients: Characterization, Applicability, and Human Health Benefits. Gels.

[181] Zhang C., Shi X.-L., Liu Q., Chen Z.-G. (2024). Hydrogel-Based Functional Materials for Thermoelectric Applications: Progress and Perspectives. Adv. Funct. Mater..

[182] Yang P., Liu K., Chen Q., Mo X., Zhou Y., Li S., Feng G., Zhou J. (2016). Wearable Thermocells Based on Gel Electrolytes for the Utilization of Body Heat. Angew. Chem. Int. Ed..

[183] Sun S., Shi X., Lyu W., Hong M., Chen W., Li M., Cao T., Hu B., Liu Q., Chen Z. (2024). Graphene-Oxide-Modified Flexible Ionogel Thermoelectric Films. Adv. Funct. Mater..

[184] Zhang C., Nayeem M.O.G., Wang Z., Pu X., Dagdeviren C., Wang Z.L., Zhang X., Liu R. (2023). Conductive Hydrogels for Bioenergy Harvesting and Self-Powered Application. Prog. Mater. Sci..

[185] Wang Y., Hong M., Liu W.D., Shi X.L., Xu S.D., Sun Q., Gao H., Lu S., Zou J., Chen Z.G. (2020). Bi0.5Sb1.5Te3/PEDOT:PSS-Based Flexible Thermoelectric Film and Device. Chem. Eng. J..

[186] Liang Y., Wang K., Li J., Wang H., Xie X., Cui Y., Zhang Y., Wang M., Liu C. (2021). Low-Molecular-Weight Supramolecular-Polymer Double-Network Eutectogels for Self-Adhesive and Bidirectional Sensors. Adv. Funct. Mater..

[187] Zhang C., Wang M., Jiang C., Zhu P., Sun B., Gao Q., Gao C., Liu R. (2022). Highly Adhesive and Self-Healing γ-PGA/PEDOT:PSS Conductive Hydrogels Enabled by Multiple Hydrogen Bonding for Wearable Electronics. Nano Energy.

[188] Yang X., Tian Y., Wu B., Jia W., Hou C., Zhang Q., Li Y., Wang H. (2022). High-performance Ionic Thermoelectric Supercapacitor for Integrated Energy Conversion-storage. Energy Environ. Mater..

[189] Sun S., Li M., Shi X.L., Chen Z.G. (2023). Advances in Ionic Thermoelectrics: From Materials to Devices. Adv. Energy Mater..

[190] Kong S., Huang Z., Hu Y., Jiang Y., Lu Y., Zhao W., Shi Q., Yuan M., Dai B., Li J. (2023). Tellurium-Nanowire-Doped Thermoelectric Hydrogel with High Stretchability and Seebeck Coefficient for Low-Grade Heat Energy Harvesting. Nano Energy.

[191] Wu B., Geng J., Lin Y., Hou C., Zhang Q., Li Y., Wang H. (2022). Hydrogel-Based Printing Strategy for High-Performance Flexible Thermoelectric Generators. Nanoscale.

[192] Liu J., Jia Y., Jiang Q., Jiang F., Li C., Wang X., Liu P., Liu P., Hu F., Du Y. (2018). Highly Conductive Hydrogel Polymer Fibers toward Promising Wearable Thermoelectric Energy Harvesting. ACS Appl. Mater. Interfaces.

[193] Hsu C., Lin Y., Hong S., Jeng U., Chen H., Yu J., Liu C. (2024). 3D Printed Gelatin Methacrylate Hydrogel-Based Wearable Thermoelectric Generators. Adv. Sustain. Syst..

